# Canine distemper virus N protein induces autophagy to facilitate viral replication

**DOI:** 10.1186/s12917-023-03575-7

**Published:** 2023-03-15

**Authors:** Fei Chen, Zijing Guo, Rui Zhang, Zhixiong Zhang, Bo Hu, Ling Bai, Shuaiyang Zhao, Yongshu Wu, Zhidong Zhang, Yanmin Li

**Affiliations:** 1 State Key Laboratory of Veterinary Etiological Biology, Lanzhou Veterinary Research Institute, Chinese Academy of Agricultural Sciences, 1 Xu Jiaping, Lanzhou, 730046 Gansu China; 2 College of Animal Husbandry and Veterinary Medicine, Southwest Minzu University, 16 Yihuan Rd., Chengdu, 610041 Sichuan China; 3 Key Laboratory of Special Animal Epidemic Disease, Ministry of Agriculture, Institute of Special Animal and Plant Sciences, Chinese Academy of Agricultural Sciences, 4899 Juye St., Changchun, 130112 Jilin China

**Keywords:** Canine distemper virus, Autophagy, Replication, Nucleocapsid protein

## Abstract

**Background:**

Canine distemper virus (CDV) is one of the most contagious and lethal viruses known to the *Canidae*, with a very broad and expanding host range. Autophagy serves as a fundamental stabilizing response against pathogens, but some viruses have been able to evade or exploit it for their replication. However, the effect of autophagy mechanisms on CDV infection is still unclear.

**Results:**

In the present study, autophagy was induced in CDV-infected Vero cells as demonstrated by elevated LC3-II levels and aggregation of green fluorescent protein (GFP)-LC3 spots. Furthermore, CDV promoted the complete autophagic process, which could be determined by the degradation of p62, co-localization of LC3 with lysosomes, GFP degradation, and accumulation of LC3-II and p62 due to the lysosomal protease inhibitor E64d. In addition, the use of Rapamycin to promote autophagy promoted CDV replication, and the inhibition of autophagy by Wortmannin, Chloroquine and siRNA-ATG5 inhibited CDV replication, revealing that CDV-induced autophagy facilitated virus replication. We also found that UV-inactivated CDV still induced autophagy, and that nucleocapsid (N) protein was able to induce complete autophagy in an mTOR-dependent manner.

**Conclusions:**

This study for the first time revealed that CDV N protein induced complete autophagy to facilitate viral replication.

**Supplementary Information:**

The online version contains supplementary material available at 10.1186/s12917-023-03575-7.

## Background

Canine distemper virus (CDV) can infect *Canidae* causing canine distemper (CD), one of the most contagious diseases in the *Canidae*, which can result in high morbidity and mortality in unimmunized host [1]. CDV has multicellular tropism (epithelial cells, lymphocytes, and nerve cells) and can cause systemic infections. [2]. The clinical symptoms of CD characterized by fever, increased ocular and nasal discharge, conjunctivitis, diarrhea, vomiting, rash, lymphopenia, and neurologic symptoms such as encephalitis, with severe immunosuppression, but also lifelong immunity in surviving hosts [3–6]. CDV has an expanding host range, mainly comprising mammals from at least 6 orders and more than 20 families [4, 7], especially all families of *Carnivora* [8, 9]. It poses a great threat to the pet industry, fur industry, and conservation of endangered species worldwide [10], especially the recent large outbreaks of CD in primate populations of Macaca mulatta and Macaca fascicularis, which have raised concerns about possible zoonotic diseases and public health concerns after measles eradication [11, 12]. CDV is a single-stranded, negative-stranded, non-segmented RNA virus, which belongs to the *Morbillivirus* genus of the *Paramyxoviridae* family [13, 14]. The viral particles have a double-layered envelope with dense spikes. The full-length genome is 15.69 kb and contains 6 transcription units (N-P-M-F–H-L), and 8 proteins are encoded, of which the P gene encoding two non-structural proteins C and V [15].

Autophagy is an evolutionarily conserved catabolic process that begins with the isolation of "cargo" (unwanted proteins, damaged organelles, invading microorganisms, etc.) to be degraded into autophagosome with a double membrane, which then fuse with lysosome to form autophagolysosome, in which hydrolytic enzymes degrade the luminal contents, and metabolic byproducts can be reused to maintain the dynamic balance of the intracellular environment [16, 17]. Autophagy (programmed cell death type II) also occurs at basal levels in normal cells as a cytoprotective mechanism [18, 19]. Autophagy can also be up- or down-regulated in response to various stress stimuli both inside and outside the cell, such as nutrient deficiency, oxidative stress, endoplasmic reticulum stress, pathogen infection, and pathogen-associated molecular patterns [20, 21]. In a process known as “xenophagy”, the isolation of viral particles within the autophagosome may lead to their degradation and destruction and/or delivery to compartments involved in pathogen recognition and antigen presentation to activate immune responses against the virus [22]. On the other hand, viruses have evolved strategies to evade or hijack autophagy to facilitate their own propagation, and viruses can use autophagosomal membranes as replication platforms or interact with autophagy-related-gene (ATG) proteins to aid in the assembly, maturation, and encapsulation of viral particles [23–25]. Measles virus (MeV) [26], peste des petits ruminants virus (PPRV) [27], newcastle disease virus (NDV), hepatitis C virus (HCV) [28], foot-and-mouth disease virus (FMDV) [29, 30], and Influenza A virus [31] use the autophagic process for replication.

The mammalian target of rapamycin (mTOR) signaling pathway is a key pathway regulating cell growth, proliferation and survival [32]. mTOR is a serine/threonine kinase belonging to the phosphatidylinositol 3-kinase (PI3K)-related protein kinase family, and the PI3K/protein kinase B (PKB, generally known as AKT) pathway is the main upstream regulator of mTORC1, whose activation inhibits autophagy [32, 33]. The relationship between other members of the *Paramyxoviridae* family and autophagy has been studied. Previous study found that the number of stained cells and the intensity of the immunoreaction for autophagy-associated microtubule-associated protein 1 light chain 3 (LC3) were significantly elevated in the cerebellums of CDV-infected dogs, which indicated that autophagy increased in virus-infected cells [34]. Another study showed that CDV induced virus-cell or cell–cell fusion triggering autophagy which in turn enhanced cell-to-cell transmission of the virus [35]. Both studies suggest that CDV infection induces the onset of autophagy, but the impact of the autophagy machinery on CDV infection remains unclear. In this study, the relationship between CDV and autophagy was systematically investigated for the first time. The data provided the first evidence that CDV induced complete autophagic flux to promote viral replication. For the first time, it was revealed that nucleocapsid protein induced complete autophagy in an mTOR-dependent manner and played an important role in CDV-induced autophagy to facilitate viral replication.

## Results

### Infection of CDV triggers autophagy in vero cells

**Fig. 1:**
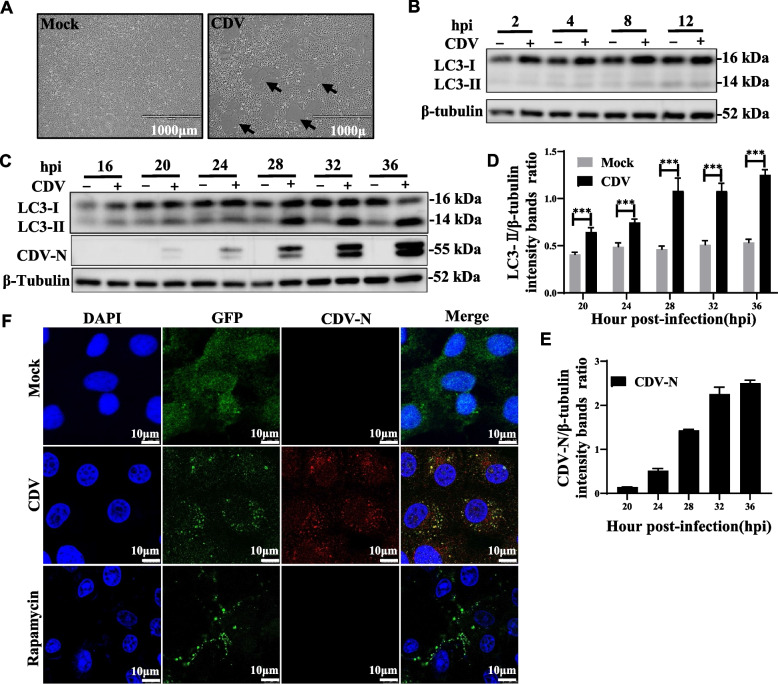
CDV infection triggers autophagy in vero cells. **A** The cytopathic effect (CPE) in CDV-infected Vero cell at 28 hpi (magnification, × 4). **B** and **C** Vero cells were mock-infected or infected with CDV (MOI = 1) for 2, 4, 8, 12, 16, 20, 24, 28, 32, and 36 h. The expression levels of LC3B, CDV-N, and β-tubulin (loading control) were analyzed by western blot with specific antibodies. **D** The LC3-II levels relative to the β-tubulin levels were determined by densitometry. **E** The CDV-N levels relative to the β-tubulin levels were determined by densitometry. **F** Vero cells were transfected with GFP-LC3 for 28 h. The cells were then fixed and processed for indirect immunofluorescence using antibodies against CDV-N protein, followed by the corresponding secondary antibodies. The fluorescence signals were visualized by confocal immunofluorescence microscopy. The blots were cropped. The samples derived from the same experiment and that blots were processed in parallel. The data represent the mean ± SD of three independent experiments. Two-way ANOVA; ***P < 0.001

The CDV3-CL strain can infect Vero cells and passage on Vero cells, resulting in syncytial lesions (Fig. 1A). The microtubule-associated protein 1 light chain 3β (MAP1LC3B/LC3B) was known to bind to mature autophagosome membranes and was a marker of autophagosome formation [36, 37]. The results showed that the amount of LC3-II increased with the progression of CDV infection (Fig. 1B, C), and the level of LC3-II were found to increase starting at 20 hpi, consistent with the level of N protein (Fig. 1C, E). Because the amount of LC3-II was closely related to the number of autophagosomes, the ratio of LC3-II to β-tubulin in cells was now considered to be an accurate indicator of autophagic activity [38]. The gray scale ratio of LC3-II to β-tubulin bands was higher in CDV-infected Vero cells than that in mock-infected cells from 20 hpi onwards (Fig. 1D). Furthermore, we next performed immunofluorescence assays. Consistent with the results with immunoblotting, a large amount of punctate GFP-LC3 protein was observed in or near the syncytium induced by viral infection, and similarly, in Rapamycin-treated positive control cells. In contrast, mock-infected Vero cells exhibited a weak diffuse staining pattern with almost no LC3 punctate accumulation (Fig. 1F). These results suggested that CDV infection induced the onset of autophagy in Vero cells.

### CDV infection enhances autophagic flux

**Fig. 2:**
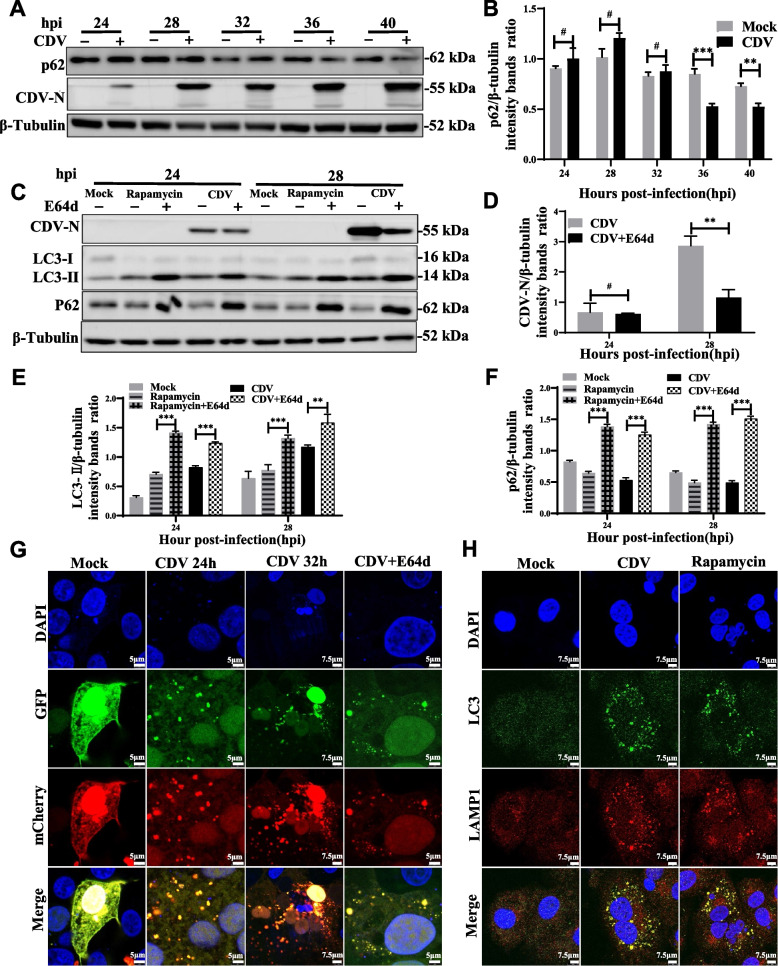
CDV infection enhances autophagic flux. **A** Vero cells were mock-infected or infected with CDV (MOI = 1) for 24, 28, 32, 36 h and 40 h. The expression levels of p62/SQSTM1, CDV-N, and β-tubulin (loading control) were analyzed by western blot with specific antibodies. **B** The p62 levels relative to the β-tubulin levels were determined by densitometry. **C** Vero cells pre-treated with rapamycin for 4 h and then mock-infected with DMEM, and cells infected with CDV (MOI = 1) were further cultured in the absence and presence of 10 mg/mL E64d for 24 and 28 h. The cell samples were then analyzed by western blot with anti-CDV-N, anti-LC3B, anti-p62, and anti-β-tubulin (loading control) antibodies. **D**, **E** and **F** The CDV-N, LC3B and p62 levels relative to the β-tubulin levels were determined by densitometry in mock-infected, rapamycin-pre-treated, and CDV-infected Vero cells in the absence and presence of E64d. **G** Cells were transfected with pmCherry-GFP-LC3B for 24 h, followed by CDV infection (MOI = 1) and treatment with E64d. The fluorescence signals were visualized by confocal immunofluorescence microscopy. **H** Vero cells were mock-infected or infected with CDV (MOI = 1) for 28 h. The cells were fixed and processed for indirect immunofluorescence using antibodies against LC3 and LAMP1 protein. The blots were cropped. The samples derived from the same experiment and that blots were processed in parallel. The data represent the mean ± SD of three independent experiments. Two-way ANOVA; # *P* > 0.05; **P < 0.01; ***P < 0.001

To investigate whether CDV-induced autophagy is a complete process, we determined the degradation of polyubiquitin-binding protein p62/SQSTM1 (sequestosome 1), a marker of autophagy-mediated protein degradation pathway, which is considered as a marker of autophagic flux [37]. As shown in the Fig. 2A, the level of p62 protein did not change significantly during the mid to late phase of infection (from 24 to 32 hpi) when compared to mock cells, but decreased significantly at late stage of the infection (from 36 to 40 hpi), and similarly, the gray scale ratio of p62 protein to β-tubulin bands in CDV-infected Vero cells was much lower than that in negative control cells (Fig. 2B). E64d is a membrane-permeable inhibitor of histones B, H and L. The upregulation of LC3-II and p62 in the presence of the lysosomal protease inhibitor represents an increase in autophagic flux. Both LC3-II and p62 degradation were significantly inhibited in CDV-infected cells treated E64d. The same results were also obtained from E64d and Rapamycin-treated Vero cells (Fig. 2C-F).

To further confirm the above results, Vero cells were transfected with the dual fluorescent-tagged plasmid mCherry-GFP-LC3B. The green signaling protein (GFP) is degraded and quenched under low pH conditions in the lumen of the lysosome, whereas the red signaling protein (mCherry) exhibits more stable fluorescence under acidic conditions [37, 39]. As shown in Figs. 2G and S1A, almost all of the green and red fluorescent puncta colocalized in the CDV-infected Vero cells at 24 hpi. In contrast, the number and intensity of the red fluorescent dots are greater than the green fluorescent point at 32 hpi (Figs. 2G, S1B). Subsequently, E64d treatment dramatically recovered green fluorescent puncta and increased yellow puncta in CDV-infected Vero cells (Figs. 2G and S1C). These results showed that CDV infection could promote GFP to be degraded by autolysosome. Lysosome-associated membrane protein 1 (LAMP1), a lysosomal marker, co-localizes with LC3 during autophagosome maturation [40]. There was a punctiform distribution of LC3 with LAMP1 in CDV-infected cells. More importantly, partial overlap of LC3 with LAMP1 was observed, and the same phenomenon was observed in Rapamycin-treated positive control cells. In contrast, both LC3 and LAMP1 exhibited weaker diffuse staining in negative control cells (Fig. 2H). In summary, CDV infection increased autophagosome formation, promoted the fusion of autophagosome with lysosome to form autophagosome, enhanced autophagic degradation, and enhanced autophagic flux.

### Induction of autophagy with rapamycin promotes virus replication

**Fig. 3:**
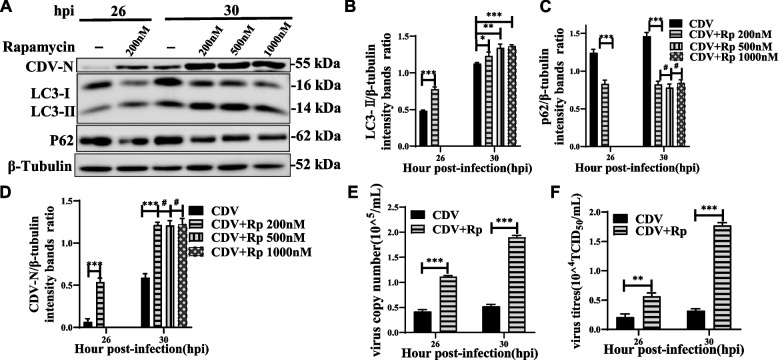
Induction of autophagy with rapamycin promotes virus replication. **A** Vero cells were pre-treated with rapamycin for 4 h and then infected with CDV (MOI = 1) for 26 and 30 h. The cell samples were then analyzed by western blot with anti-CDV-N, anti-LC3B, anti-p62, and anti-β-tubulin (loading control) antibodies. **B**, **C** and **D** The CDV-N, LC3B and p62 levels relative to the β-tubulin levels were determined by densitometry. **E** and **F** Vero cells were pre-treated and infected as described in (**A**). At 26 and 30 hpi, copy numbers of CDV were measured by qRT-PCR; virus titers were measured by TCID_50_. The blots were cropped. The samples derived from the same experiment and that blots were processed in parallel. The data represent the mean ± SD of three independent experiments. Two-way ANOVA; # *P* > 0.05; *P < 0.05; **P < 0.01; ***P < 0.001

Treatment of cells with the autophagy inducer rapamycin did induce autophagy, which not only upregulated the expression of LC3-II in CDV-infected Vero cells (Fig. 3A, B), but also decreased the expression of p62 (Fig. 3A, C). Importantly, rapamycin treatment increased the level of CDV N protein in a non-dose dependent manner (Fig. 3A, D). In addition, the effect of rapamycin treatment was also found to significantly increase viral RNA copy numbers and viral titer (Fig. 3E, F). These findings suggested that the autophagy contributed to CDV replication.

### Inhibition of autophagy reduces CDV replication

**Fig. 4:**
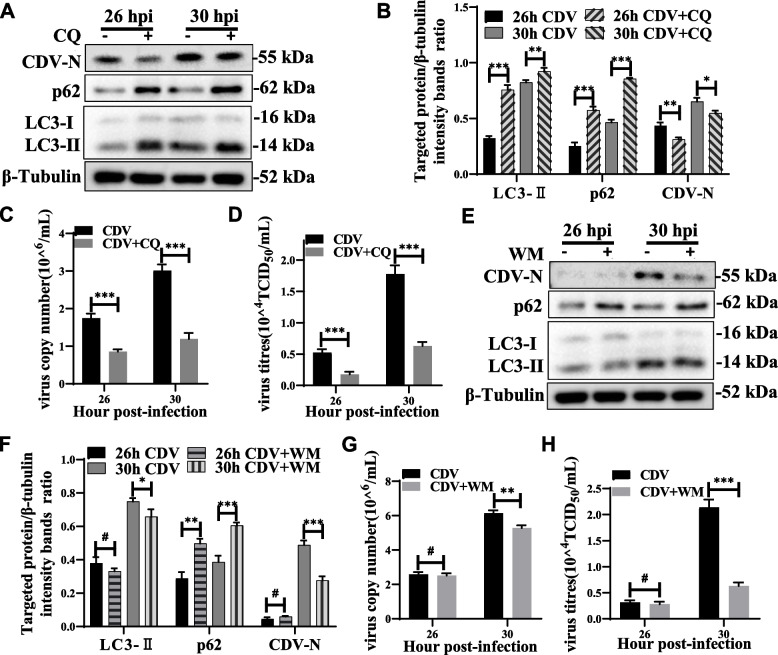
Autophagy inhibition reduces CDV replication. **A** and **E** Vero cells were pre-treated with Chloroquine and Wortmannin for 4 h and then infected with CDV (MOI = 1) for 26 and 30 h. The cell samples were then analyzed by western blot with anti-CDV-N, anti-LC3B, anti-p62, and anti-β-tubulin (loading control) antibodies. **B** and **F** Vero cells were pre-treated and infected as described in (**A** and **E**). At 26 and 30 hpi, the target protein levels relative to the β-tubulin levels were determined by densitometry in control, chloroquine- and wortmannin-pre-treated cells. **C-D** and **G-H** Vero cells were pre-treated and infected as described in (**A** and **E**). At 26 and 30 hpi, copy numbers of CDV were measured by qRT-PCR; virus titers were measured by TCID_50_. The blots were cropped. The samples derived from the same experiment and that blots were processed in parallel. The data represent the mean ± SD of three independent experiments. Two-way ANOVA; # *P* > 0.05; *P < 0.05; **P < 0.01; ***P < 0.001

To further confirm the effect of autophagy on CDV replication, we treated Vero cells with chloroquine (CQ) and wortmannin (WM) and examined CDV replication. CQ can inhibit endosomal acidification, causing accumulation of autophagosomes and autophagolysosomal contents, and inhibits autophagy at a late stage [41]. Wortmannin, a specific inhibitor of the PI3K pathway, blocks autophagy at an early stage [42]. In CDV-infected Vero cells, chloroquine treatment increased the accumulation of LC3-II and p62 (Fig. 4A, B), and Wortmannin treatment decreased LC3-II expression and p62 degradation (Fig. 4E, F). Importantly, The CDV-N expression levels were reduced after treatment of cells with CQ and WM (Fig. 4A, B, E, F). Similarly, Chloroquine and Wortmannin treatment resulted in a significant reduction in viral RNA levels and titers (Fig. 4C, D, G, H). In addition, E64d treatment inhibited autophagic degradation, resulting in reduction of CDV N protein expression levels (Fig. 2C, D). These data suggested that pharmacological inhibition of autophagy negatively regulated CDV replication.

**Fig. 5:**
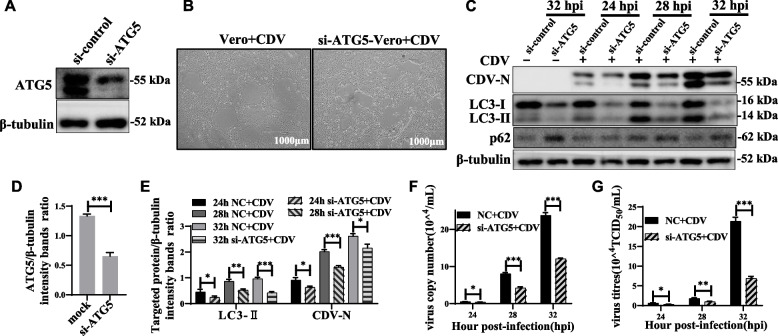
Inhibition of autophagy with specific siRNA targeting ATG5 reduces CDV replication. **A** and **C** Vero cells were transfected with siRNA-ATG5 or scrambled siRNA for 48 h; then, the cells were infected with CDV (MOI = 1). The cell samples were then analyzed by western blot with anti-ATG5, anti-CDV-N, anti-LC3B, and anti-β-tubulin (loading control) antibodies. **B** Cell lesions in CDV-infected Vero and siRNA-ATG5-Vero cell at 32 hpi (magnification, × 4). **D**-**E** Vero cells were pre-treated and infected as described in (**C**). At 24, 28 and 32 hpi, the target protein levels relative to the β-tubulin levels were determined by densitometry. **F**-**G** Vero cells were pre-treated and infected as described in (**C**). At 24, 28 and 32 hpi, copy numbers of CDV were measured by qRT-PCR; virus titers were measured by TCID_50_. The blots were cropped. The samples derived from the same experiment and that blots were processed in parallel. The data represent the mean ± SD of three independent experiments. Two-way ANOVA; *P < 0.05; **P < 0.01; ***P < 0.001

We next examined the effect of target-specific RNA interference with reduced expression of the endogenous autophagy-associated protein ATG5 on CDV replication. The ATG5 protein, with ATG12 and ATG16L1, forms a conjugation system that is essential for the phagophore elongation process. Knockdown of ATG5 can block the autophagy process [43]. As shown in the Fig. 5A, C and D, Vero cells transfected with ATG5-specific small interfering RNA (si-ATG5) exhibited significantly lower levels of endogenous ATG5 and LC3-II protein compared to cells transfected with scrambled siRNA, indicating that the expression of the target protein was successfully suppressed in Vero cells. There was no significant difference in the effect of syncytial lesions caused by CDV infection of the two cell types (Fig. 5B). Importantly, ATG5 knockdown significantly inhibited CDV-induced autophagy (Fig. 5C, E). These results suggested that CDV infection can induce autophagy in Vero cells through an ATG5-dependent pathway. More importantly, inhibition of autophagy strongly reduced CDV N protein expression (Fig. 5C, E), viral RNA copy number and viral titer (Fig. 5F, G). These data further revealed that inhibition of autophagy at the genetic level is detrimental to CDV replication.

### CDV N proteins induce autophagy in an mTOR-dependent manner

**Fig. 6:**
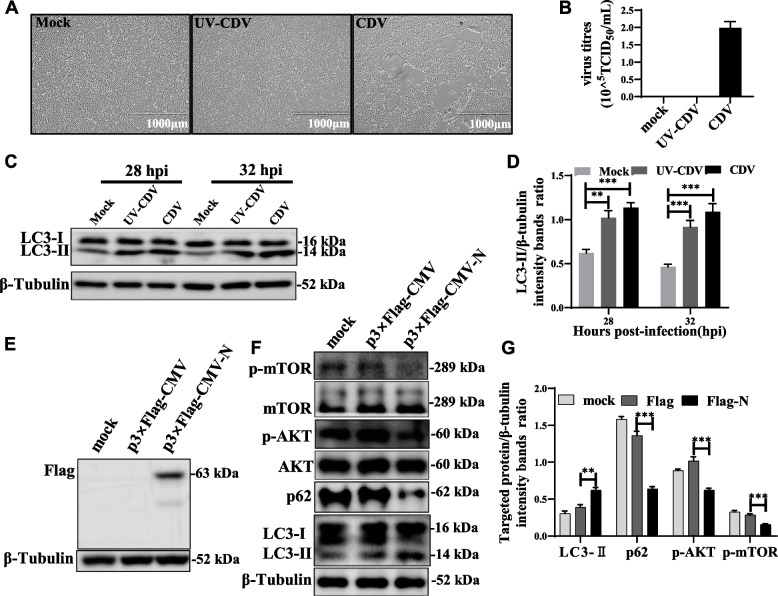
N Proteins Induce Autophagy in an mTOR-Dependent Manner. **A** The CPE in mock-, UV-CDV-, CDV-infected Vero cell at 72 hpi (magnification, × 4). **B** Vero cells were infected as described in (**A**). virus titers were measured by TCID_50_. **C** Vero cells were infected as described in (**A**). The cell samples were then analyzed by western blot with anti-CDV-N, anti-LC3B, and anti-β-tubulin (loading control) antibodies. **D** The LC3B levels relative to the β-tubulin levels were determined by densitometry. **E** Vero cells were transfected with p3 × Flag-CMV and p3 × Flag-CMV-N plasmids expressing the fusion protein for 48 h. The cell samples were then analyzed by western blot with anti-Flag antibody. **F** Vero cells were infected as described in (**E**). The cell samples were then analyzed by western blot with anti-p-mTOR, anti-mTOR, anti-p-AKT, anti-AKT, anti-p62, anti-LC3B and anti-β-tubulin (loading control) antibodies. **G** Vero cells were infected as described in (**E**). The target protein levels relative to the β-tubulin levels were determined by densitometry. The blots were cropped. The samples derived from the same experiment and that blots were processed in parallel. The data represent the mean ± SD of three independent experiments. Two-way ANOVA; **P < 0.01; ***P < 0.001

We inactivated CDV by ultraviolet (UV) irradiation [28]. Infectious CDV could infect cells producing significant syncytial lesions and viral titers, whereas the cells treated with UV-inactivated CDV after 72 hpi produced none (Fig. 6A, B). These data suggested that UV-inactivated CDV had lost its ability to infect and replicate. More importantly, both infectious CDV and UV-inactivated CDV inoculated Vero cells increased the expression level of LC3-II compared to mock-infected cells (Fig. 6C, D). This indicated that UV-inactivated CDV can also induce the onset of autophagy. Therefore, structural proteins of CDV may play an important role in the process of virus-induced autophagy.

To analyze the effect of different viral proteins of CDV on autophagy induction. We constructed expression vectors with flag-tagged proteins and transfected them with Vero cells. Western blot assays revealed that Flag-N, Flag-P, Flag-V, Flag-C proteins were expressed in Vero cells (Figs. 6E, S2A). When we transfected the same concentration of plasmids, Flag-N caused the LC3-II level to increase the most significantly (Fig. S2B). Additionally, Flag-N promoted the degradation of p62 protein (Fig. 6F, G). This suggested that CDV N protein induces complete autophagic flux. The mammalian target of rapamycin (mTOR) is an important signaling molecule that negatively regulates autophagy and is also a downstream target of phosphatidylinositol 3 kinase (PI3K) and AKT pathways [44]. The expression of phosphorylated AKT (Ser 473) and phosphorylated mTOR (Ser 2448) was reduced in Vero cells expressing Flag-N protein compared to controls, but there was no significant change in total AKT or mTOR expression (Fig. 6F, G). These data suggested that CDV N protein induced autophagy by inhibiting the AKT-mTOR pathway, indicating that N protein play an important role in CDV-induced autophagy.

## Discussion

Autophagy relies on lysosomal degradation and plays an important role in maintaining intracellular homeostasis by recycling long-lived proteins and obsolete or damaged organelles during nutrient deficiency, energy recycling, and cell survival. Subsequently, it has been shown that autophagy can also play an important role in defense against pathogens as an immune mechanism after pathogen invasion of cells [45, 46]. Although autophagy can transport viruses to the lysosomal compartment for degradation [47], it has been shown that sindbis virus, tobacco mosaic virus and vesicular stomatitis virus infection-induced autophagy exerts a protective function by limiting intracellular virus replication [48–50]. However, an increasing number of pathogens have evolved strategies to avoid or hijack autophagy for their own benefit. Members of the *Paramyxoviridae* family have been shown to induce autophagy in target cells, including MeV [26], PPRV [27], NDV [51], sendai virus (SeV) [52], human parainfluenza virus type 3 (HPIV3) [53], human respiratory syncytial virus (HRSV) [54], and simian parainfluenza virus 5 (SV5) [55]. In this study, the date suggested that CDV infection of Vero cells led to increased autophagosome formation and induced autophagy. Consistent with the results of our study, the number of GFP-LC3 spots increased and autophagosome accumulation increased after infection of GFP-LC3-transfected VerodogSLAMtag cells by CDV wild-type strain 5804P and MeV vaccine strain MeVvac [35]. Application of immunohistochemistry to detect the expression of microtubule-associated protein 1 light chain 3 (LC3) protein showed increased autophagy in CDV-infected canine cerebellums [34].

Autophagy is a dynamic process and the accumulation of LC3 dot may be the result of increased autophagosome formation or decreased autophagosome degradation [36]. Many date have recently shown that viruses can induce the early stages of autophagy but prevent the fusion of autophagosomes with lysosomes, such as coxsackievirus B3 (CVB3) [56], herpes simplex virus [57], influenza A virus [58], porcine reproductive and respiratory syndrome virus [59] and hepatitis C virus [60]. MeV [26], PPRV [27], NDV [51], SeV [51], and RSV [54], members of the *Paramyxoviridae* family, induce increased autophagic flux, whereas HPIV3 induces incomplete autophagy by blocking autophagosome-lysosome fusion [53]. In this study, we found that CDV infection induced complete autophagy and increased autophagic flux. The previous study showed that CDV or MeV infection of VerodogSLAMtag cells induced the formation of autophagosome, but prevented their subsequent fusion with lysosomes [35]. Another study showed no change in the intensity of LC3 expression in cerebellar tissue during the chronic phase of CDV infection as in the acute phase, suggesting that the virus delayed or prevented the degradation of autophagosome [34].

Several members of the *Paramyxoviridae* family, including MeV [26], HRSV [54], PPRV [26], NDV [51], and HPIV3 [53], can promote replication using an autophagic mechanism. Kabak et al. suggested that CDV does not use autophagy for replication and supported the suggestion by Delpeut et al. that CDV induces autophagy through virus-cell and cell–cell fusion and that autophagy enhances cell-to-cell transmission of morbilliviruses rather than replication [34, 35]. Our data showed that both pharmacological and genetic promotion and inhibition of autophagy were important for CDV replication in Vero cells. The reason for the difference between the results of the above study and our study may be the difference in the CDV strains used and the infected cells. This also suggests that the autophagic response is virus and cell type specific and even related to cellular pathogen receptor. It has been shown then that infection of different cells with the same rabies virus strain induces different autophagic flux responses [61].

mTOR signaling is essential for many cellular processes such as growth, survival, and proliferation [32]. As a major autophagy regulator, mTOR negatively regulates autophagy when activated by signals from nutrients, growth factors, and stress stimuli [62]. Among them, the PI3K/Akt/mTOR signaling pathway plays a key role in autophagy [33]. PPRV [27], CVB3 [63], FMDV [29], Avian influenza H5N1 virus and avian reovirus trigger autophagy by inhibiting AKT-mTOR signaling [64, 65]. Our data suggested that CDV N protein induced autophagy by inhibiting the AKT-mTOR pathway, suggesting that N protein plays an important role in CDV-induced autophagy for viral replication. The nucleoprotein of MeV and the cytoplasmic nucleocapsid of CDV interact with 70 K heat shock proteins (HSP70K) [66, 67]. PPRV-N interacts with HSPA1A to play an important role in PPRV-induced second autophagic wave [27]. Which key nodal proteins interact with CDV proteins to affect autophagy deserve further investigation.

## Conclusions

This study is the first systematic investigation of the relationship between autophagy and CDV replication. For the first time, it was demonstrated that CDV induces complete autophagic flux in Vero cells to promote viral replication. It was revealed for the first time that nucleocapsid protein induces complete autophagy in an mTOR-dependent manner, which plays an important role in CDV-induced autophagy to facilitate viral replication. Given that autophagy facilitates CDV replication, our study reveals a novel approach to improve the efficiency of live attenuated CDV vaccine production that may enhance its immune effect in host cells by targeting the autophagic pathway. Our study also provides novel mechanisms of CDV-cell interactions and may provide theoretical references for the development of strategies against CDV infection and CDV-host cell interactions.

## Materials and methods

### Antibodies, reagents and plasmids

The primary antibodies used in this study included antibodies against rabbit LC3B (Abcam, UK); SQSTM1/p62, Phospho-mTOR (Ser2448), Phospho-Akt (Ser473), AKT (all from Cell Signaling Technology, USA); LAMP1 (Proteintech, USA), ATG5 (Novus Biologicals, USA); mouse mTOR (Proteintech, USA); β-Tubulin (Affinity, USA); CDV-NP (VMRD, USA). Secondary antibodies were goat anti-mouse IgG-HRP, goat anti-rabbit IgG-HRP antibody (Bioworld, USA); Alexa Fluor 488 goat anti-rabbit IgG, Alexa Fluor 594 goat anti-mouse IgG (Invitrogen, USA); Alexa Fluor 594 goat anti-rabbit IgG (Abmart, China). Pharmaceuticals were Rapamycin, Wortmannin, E64d (Selleck, USA); Chloroquine (Sigma-Aldrich, USA). Plasmids pEGFP-LC3B (Cat.: P0199) and pmCherry-EGFP-LC3B (Cat.: P0446) was obtained from miaolingbio (Wuhan, China). Most of the primary antibodies used in the study are against human, and the similarities between the target proteins from the two species (human and monkey) are high.

### Cell culture and virus propagation

African green monkey kidney epithelial cell line (Vero) was obtained from ATCC. Cells were grown in DMEM medium (JSBio, China) supplemented with 10% fetal bovine serum (FBS), 100 U/mL penicillin and 100 μg/mL streptomycin (BI, ISR) at 37 °C with 5% CO_2_ in a humidified atmosphere. The CDV3-CL vaccine strain was obtained from the Institute of Special Animal and Plant Sciences, Chinese Academy of Agricultural Sciences (Changchun, China). The CDV3-CL strain were passaged in Vero cells. The viral titer was determined by the Reed-Muench method in 96-well plates and expressed as a 50% tissue culture infectious dose (TCID_50_) [68].

### Virus infection and pharmaceutical treatment

Vero cells were not treated or treated with 200 nM, 500 nM, 1000 nM Rapamycin, 5 μM Chloroquine, 200 nM (final concentration) Wortmannin for 4 h, followed by CDV infection (multiplicity of infection, MOI = 1). Cells were covered with DMEM medium containing 2% FBS and certain concentration of the above inducer or inhibitor and cultured at 37 °C with 5% CO2.

### Plasmid construction

CDV N gene was amplified from the CDV3-CL genomic cDNA used in this study and cloned into the p3 × Flag-CMV vectors according to the manufacturer's instructions of the ClonExpress MultiS Cloning Kit (Vazyme, China).

### Plasmid transfection

Vero cells were cultured in Petri dishes until 70% fusion. 2 μg of plasmid was diluted in 200 μL of JetPRIME buffer, then 4 μL of JetPRIME transfection reagent was added, mixed well and incubated at room temperature for 10 min. The mixture was added to each dish and mixed well. If transfection is performed in laser confocal-specific Petri dishes, the plasmids and reagents are halved.

### Transfection and gene silencing with siRNAs

siRNAs targeting the Vero cell ATG5 were designed using the online design tool Block-iTRNAi Designer (Invitrogen, USA). siRNAs were synthesized by GENEWIZ Biotechnology (Suzhou, China). Vero cells were transfected with 100 pmol siRNA using JetPRIME transfection reagent (Ployplus, FR) at a 35 mm dish according to the manufacturer's instructions. Scrambled siRNA was used as a negative control. Silencing efficiency was measured by Western blot analysis. The sequence of the siRNA targeting ATG5 used in this study: GATTCATGGAATTGAGCCA (5’-3’).

### Immunoblotting

Cells were collected and lysed with RIPA strong lysis buffer (Beyotime) containing the protease inhibitor PMSF (Beyotime, China), and 5 × loading buffer (BOSTER, China) was added, and the mixture was sonicated on ice for 15 s. Denaturation was performed at 100 °C for 10 min. Proteins were separated on an electrophoretic sodium dodecyl sulfate–polyacrylamide gel and then transferred to polyvinylidene difluoride (PVDF) membranes (Millipore, USA). The membranes were blocked for 1 h or more with 5% skim milk (BBI, China) and cut according to the size of the target protein before hybridization with the antibody. The membranes were incubated with primary antibody overnight at 4 °C, then incubated with peroxidase-coupled secondary antibodies. Protein bands were detected using chemiluminescence image analysis system (Amersham Imager 600, GE, USA), with an ECL kit (Advansta, USA). The intensity of the target protein blots was analyzed using ImageJ software (NIH, USA). The relative protein level was normalized to β-tubulin. The dilution ratios of antibodies were shown in Table 1.

**Table 1:** The dilution of all the antibodies used for the immunoblotting

Antibodies	Dilution
LC3B	1∶2000
p62	1∶1000
p-mTOR	1∶1000
mTOR	1∶2000
p-AKT	1∶1000
AKT	1∶1000
ATG5	1∶2000
CDV-N	1∶5000
β-Tubulin	1∶5000
goat anti-mouse IgG-HRP	1∶8000
goat anti-rabbit IgG-HRP	1∶8000

### Immunofluorescence and confocal microscopy

Cells were grown in glass-bottom culture dishes (NEST, China). The CDV-infected at 1 MOI or plasmids-transfected or pharmaceutical-treated cells fixed with 4% paraformaldehyde (Beyotime, China) for 15 min. and then treated with 0.2% Triton X-100 (Solarbio, China) for 15 min and blocked with 5% bovine serum albumin (MP, USA) for 1 h. Cells are then incubated with primary antibody overnight at 4 °C or 2 h at 37 °C, followed by incubation with the appropriate fluorescent dye-conjugated secondary antibody for 1 h at 37 °C under dark conditions. Cells were then stained with DAPI (Beyotime, China) for 5 min. Samples were then imaged using a confocal fluorescence microscope (Objective magnification, × 60) (Leica SP8, Germany). The dilution ratios of antibodies were shown in Table 2.

**Table 2:** The dilution of all the antibodies used for immunofluorescence

Antibodies	Dilution
LC3B	1∶300
CDV-N	1∶500
LAMP1	1∶300
Alexa Fluor 488 goat anti-rabbit IgG	1∶500
Alexa Fluor 594 goat anti-mouse IgG	1∶500
Alexa Fluor 594 goat anti-rabbit IgG	1∶300

### Quantitative real-time PCR

Vero cells were treated with pharmaceuticals or siRNA, and infected with CDV (MOI = 1). Total RNA was extracted using the Viral RNA/DNA Extraction Kit (TaKaRa, China) according to the manufacturer's protocol. cDNA was synthesized by reverse transcription using PrimeScript RT Master Mix reagent (TaKaRa, China). Luna Universal qPCR Master Mix (New England Biolabs, USA) was used to perform quantitative real-time PCR. For CDV-specific detection, primer pairs targeting the region corresponding to the N gene were used, and a recombinant plasmid containing the CDV N gene was used to construct a standard curve for calculating the viral RNA copy number in different samples. qPCR was performed with the primers shown below:

- CDV N Forward: 5′-GAGAATTAACAACTATTGAATC-3′
- CDV N Reverse: 5′-CATAGCATAACTCCAGAG-3′

### Statistical analysis

The Data are expressed as mean ± standard deviation (SD). The significance of the variability between treatment groups was analyzed by two-way analysis of variance (ANOVA) test by GraphPad Prism software (version 8.3, USA). Differences were considered statistically significant at *P* < 0.05.

## Supplementary Information

**Additional file 1:** **Figure S1.** Fluorescence intensity and co-localization analysis of Cherry and GFP. **Figure S2.** N protein significantly increased the level of LC3-II.

**Additional file 2.** The original blots for the figures.

## Data Availability

All data analysed during this study are included in this published article. The raw data generated during the current study are available from the corresponding author on reasonable request.

## Abbreviations

CDV: Canine distemper virus
CQ: Chloroquine
FBS: Fetal bovine serum
hpi: Hours post-infection
LC3B: Microtubule-associated protein 1 light chain 3β
PBS: Phosphate buffered saline
TCID_50_: The median tissue culture infective dose
WM: Wortmannin
