# Supplementary material for: Canine distemper virus N protein induces autophagy to facilitate viral replication
Source: BMC Vet Res. 2023 Mar 15;19:60. doi: 10.1186/s12917-023-03575-7 (PMC10015816; doi:10.1186/s12917-023-03575-7)
Supplement: Supplementary file 1 — Additional file 1: Figure S1. Fluorescence intensity and co-localization analysis of Cherry and GFP. Figure S2. N protein significantly increased the level of LC3-II. [file 12917_2023_3575_MOESM1_ESM.docx]

**Supplementary materials**


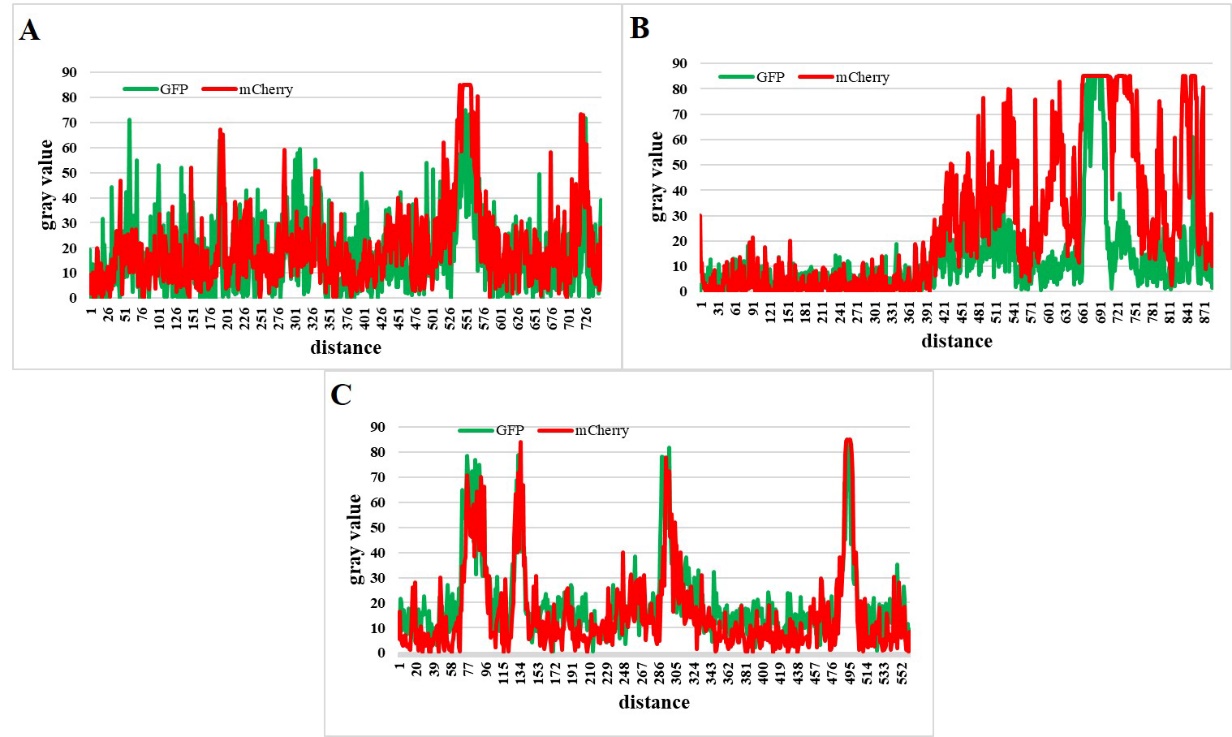


**Fig.S1** Fluorescence intensity and co-localization analysis of Cherry and GFP. Vero cells were transfected with pmCherry-GFP-LC3B for 24 h, followed by CDV infection (MOI = 1) and treatment with E64d. The fluorescence signals were visualized by confocal immunofluorescence microscopy. (A) Image J software analysis of fluorescence intensity and co-location of GFP and mCherry at 24 hpi. (B) Image J software analysis of fluorescence intensity and co-location of GFP and mCherry at 32 hpi. (C) Image J software analysis of fluorescence intensity and co-location of GFP and mCherry after E64d treatment.


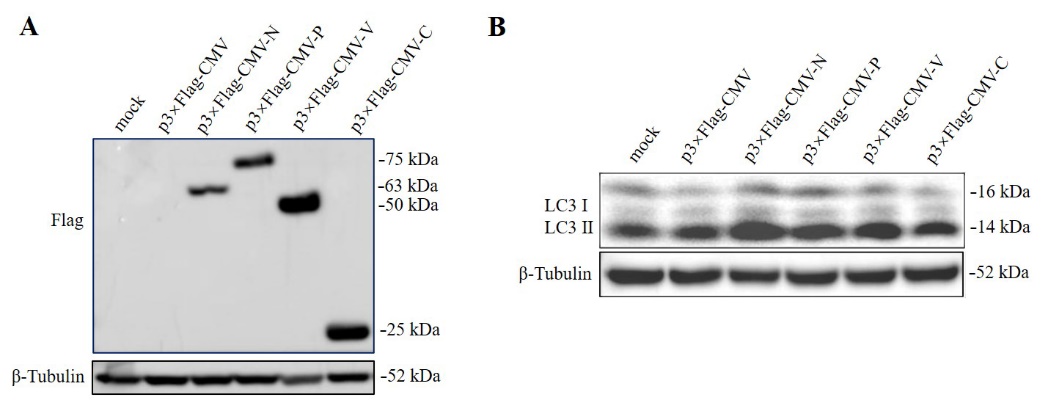


**Fig.S2** N protein significantly increased the level of LC3-II. Vero cells were transfected with empty vectors or various plasmids expressing Flag-tagged N, P, V, and C proteins for 48 h. (A) The cell samples were analyzed by immunoblotting with an anti-Flag antibody. (B) The cell samples were analyzed by immunoblotting with anti-LC3 and anti-β-tubulin (loading control) antibodies.
