# Supplementary material for: Canine distemper virus N protein induces autophagy to facilitate viral replication
Source: BMC Vet Res. 2023 Mar 15;19:60. doi: 10.1186/s12917-023-03575-7 (PMC10015816; doi:10.1186/s12917-023-03575-7)

**The original blots for the figures**

**Note:** For our gel data, the target proteins analyzed by western blot were transferred from SDS-PAGE and the PVDF membrane would be sliced into different strips according to their protein size identified with the loading marker before hybridization with the antibody. Then the panel of antibodies targeting these proteins were used for western blot analysis. Our SOP for processing the gel data only collected the pictures with target protein regions instead of the whole gel, so here are the original gels for the data we presented in the manuscript (grayscale values below the blots).

**Fig. 1B~LC3**

| hpi | 2 | 4 | 8 | 12 |
| --- | --- | --- | --- | --- |


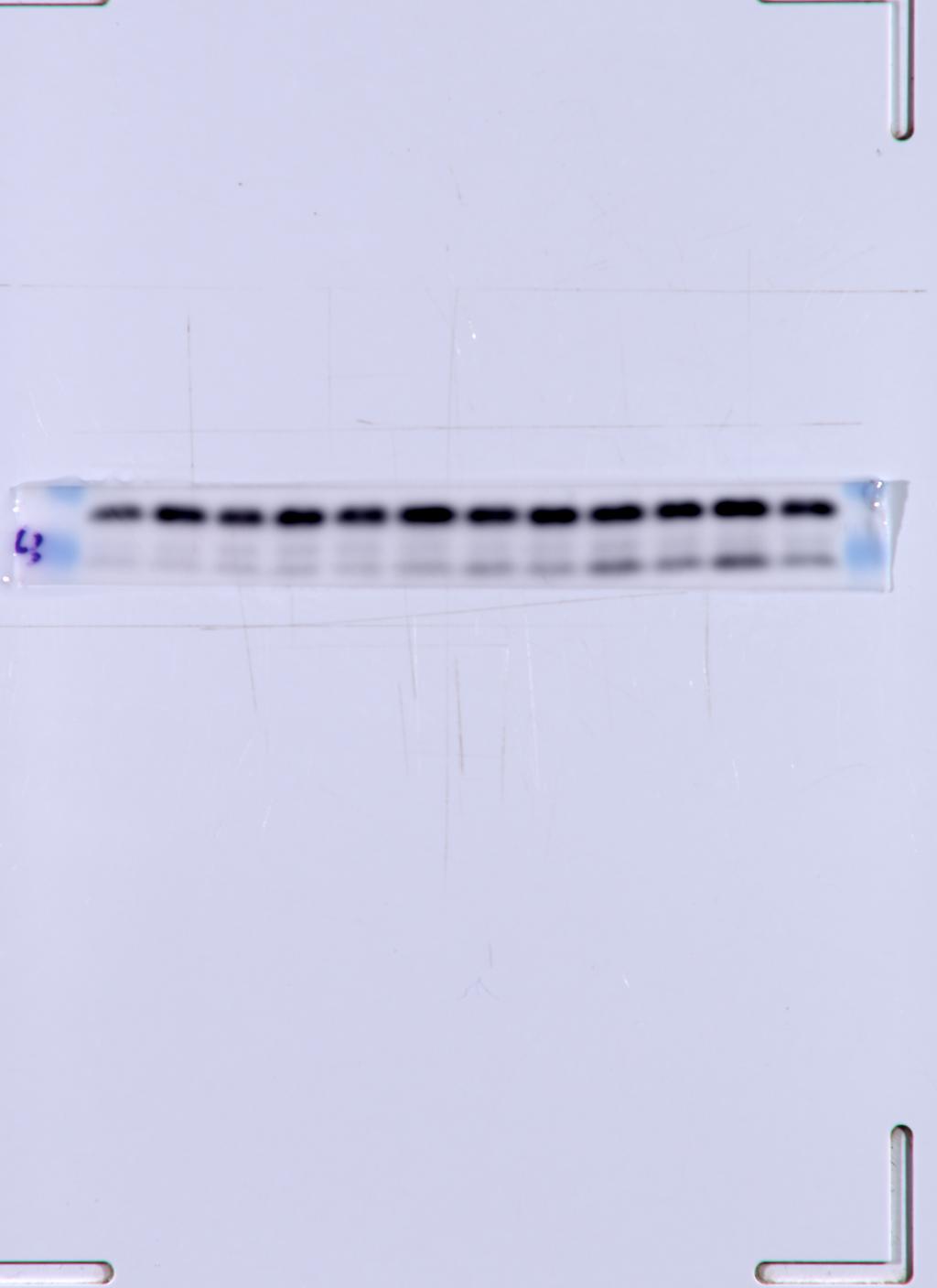


**Fig. 1B~β-tubulin**

| hpi | 2 | 4 | 8 | 12 |
| --- | --- | --- | --- | --- |


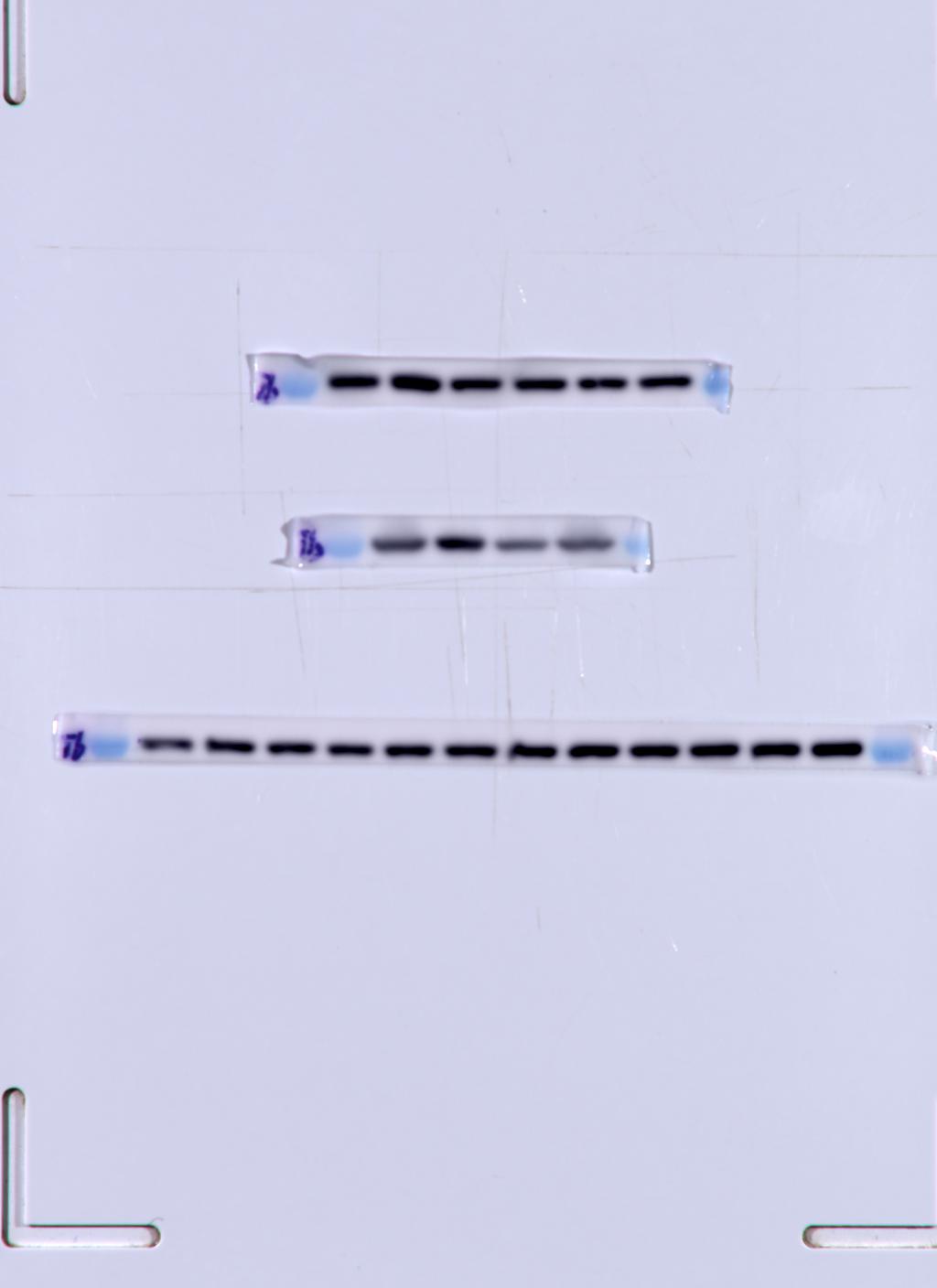


**Fig. 1C~LC3**

| hpi | 16 | 20 | 24 | 28 | 32 | 36 |
| --- | --- | --- | --- | --- | --- | --- |

**
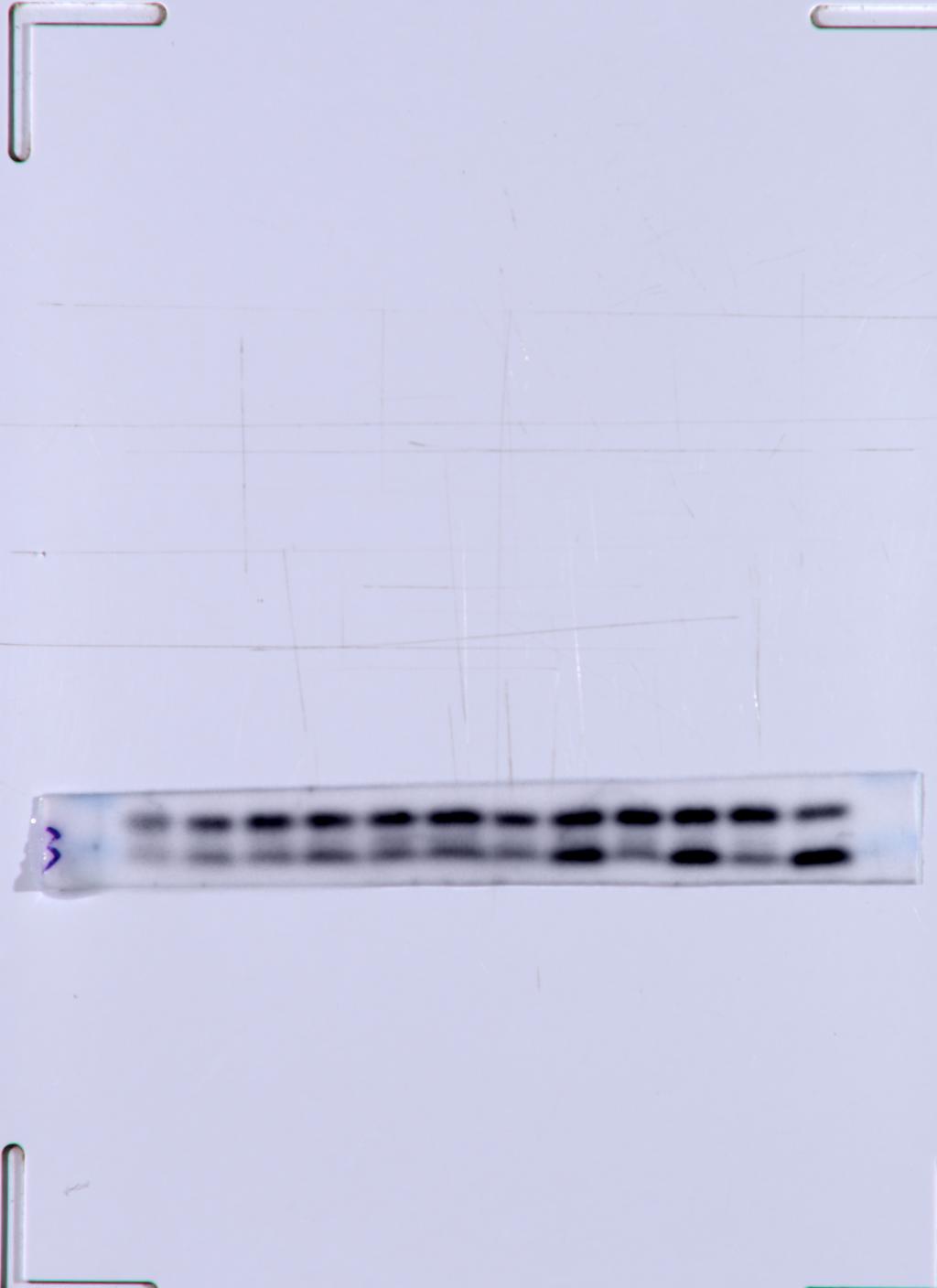
**

| Gray scale | Mock  CDV | 0.642  1.042 | 0.944  1.165 | 0.672  1.973 | 1.051  2.106 | 1.197  2.837 |
| --- | --- | --- | --- | --- | --- | --- |

**Fig. 1C~CDV-N**

| hpi | 16 | 20 | 24 | 28 | 32 | 36 |
| --- | --- | --- | --- | --- | --- | --- |

**
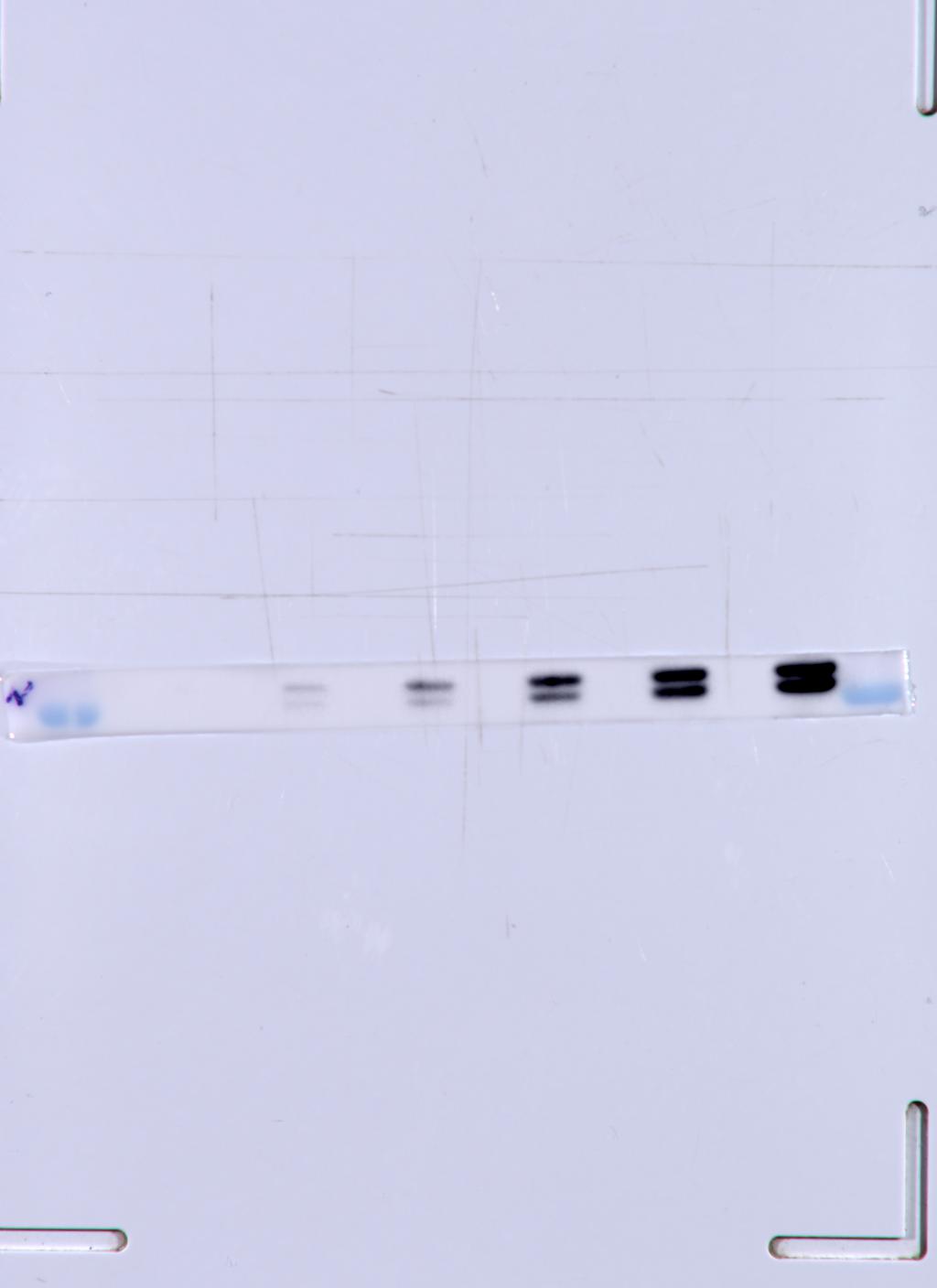
**

| Gray scale | CDV | 0.230 | 0.720 | 2.618 | 4.268 | 5.582 |
| --- | --- | --- | --- | --- | --- | --- |

**Fig. 1C~β-tubulin**

| hpi | 16 | 20 | 24 | 28 | 32 | 36 |
| --- | --- | --- | --- | --- | --- | --- |


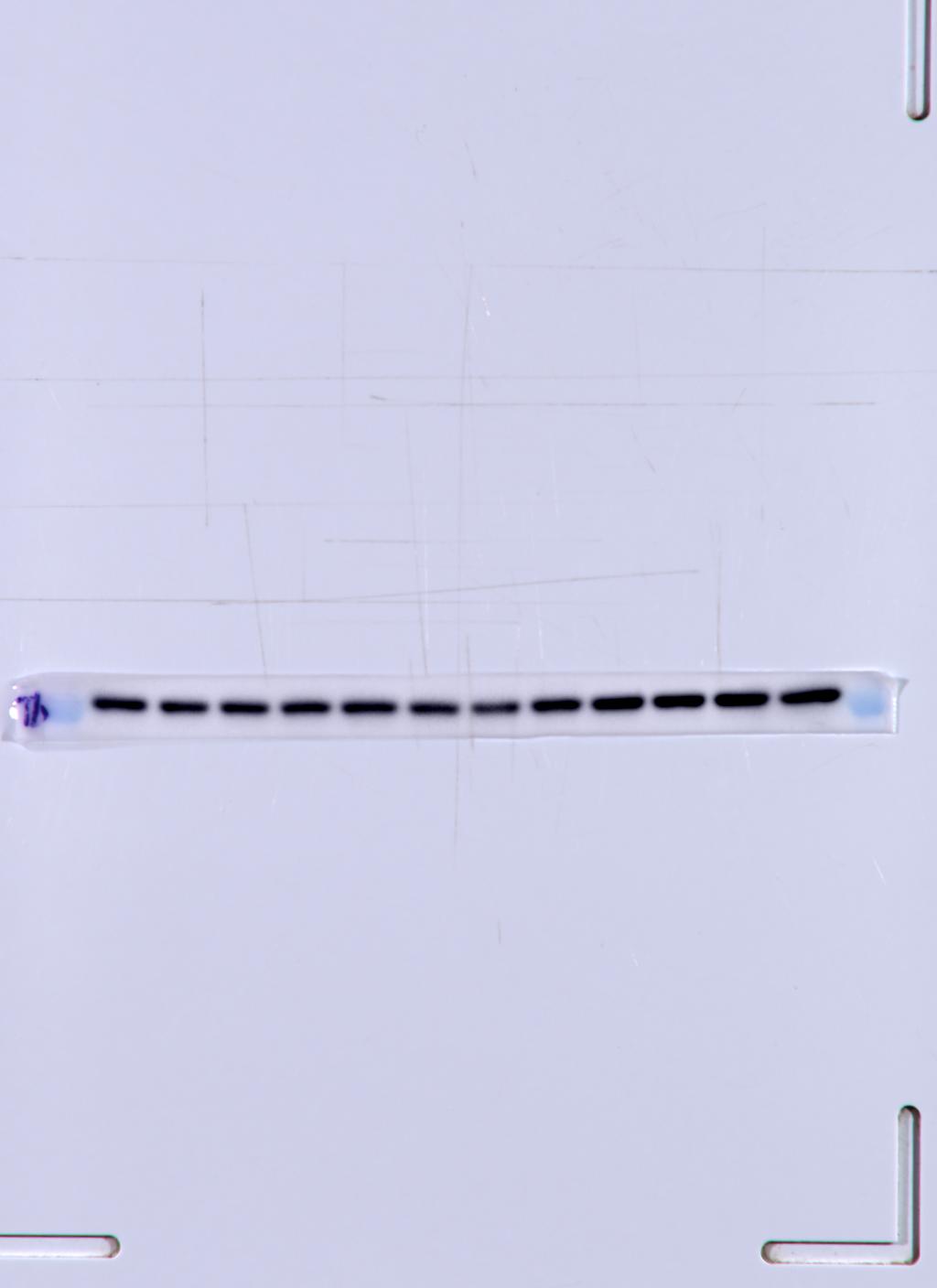


| Gray scale | Mock  CDV | 1.569  1.611 | 1.922  1.558 | 1.446  1.822 | 2.053  1.948 | 2.236  2.264 |
| --- | --- | --- | --- | --- | --- | --- |

**Fig. 2A~p62**

| hpi | 24 | 28 | 32 | 36 | 40 |
| --- | --- | --- | --- | --- | --- |


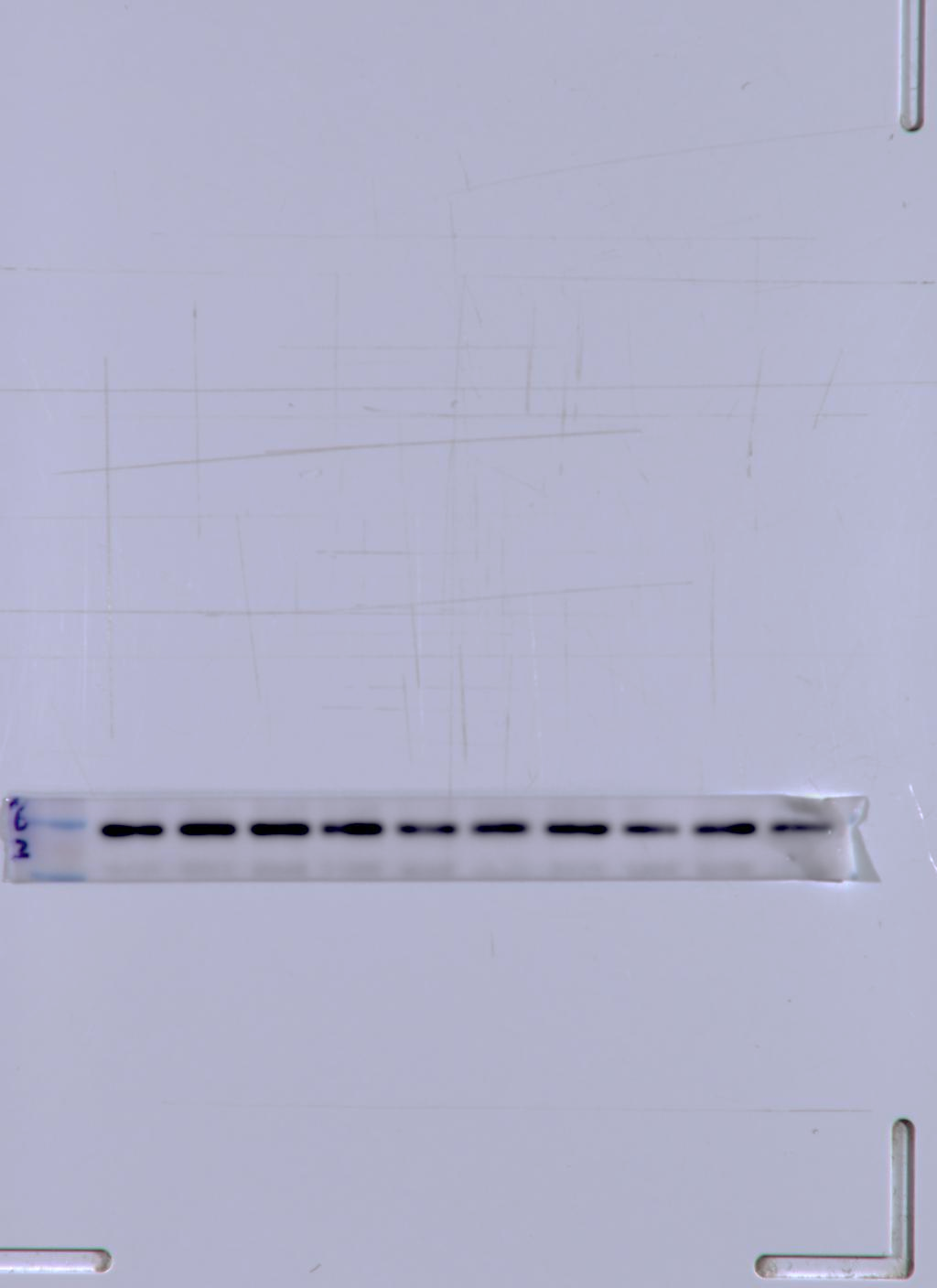


| Gray scale | 2.332  2.206 | 2.280  2.202 | 1.665  1.784 | 2.114  1.332 | 1.999  1.475 | Mock  CDV |
| --- | --- | --- | --- | --- | --- | --- |

**Fig. 2A~CDV-N**

| hpi | 24 | 28 | 32 | 36 | 40 |
| --- | --- | --- | --- | --- | --- |


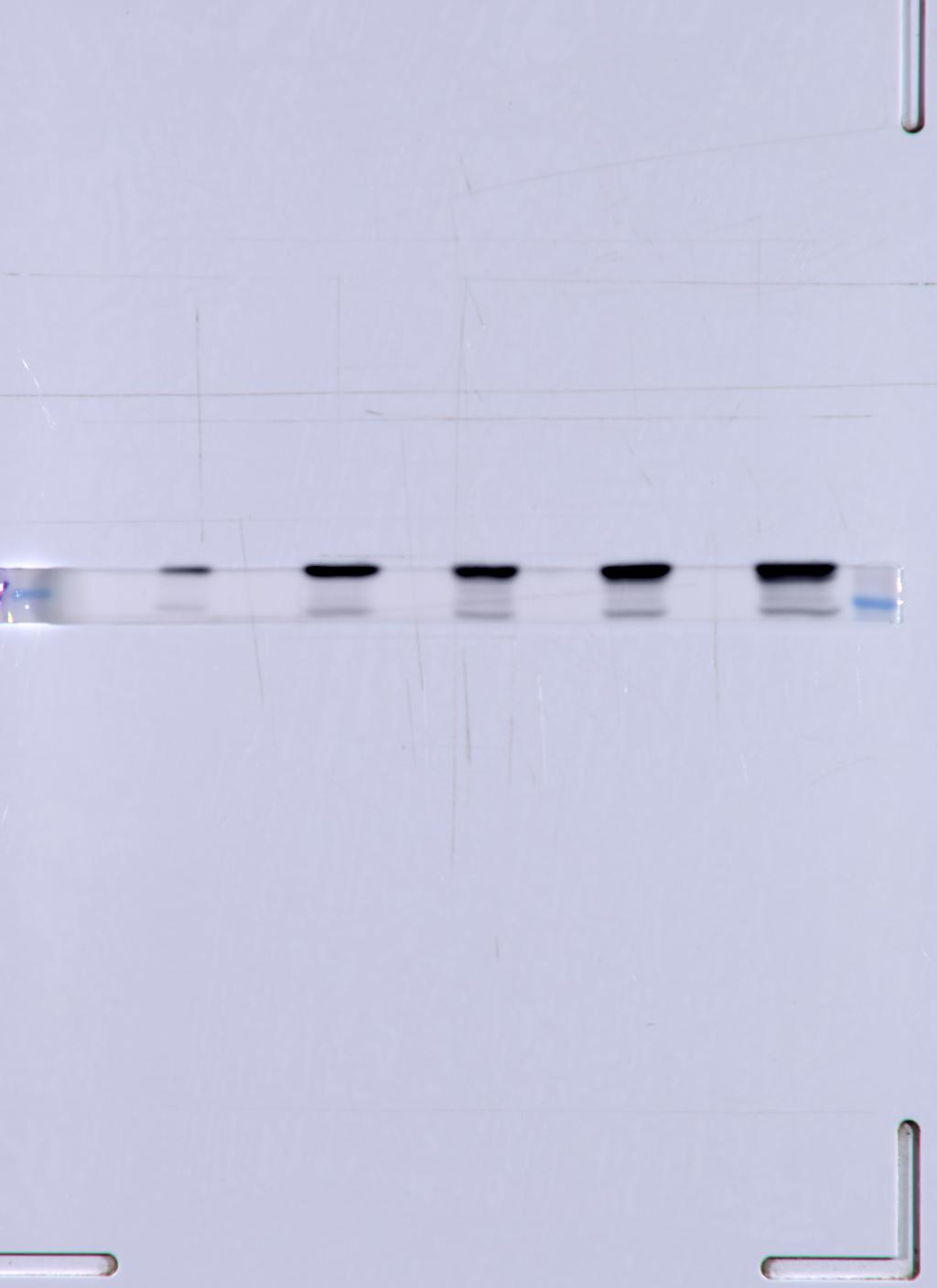


**Fig. 2A~β-tubulin**

| hpi | 24 | 28 | 32 | 36 | 40 |
| --- | --- | --- | --- | --- | --- |


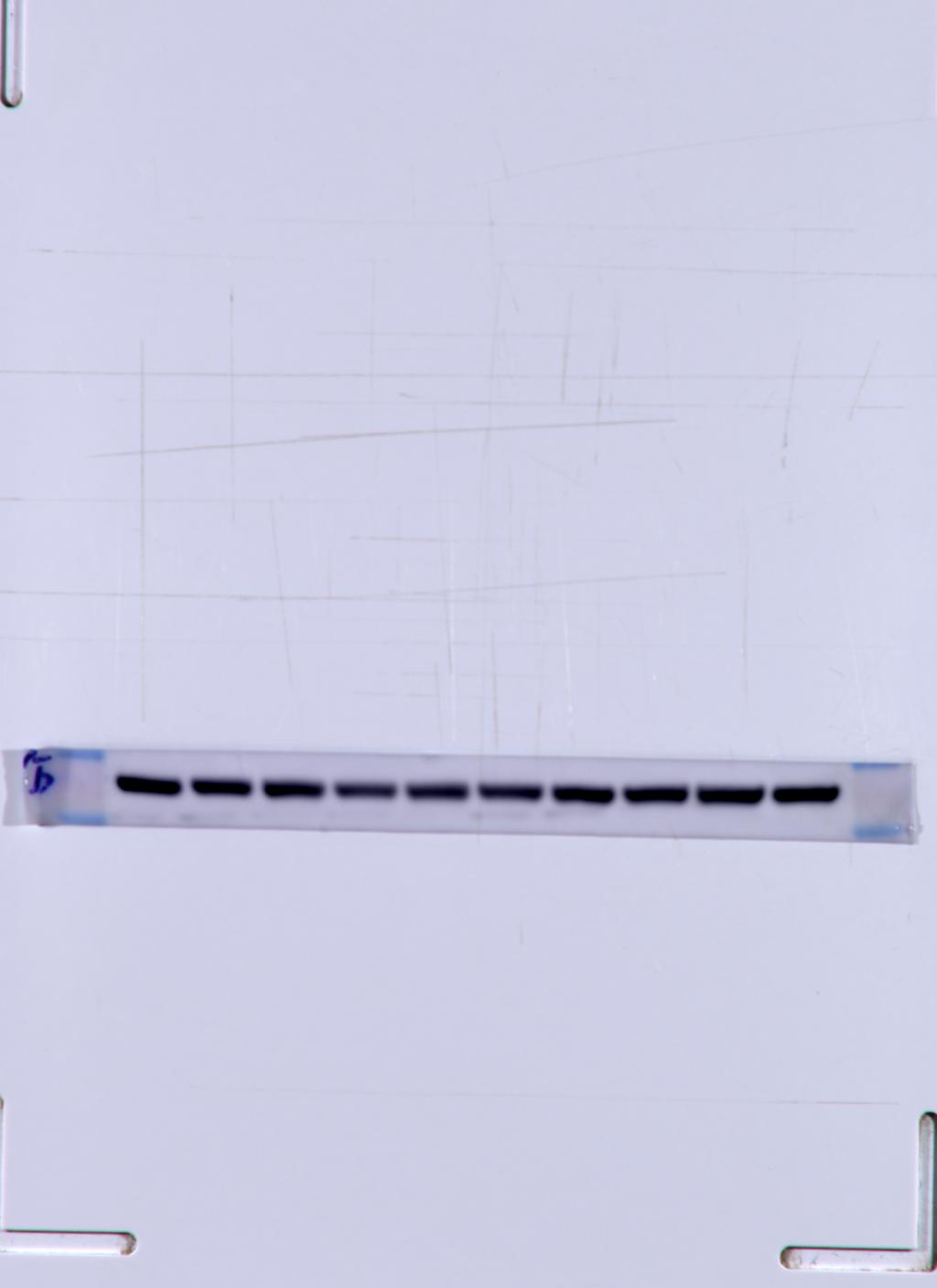


| Gray scale | 2.581  2.198 | 2.245  1.693 | 2.008  2.031 | 2.490  2.531 | 2.742  2.603 | Mock  CDV |
| --- | --- | --- | --- | --- | --- | --- |

**Fig. 2C~CDV-N**

| hpi | 24 | | | 28 | | |
| --- | --- | --- | --- | --- | --- | --- |
|  | mock | rapamycin | CDV | mock | rapamycin | CDV |


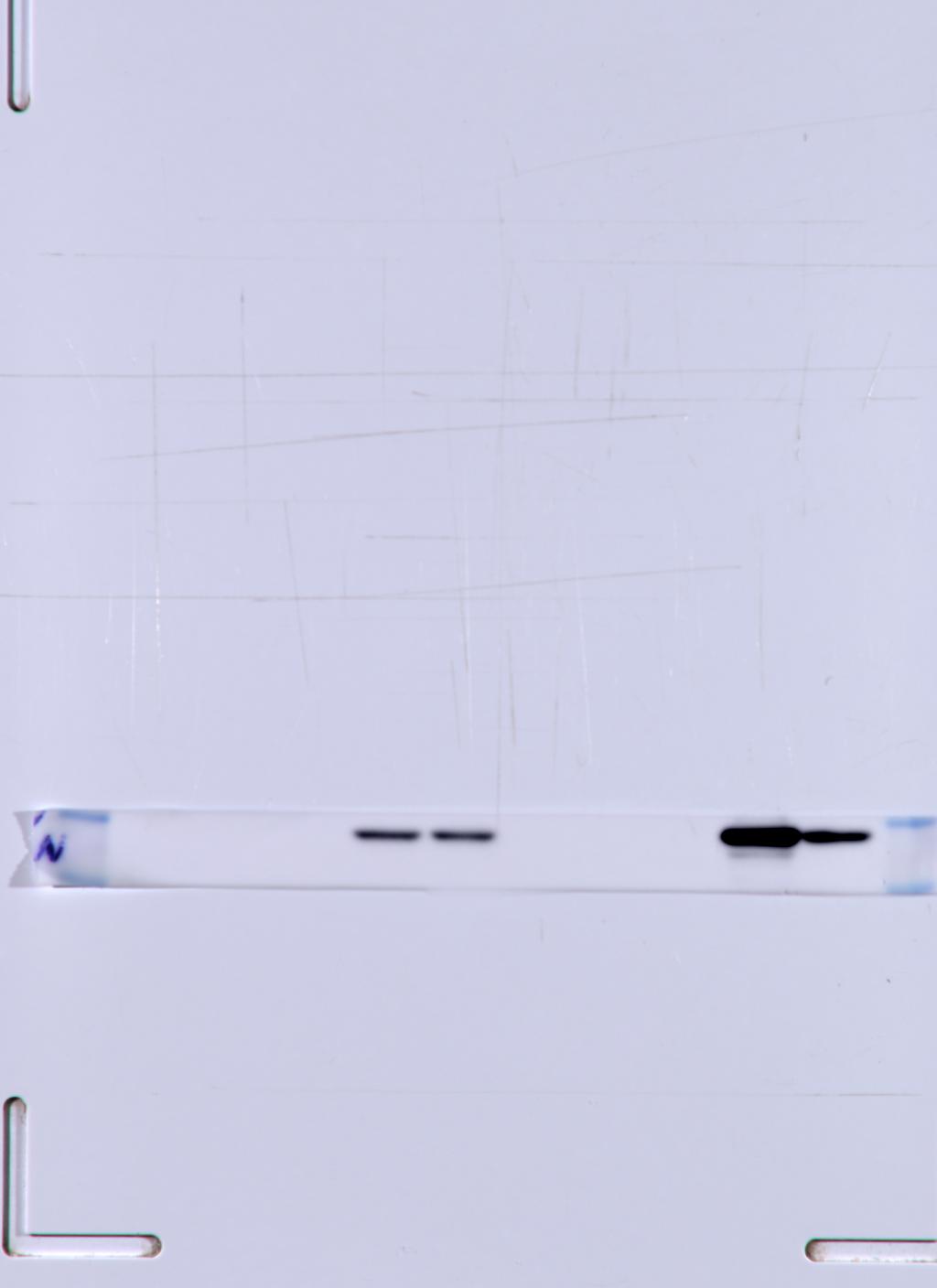


| Gray scale |  |  | 1.337  1.154 |  |  | 4.681  2.256 | E64d-  E64d+ |
| --- | --- | --- | --- | --- | --- | --- | --- |

**Fig. 2C~LC3**

| hpi | 24 | | | 28 | | |
| --- | --- | --- | --- | --- | --- | --- |
|  | mock | rapamycin | CDV | mock | rapamycin | CDV |


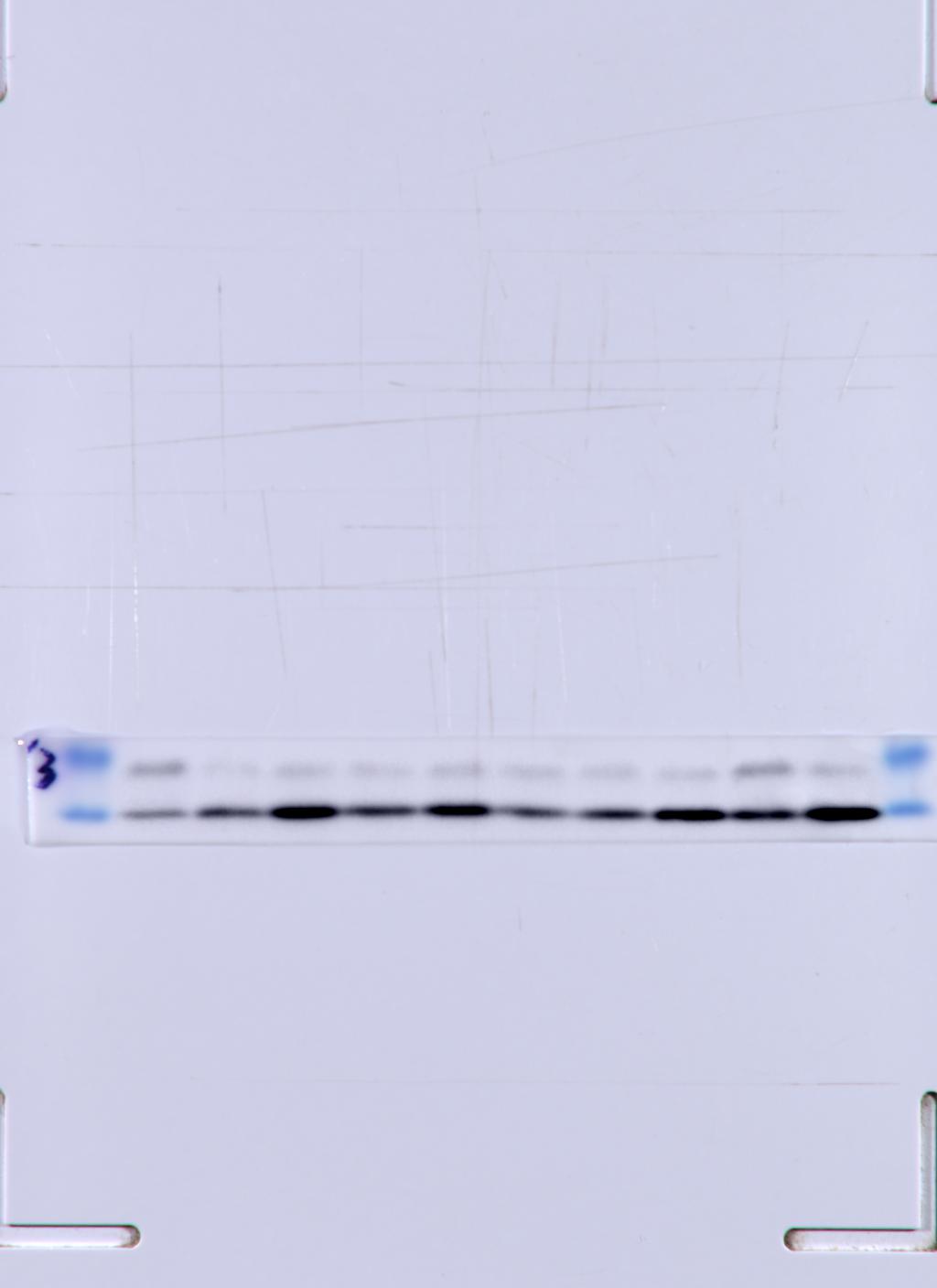


| Gray scale | 0.647 | 1.292  2.593 | 1.657  2.273 | 1.194 | 1.470  2.478 | 1.919  3.096 | E64d-  E64d+ |
| --- | --- | --- | --- | --- | --- | --- | --- |

**Fig. 2C~p62**

| hpi | 24 | | | 28 | | |
| --- | --- | --- | --- | --- | --- | --- |
|  | mock | rapamycin | CDV | mock | rapamycin | CDV |


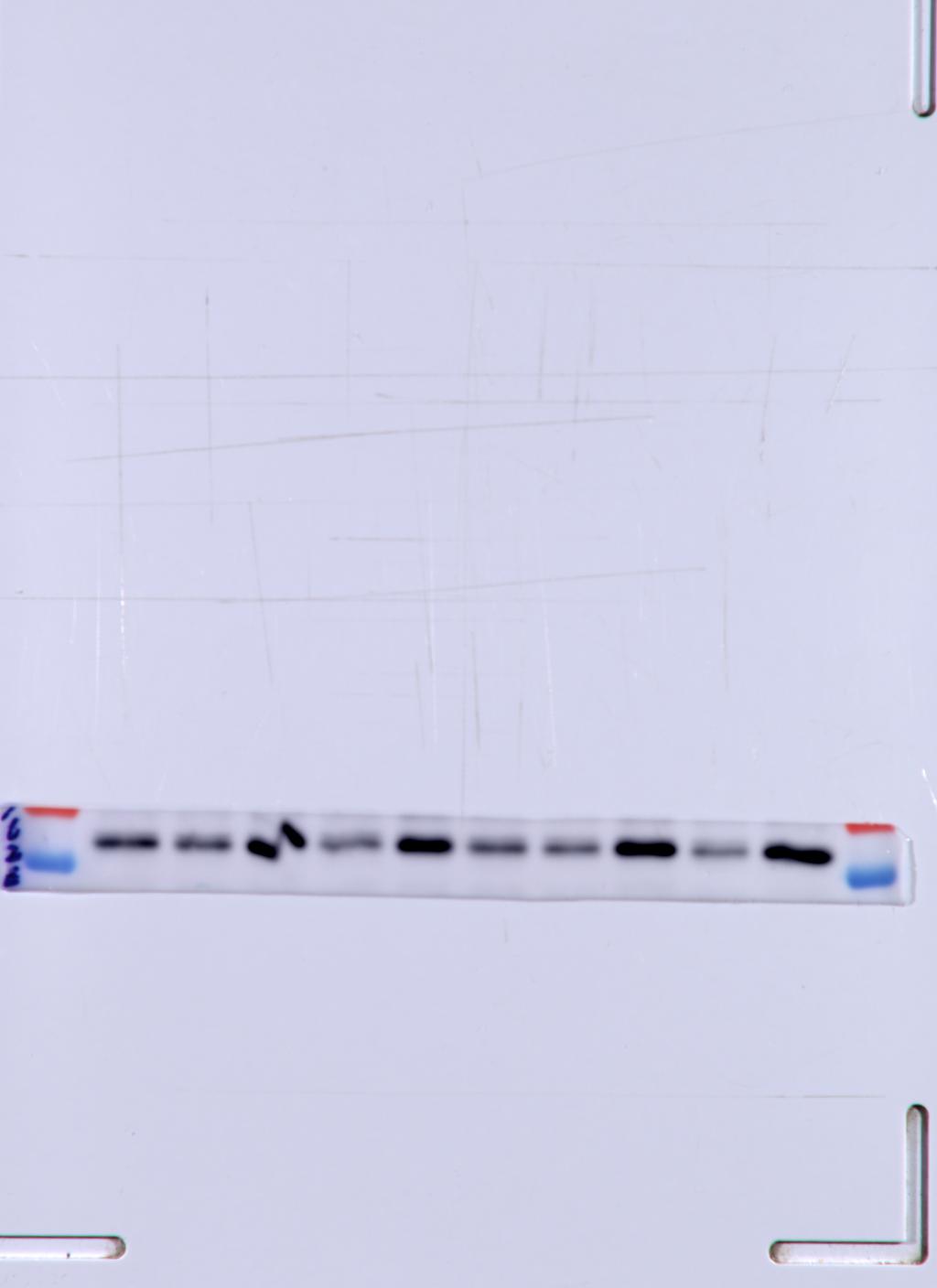


| Gray scale | 1.717 | 1.179  2.544 | 1.063  2.316 | 1.209 | 0.922  2.674 | 0.805  2.948 | E64d-  E64d+ |
| --- | --- | --- | --- | --- | --- | --- | --- |

**Fig. 2C~β-tubulin**

| hpi | 24 | | | 28 | | |
| --- | --- | --- | --- | --- | --- | --- |
|  | mock | rapamycin | CDV | mock | rapamycin | CDV |


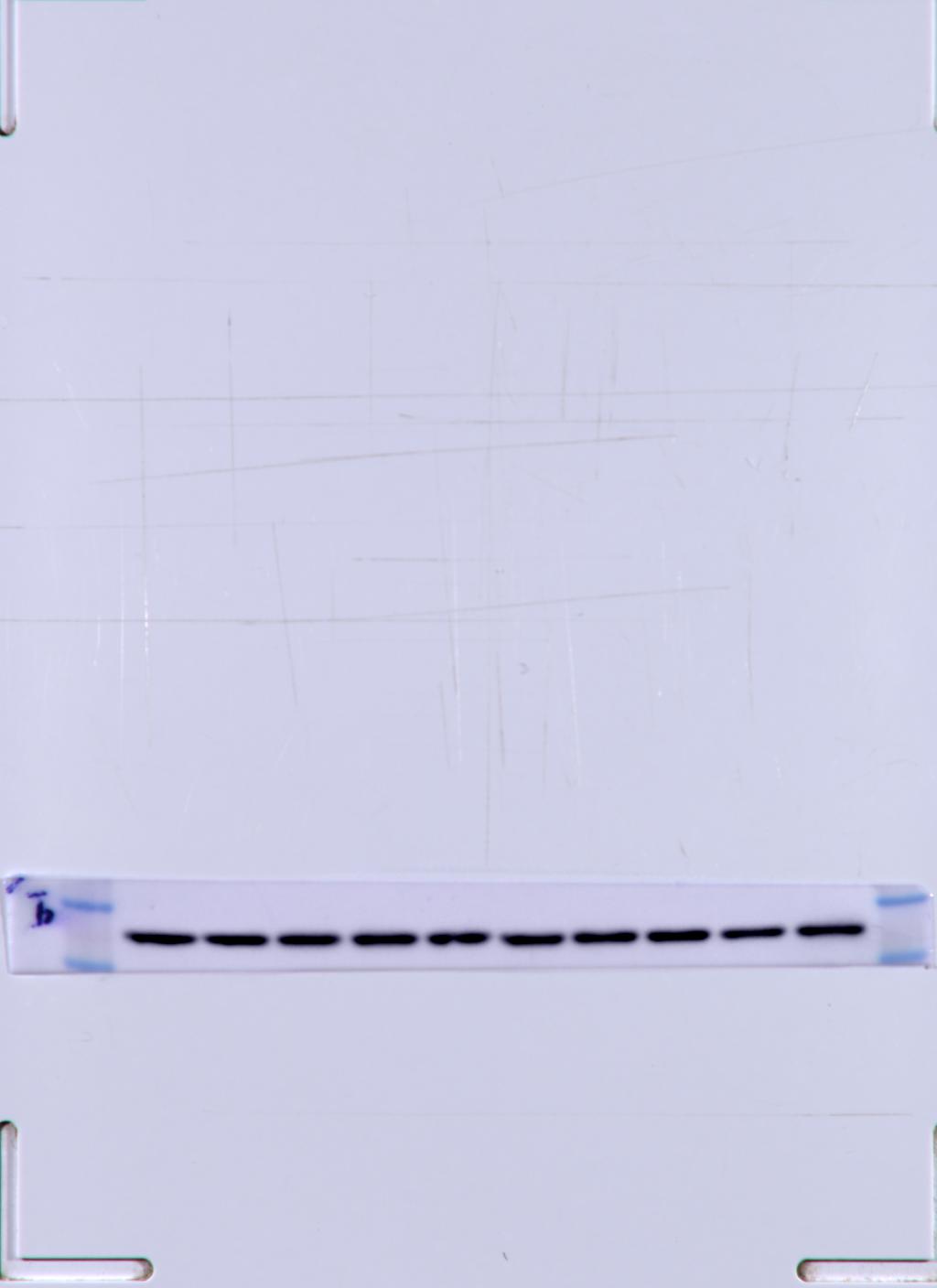


| Gray scale | 2.078 | 1.822  1.835 | 2.006  1.841 | 1.853 | 1.890  1.878 | 1.636  1.955 | E64d-  E64d+ |
| --- | --- | --- | --- | --- | --- | --- | --- |

**Fig. 3A~CDV-N**

| hpi | 26 | | 30 | | | |
| --- | --- | --- | --- | --- | --- | --- |
|  | - | 200 | - | 200 | 500 | 1000 |


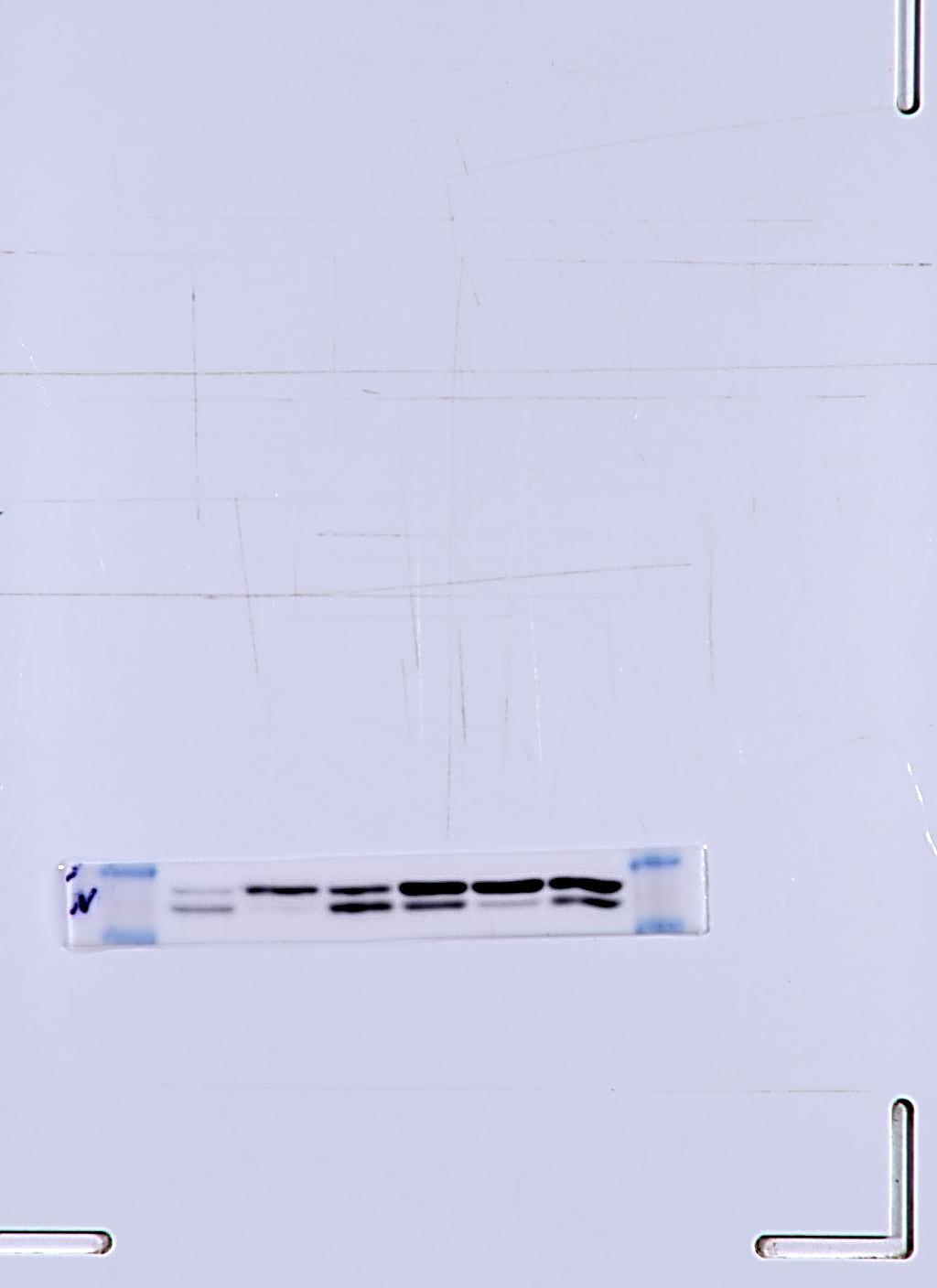


| Gray scale | 0.157 | 1.310 | 1.177 | 2.623 | 2.594 | 2.638 |
| --- | --- | --- | --- | --- | --- | --- |

**Fig. 3A~LC3**

| hpi | 26 | | 30 | | | |
| --- | --- | --- | --- | --- | --- | --- |
|  | - | 200 | - | 200 | 500 | 1000 |


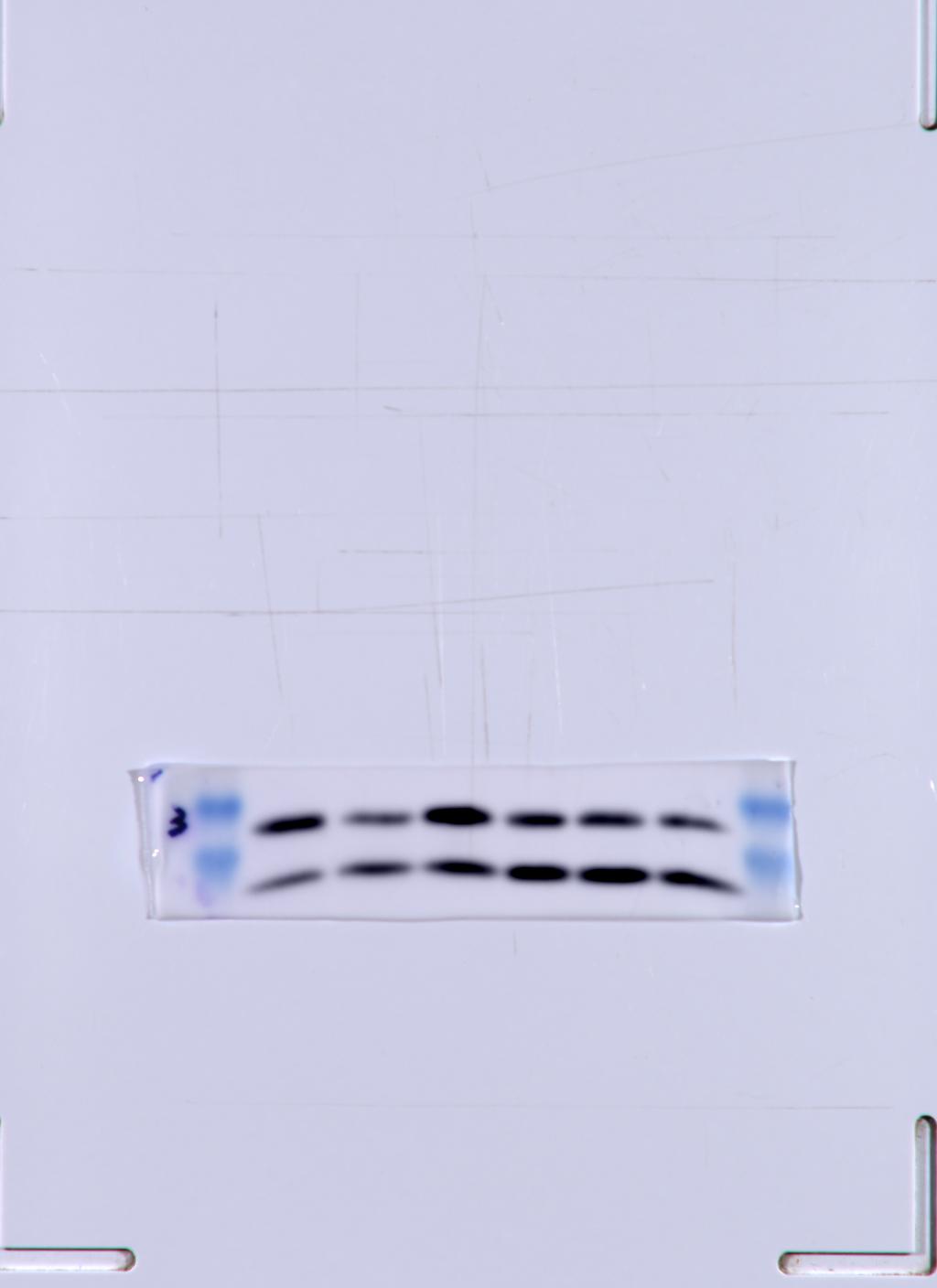


| Gray scale | 1.236 | 1.806 | 2.383 | 2.630 | 2.996 | 2.450 |
| --- | --- | --- | --- | --- | --- | --- |

**Fig. 3A~p62**

| hpi | 26 | | 30 | | | |
| --- | --- | --- | --- | --- | --- | --- |
|  | - | 200 | - | 200 | 500 | 1000 |

**
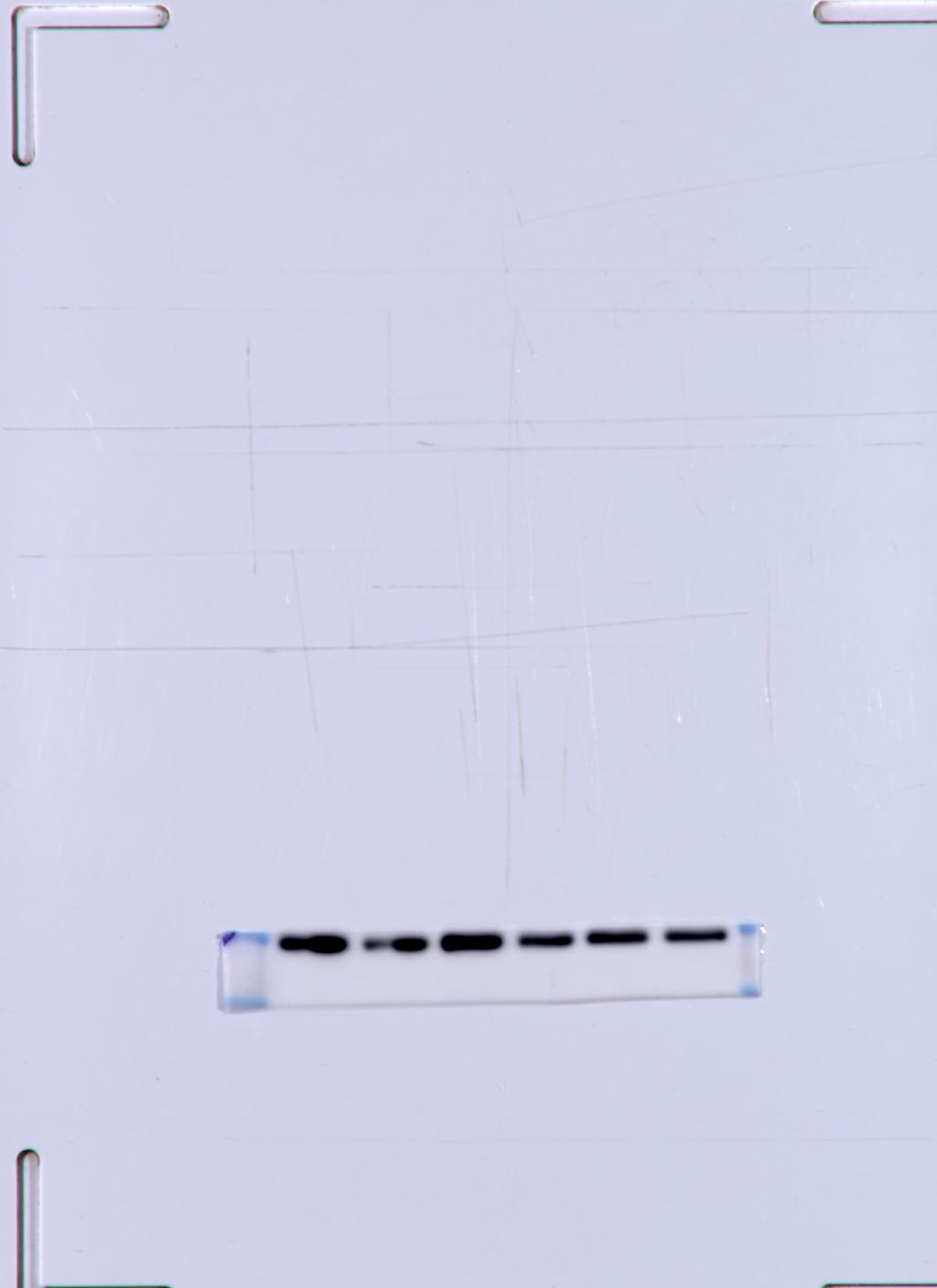
**

| Gray scale | 3.105 | 2.031 | 2.933 | 1.776 | 2.053 | 1.820 |
| --- | --- | --- | --- | --- | --- | --- |

**Fig. 3A~β-tubulin**

| hpi | 26 | | 30 | | | |
| --- | --- | --- | --- | --- | --- | --- |
|  | - | 200 | - | 200 | 500 | 1000 |

**
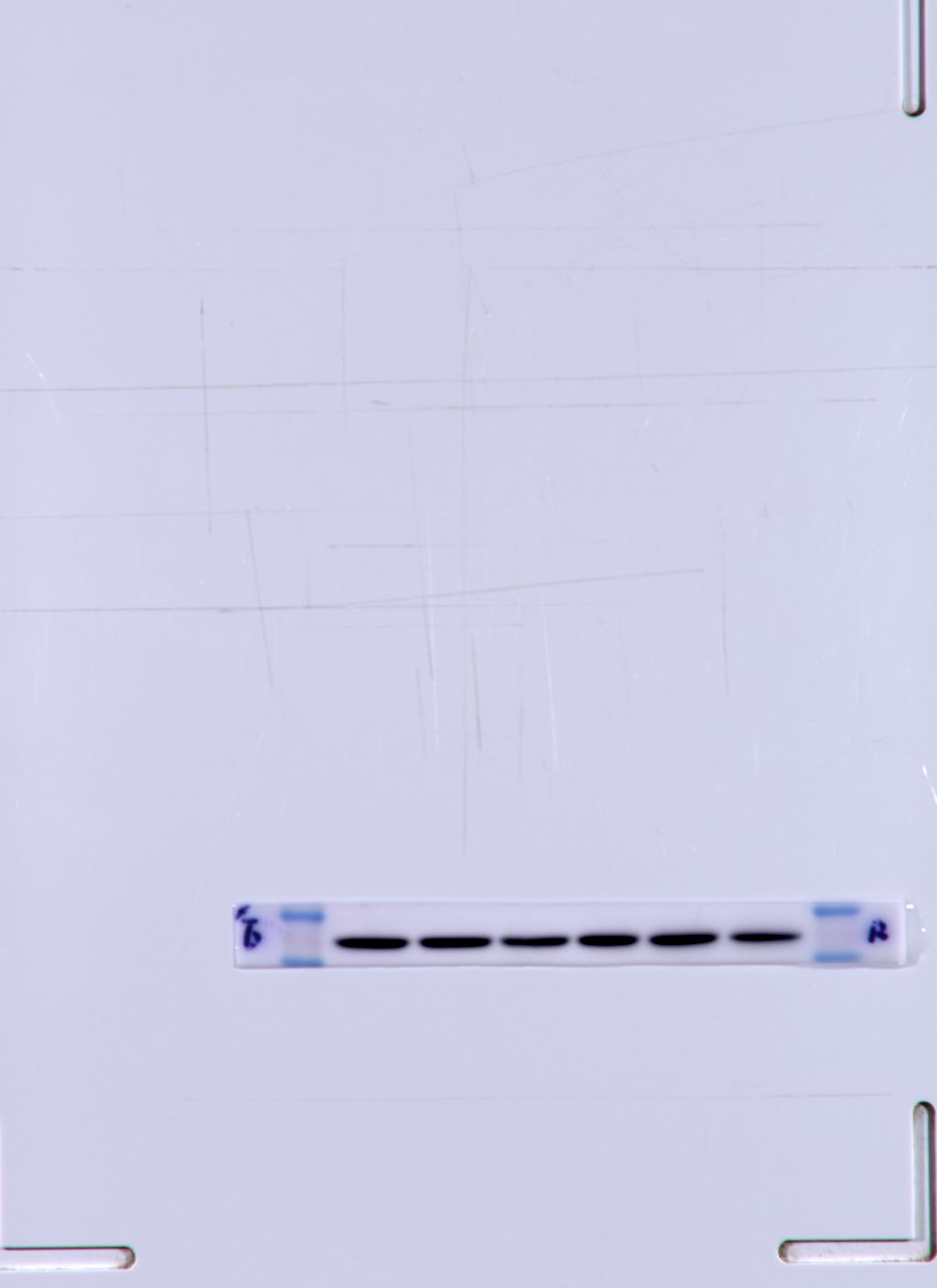
**

| Gray scale | 2.507 | 2.449 | 2.007 | 2.155 | 2.623 | 2.160 |
| --- | --- | --- | --- | --- | --- | --- |

**Fig. 4A~CDV-N**

| hpi | 26 | 30 |
| --- | --- | --- |

**
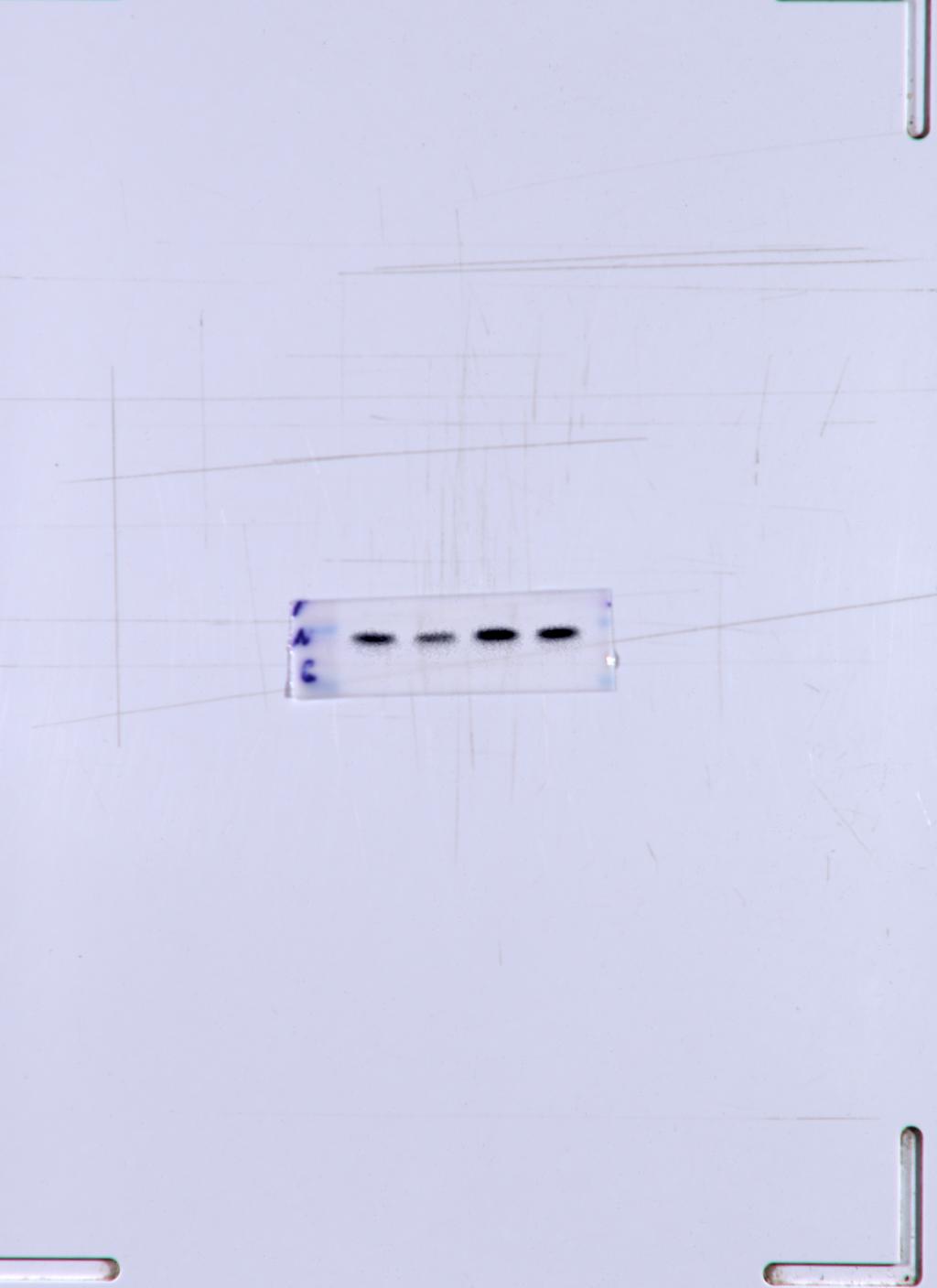
**

| Gray scale | 1.151 | 0.822 | 1.653 | 1.449 |
| --- | --- | --- | --- | --- |

**Fig. 4A~p62**

| hpi | 26 | 30 |
| --- | --- | --- |

**
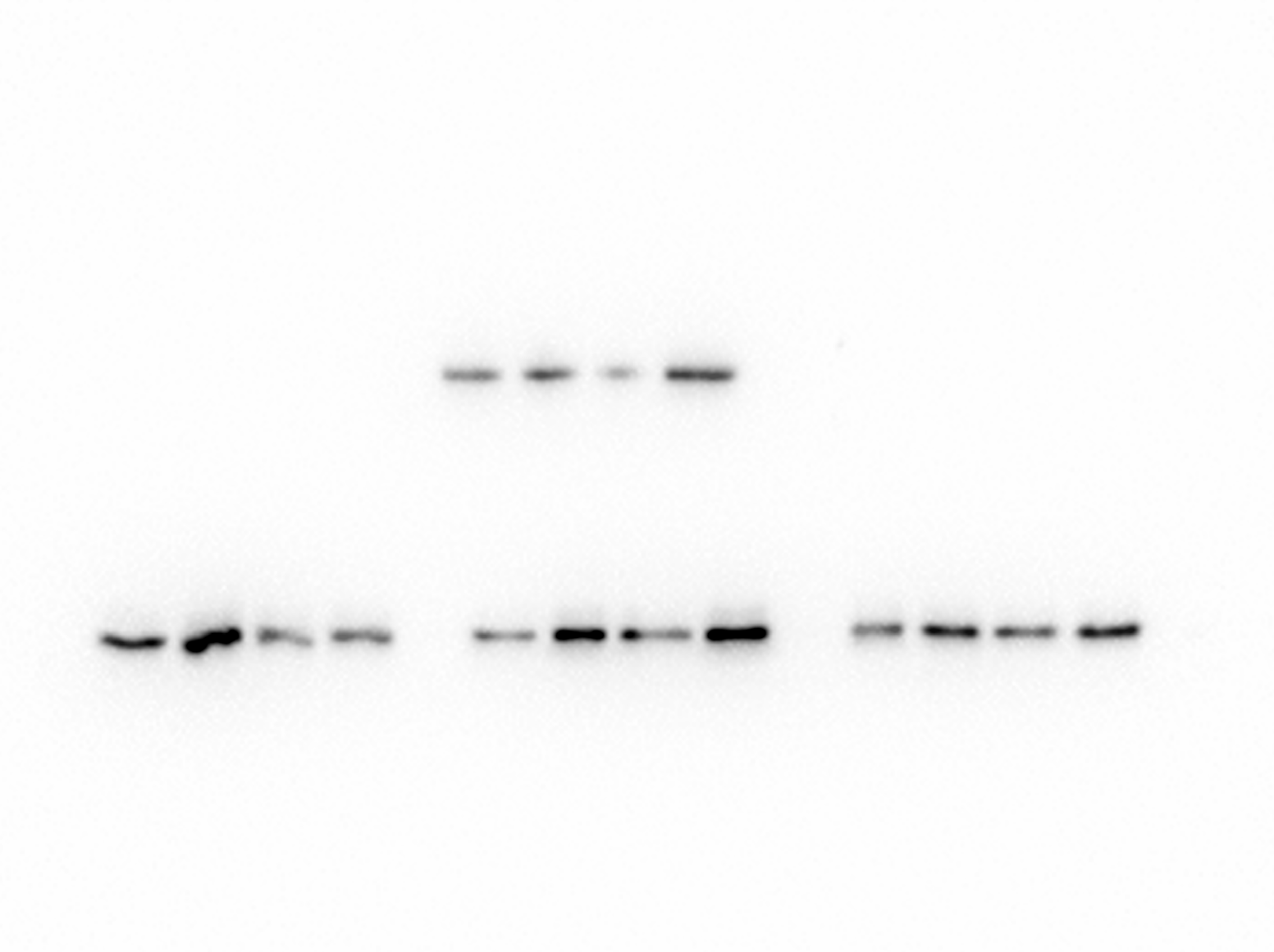
**

| Gray scale | 0.666 | 1.519 | 1.174 | 2.255 |
| --- | --- | --- | --- | --- |

**Fig. 4A~LC3**

| hpi | 26 | 30 |
| --- | --- | --- |

**
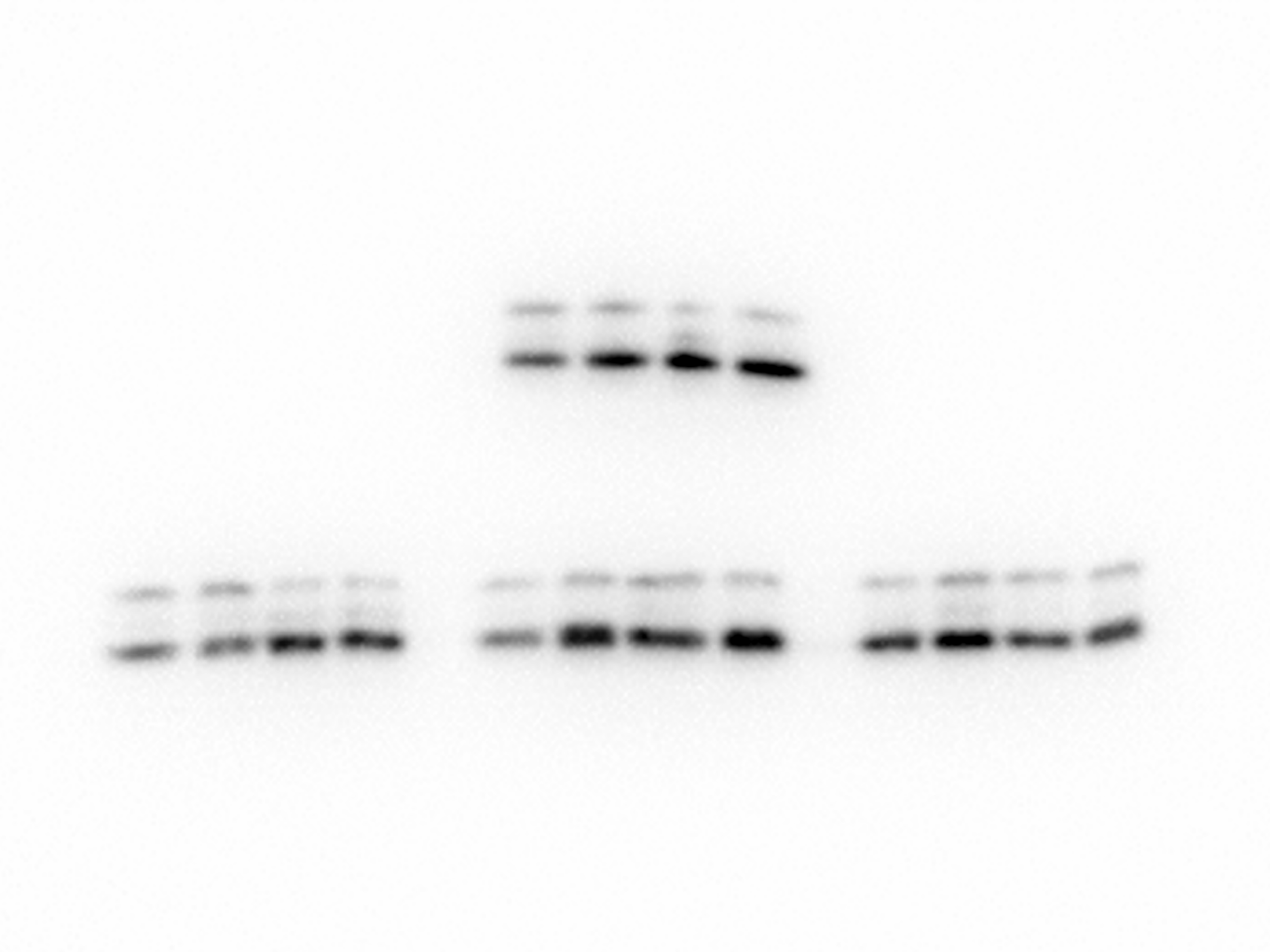
**

| Gray scale | 0.853 | 2.011 | 2.089 | 2.434 |
| --- | --- | --- | --- | --- |

**Fig. 4A~β-tubulin**

| hpi | 26 | 30 |
| --- | --- | --- |

**
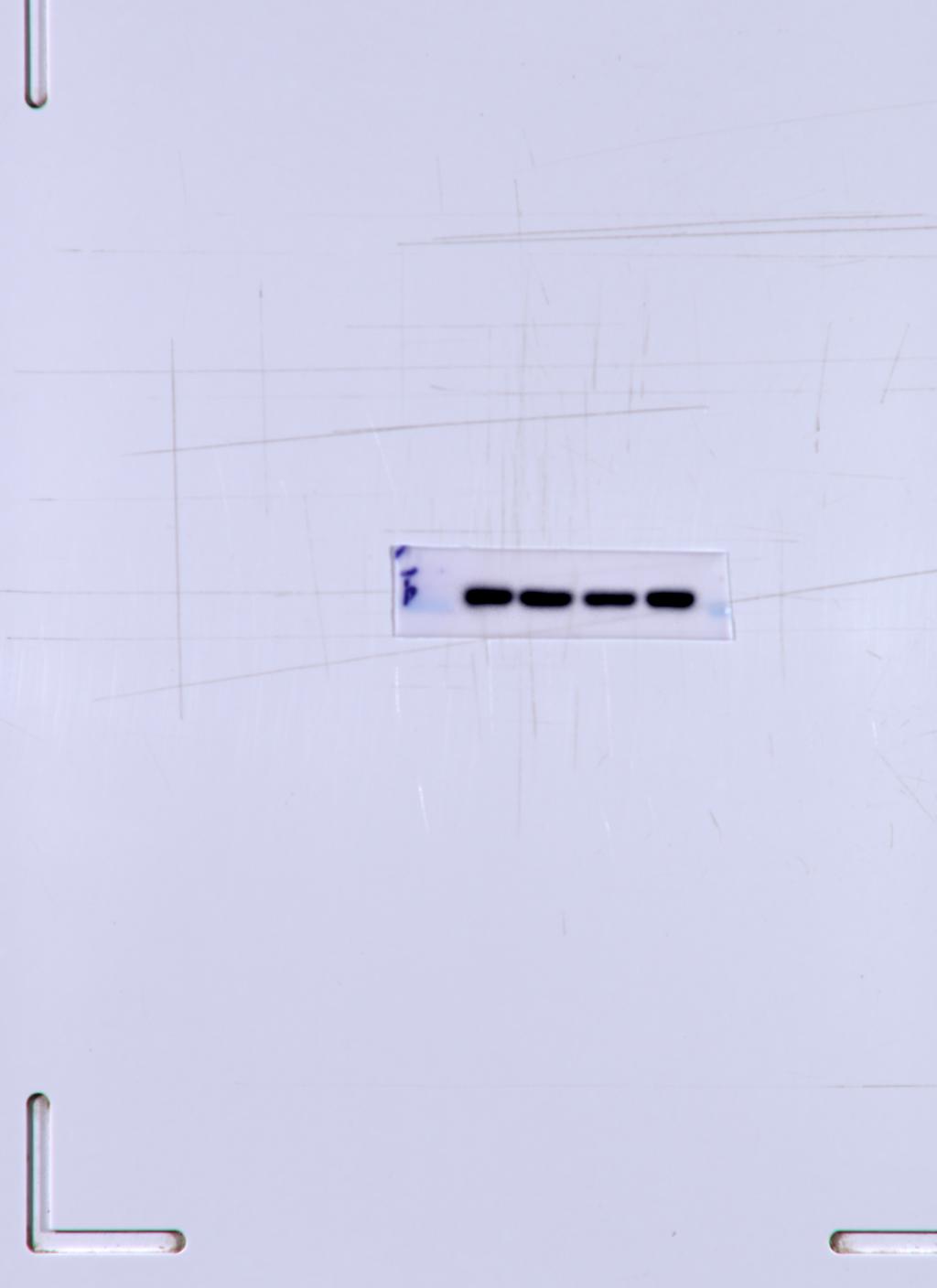
**

| Gray scale | 2.645 | 2.647 | 2.536 | 2.638 |
| --- | --- | --- | --- | --- |

**Fig. 4E~CDV-N**

| hpi | 26 | 30 |
| --- | --- | --- |

**
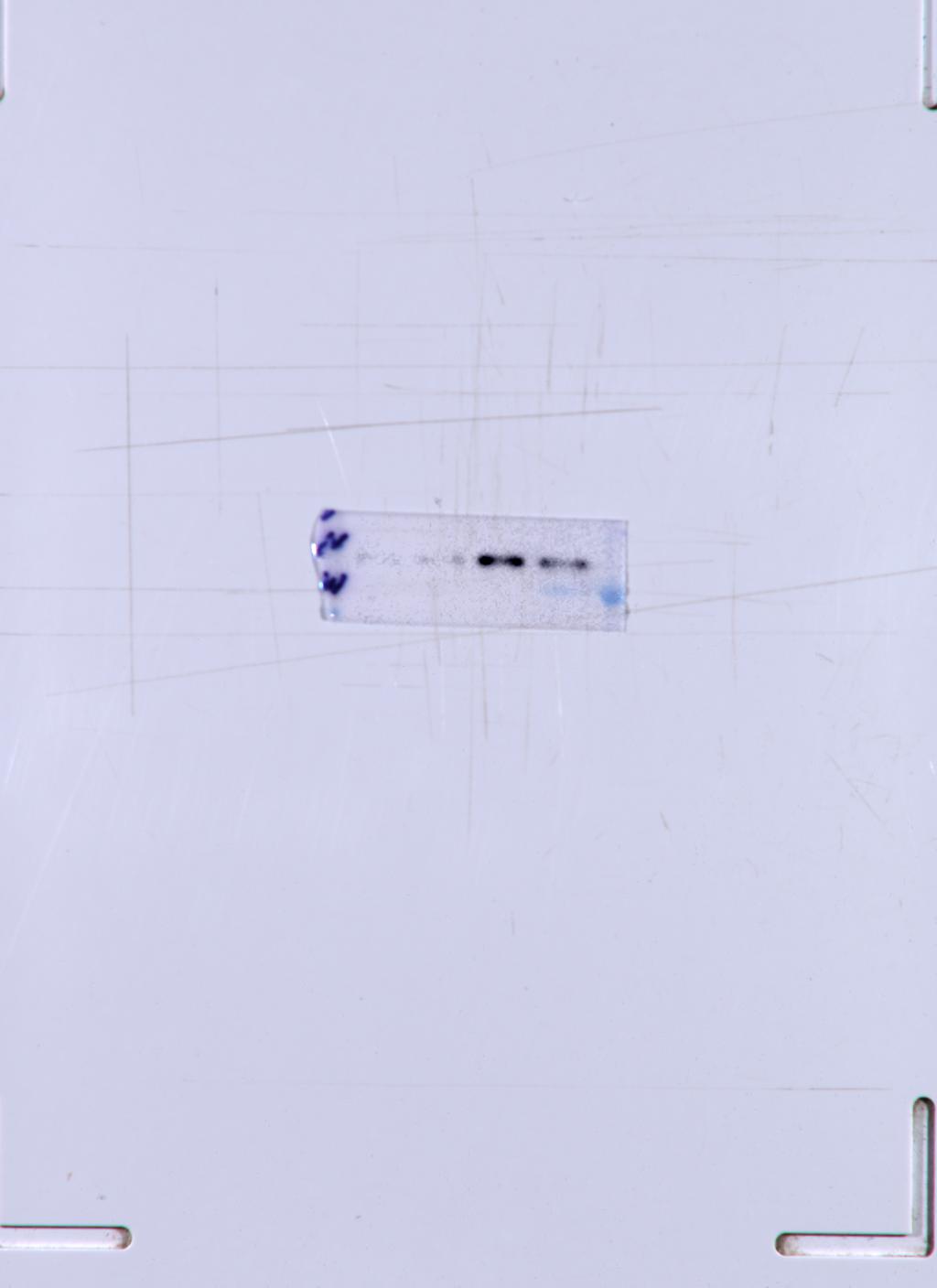
**

| Gray scale | 0.130 | 0.168 | 1.361 | 0.772 |
| --- | --- | --- | --- | --- |

**Fig. 4E~p62**

| hpi | 26 | 30 |
| --- | --- | --- |

**
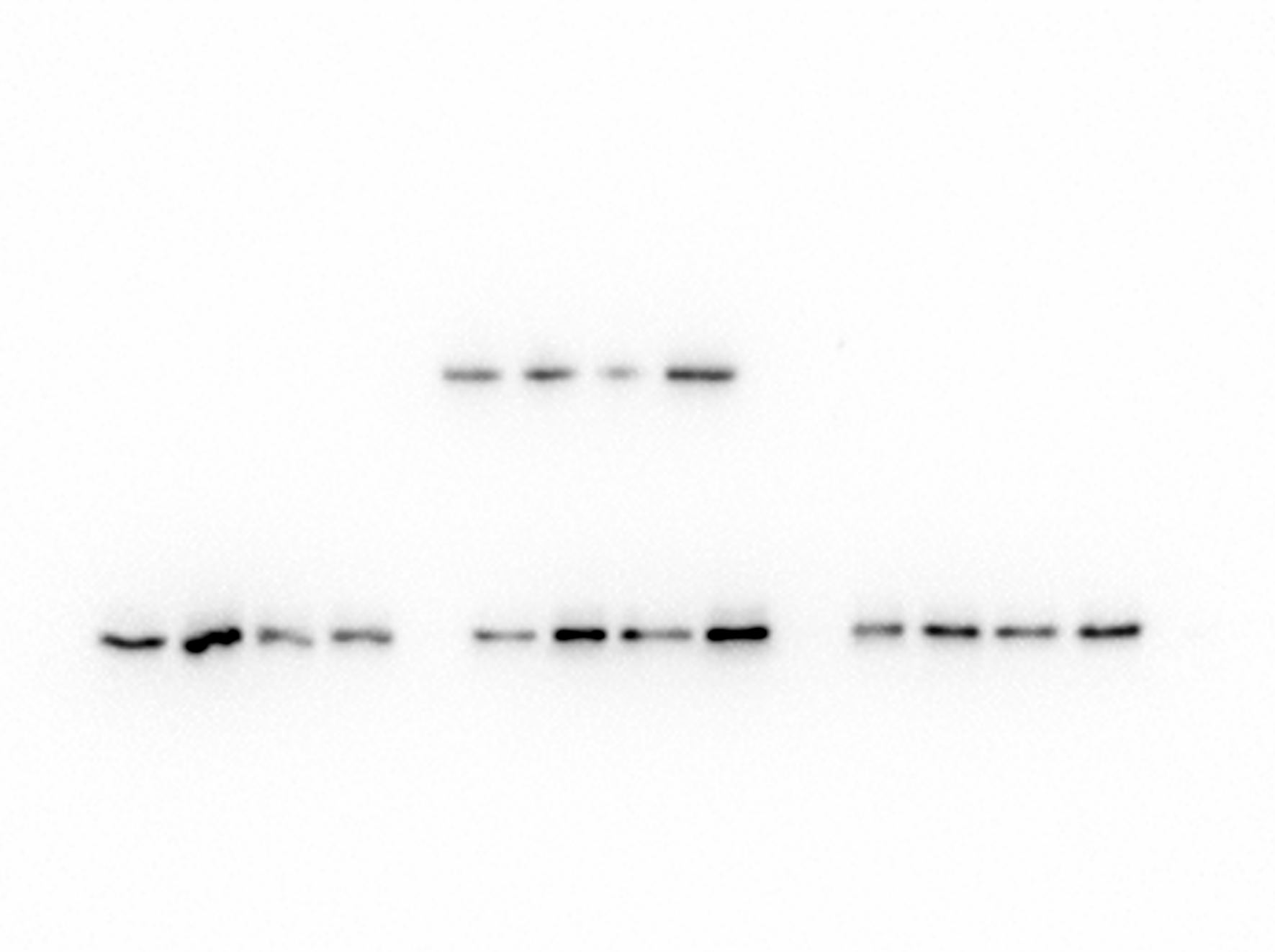
**

| Gray scale | 0.825 | 1.418 | 1.073 | 1.667 |
| --- | --- | --- | --- | --- |

**Fig. 4E~LC3**

| hpi | 26 | 30 |
| --- | --- | --- |

**
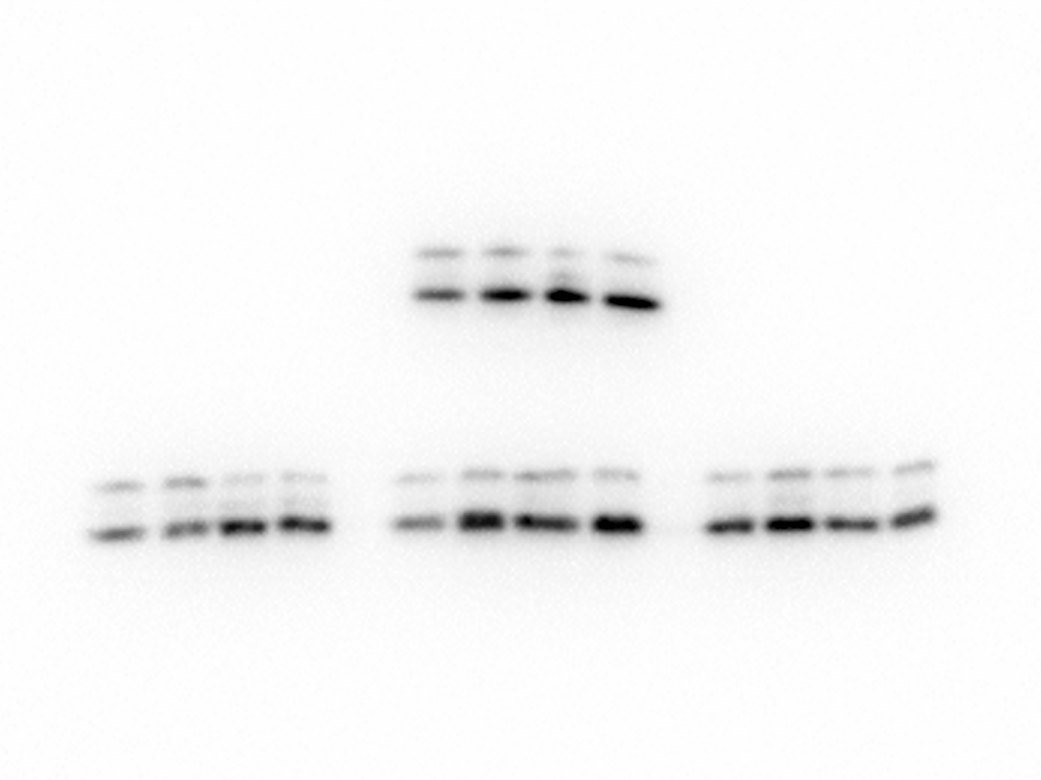
**

| Gray scale | 1.086 | 0.942 | 2.086 | 1.811 |
| --- | --- | --- | --- | --- |

**Fig. 4E~β-tubulin**

| hpi | 26 | 30 |
| --- | --- | --- |

**
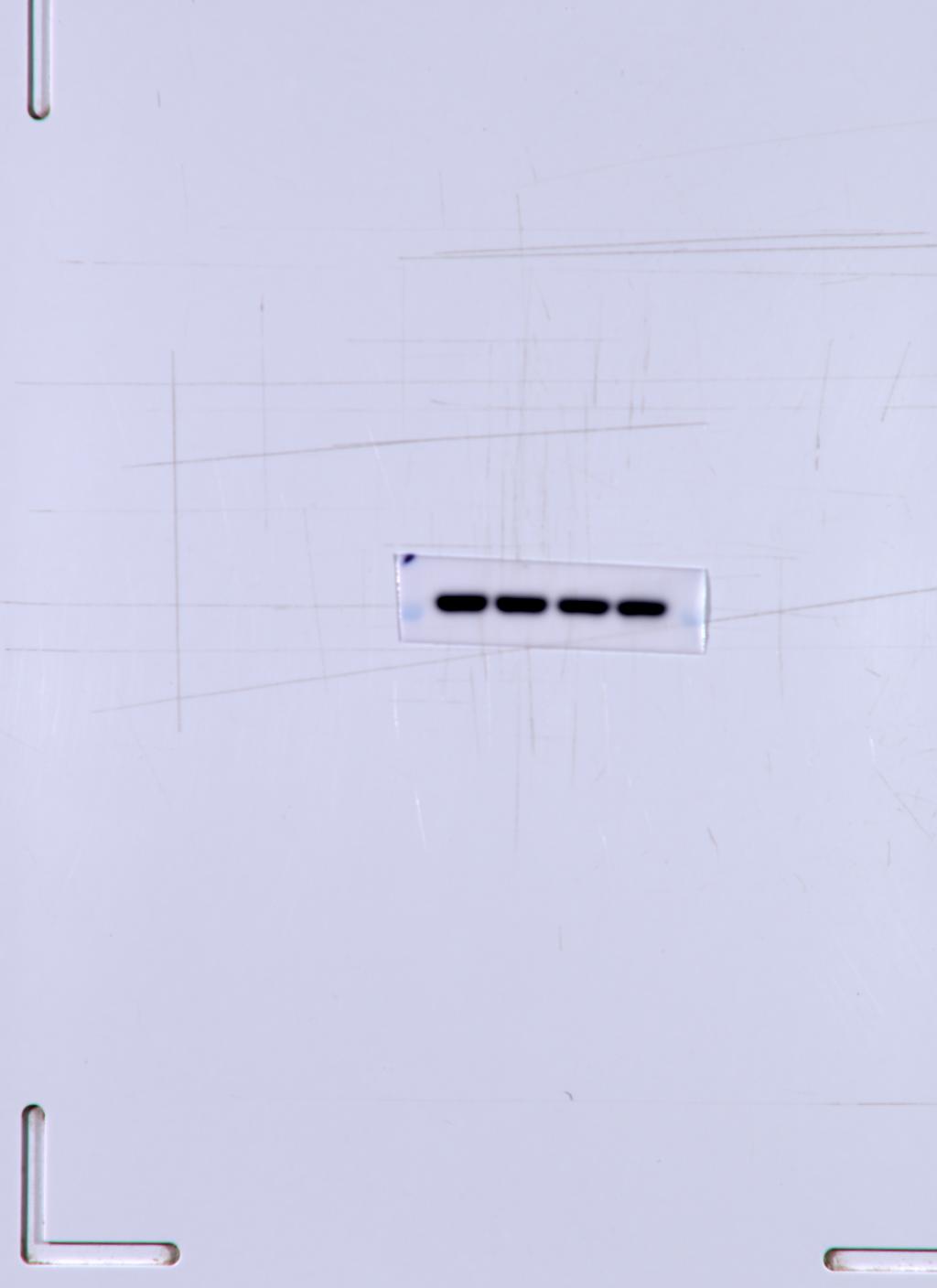
**

| Gray scale | 2.864 | 2.846 | 2.784 | 2.759 |
| --- | --- | --- | --- | --- |

**Fig. 5A~ATG5**

|  | si-  control | si-  ATG5 |
| --- | --- | --- |

**
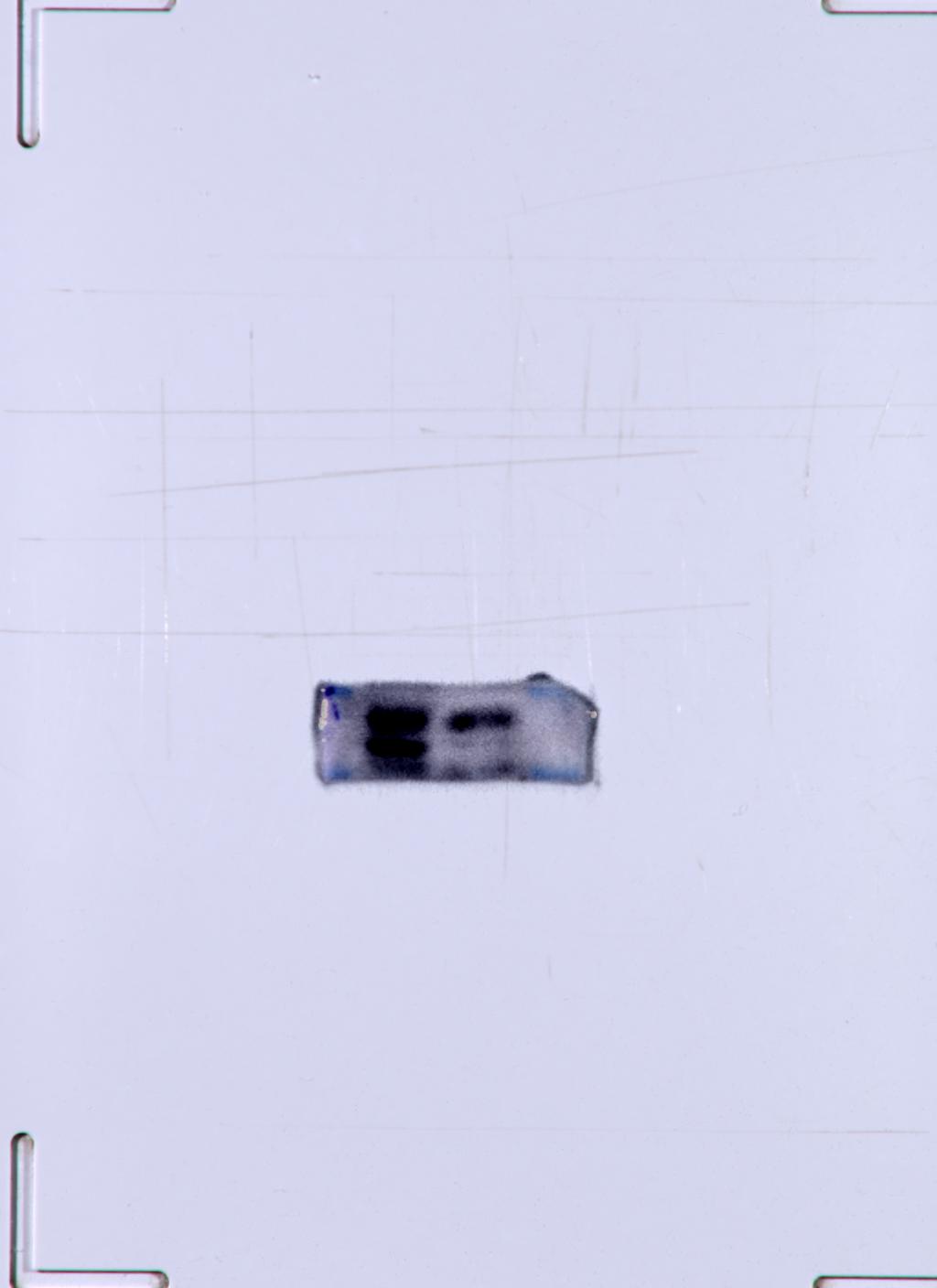
**

| Gray scale | 3.511 | 1.868 |
| --- | --- | --- |

**Fig. 5A~β-tubulin**

|  | si-  control | si-  ATG5 |
| --- | --- | --- |

**
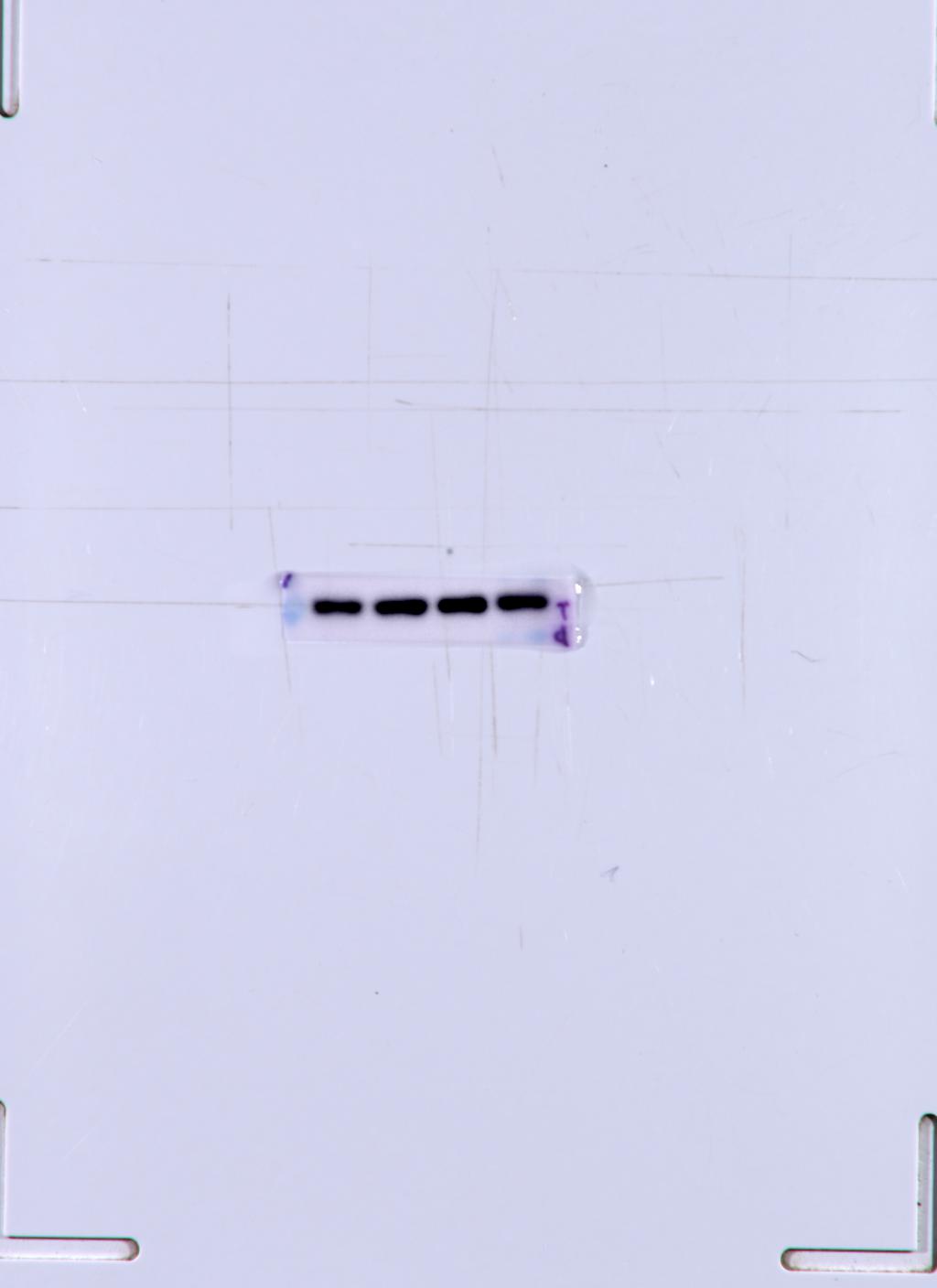
**

| Gray scale | 2.637 | 2.864 |
| --- | --- | --- |

**Fig. 5C~CDV-N**

| hpi | 32 | | 24 | | 28 | | 32 | |
| --- | --- | --- | --- | --- | --- | --- | --- | --- |
|  | si-  control | si-  ATG5 | si-  control | si-  ATG5 | si-  control | si-  ATG5 | si-  control | si-  ATG5 |

**
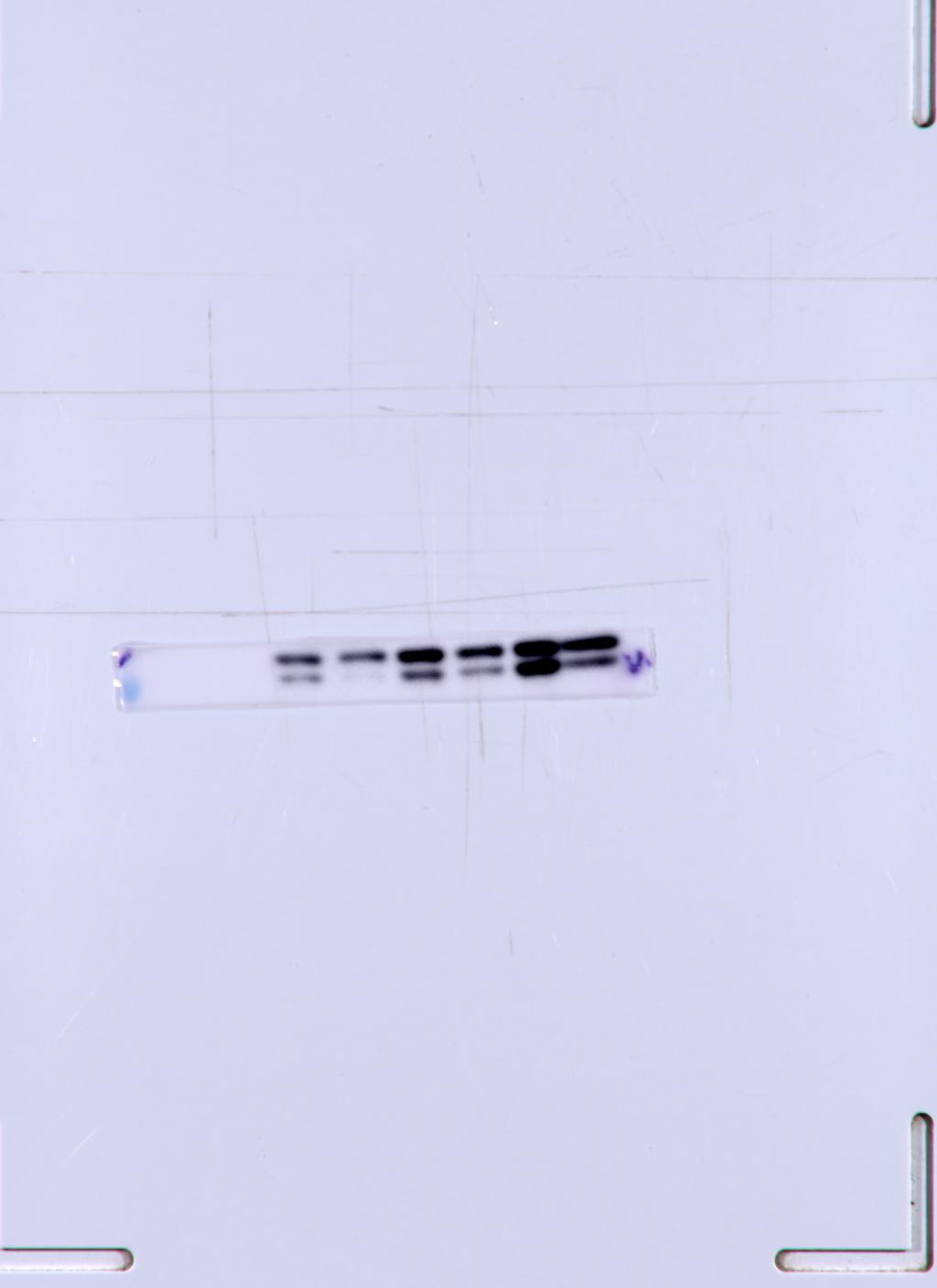
**

| Gray scale |  |  | 1.824 | 1.223 | 3.876 | 2.648 | 5.174 | 3.981 |
| --- | --- | --- | --- | --- | --- | --- | --- | --- |

**Fig. 5C~LC3**

| hpi | 32 | | 24 | | 28 | | 32 | |
| --- | --- | --- | --- | --- | --- | --- | --- | --- |
|  | si-  control | si-  ATG5 | si-  control | si-  ATG5 | si-  control | si-  ATG5 | si-  control | si-  ATG5 |

**
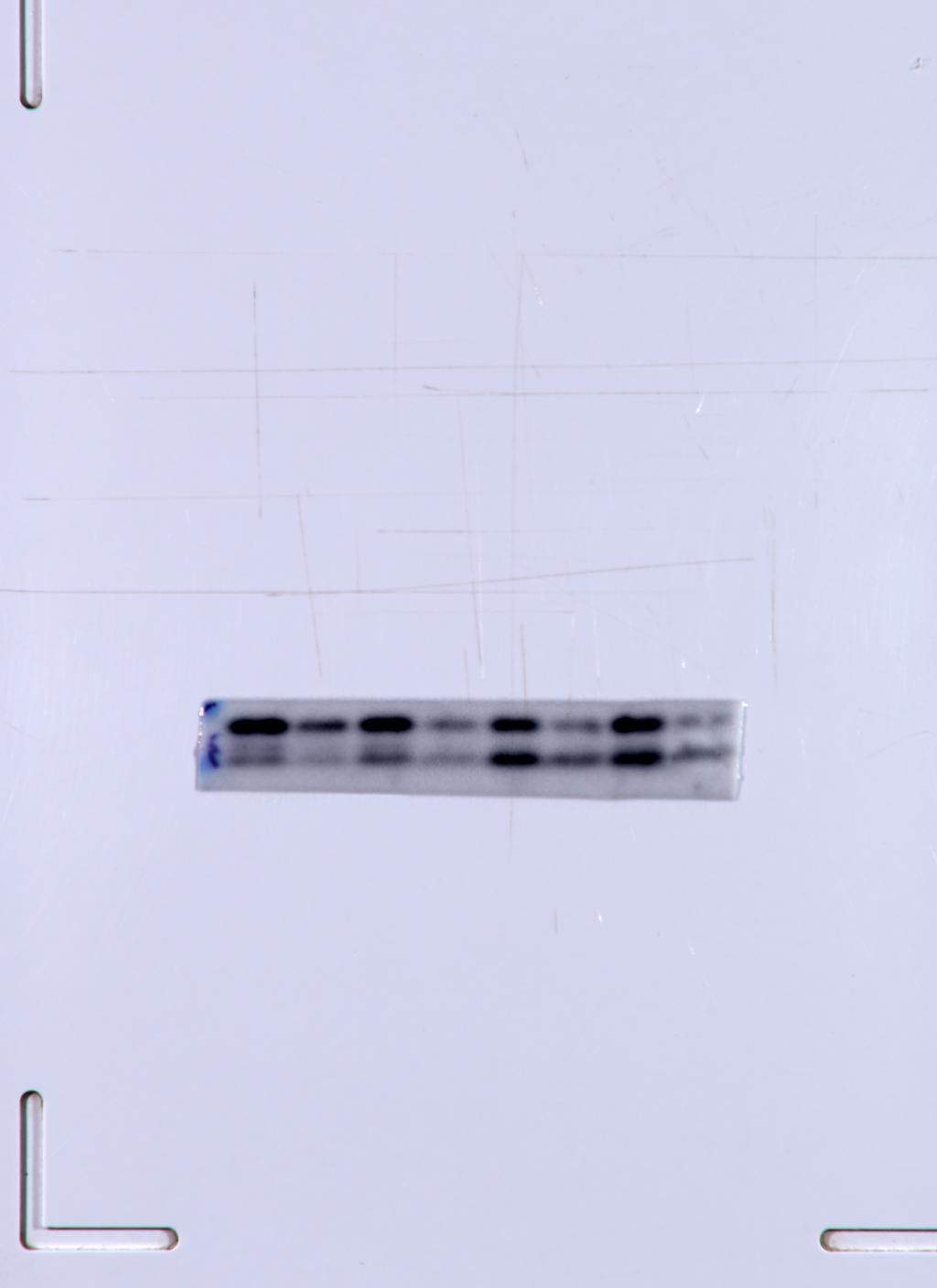
**

| Gray scale |  |  | 0.905 | 0.470 | 1.667 | 0.976 | 1.908 | 0.777 |
| --- | --- | --- | --- | --- | --- | --- | --- | --- |

**Fig. 5C~p62**

| hpi | 32 | | 24 | | 28 | | 32 | |
| --- | --- | --- | --- | --- | --- | --- | --- | --- |
|  | si-  control | si-  ATG5 | si-  control | si-  ATG5 | si-  control | si-  ATG5 | si-  control | si-  ATG5 |

**
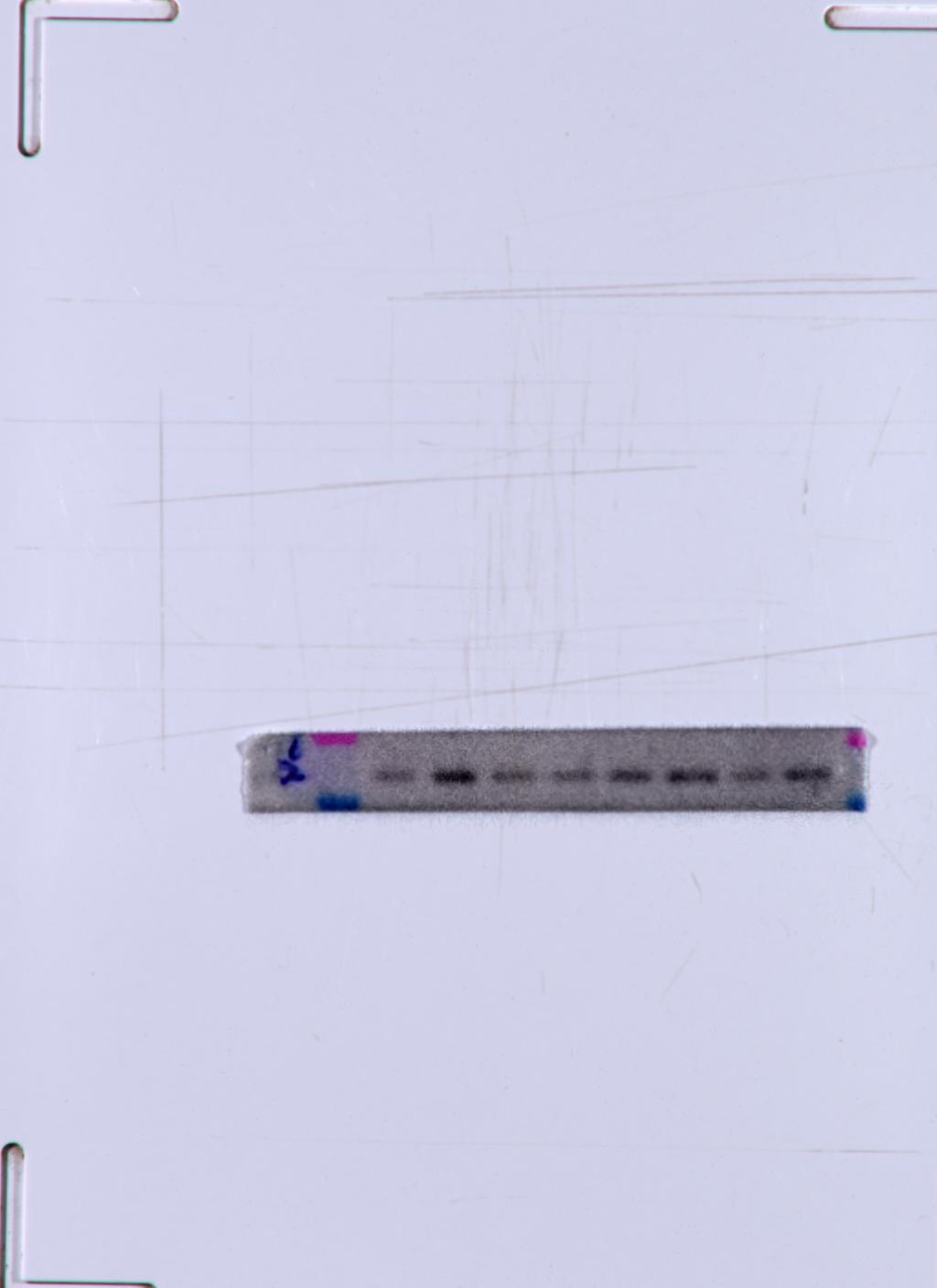
**

**Fig. 5C~β-tubulin**

| hpi | 32 | | 24 | | 28 | | 32 | |
| --- | --- | --- | --- | --- | --- | --- | --- | --- |
|  | si-  control | si-  ATG5 | si-  control | si-  ATG5 | si-  control | si-  ATG5 | si-  control | si-  ATG5 |


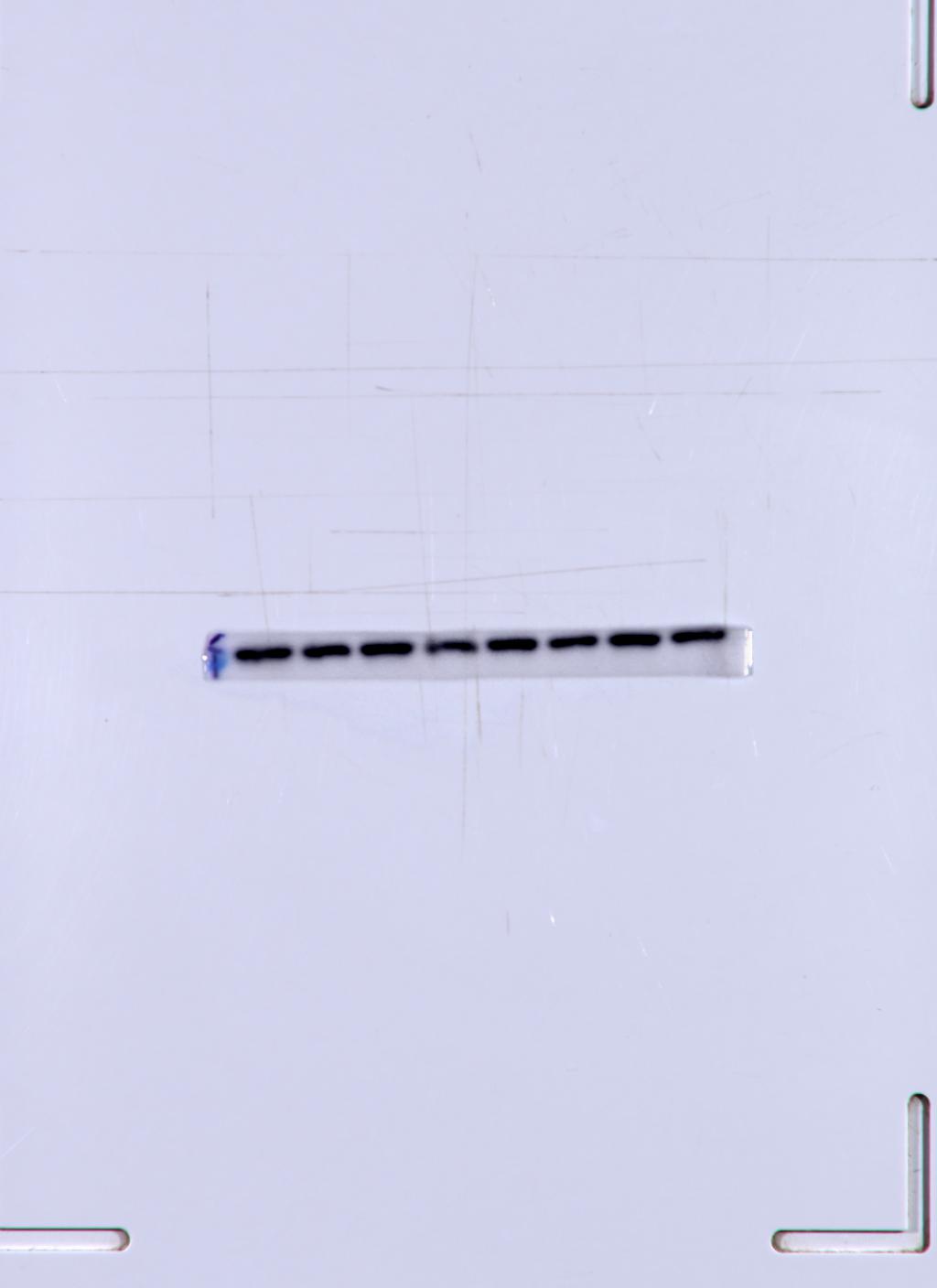


| Gray scale | 2.102 | 1.982 | 1.997 | 1.938 | 1.921 | 1.876 | 1.978 | 1.849 |
| --- | --- | --- | --- | --- | --- | --- | --- | --- |

**Fig. 6C~LC3**

| hpi | 28 | 32 |
| --- | --- | --- |


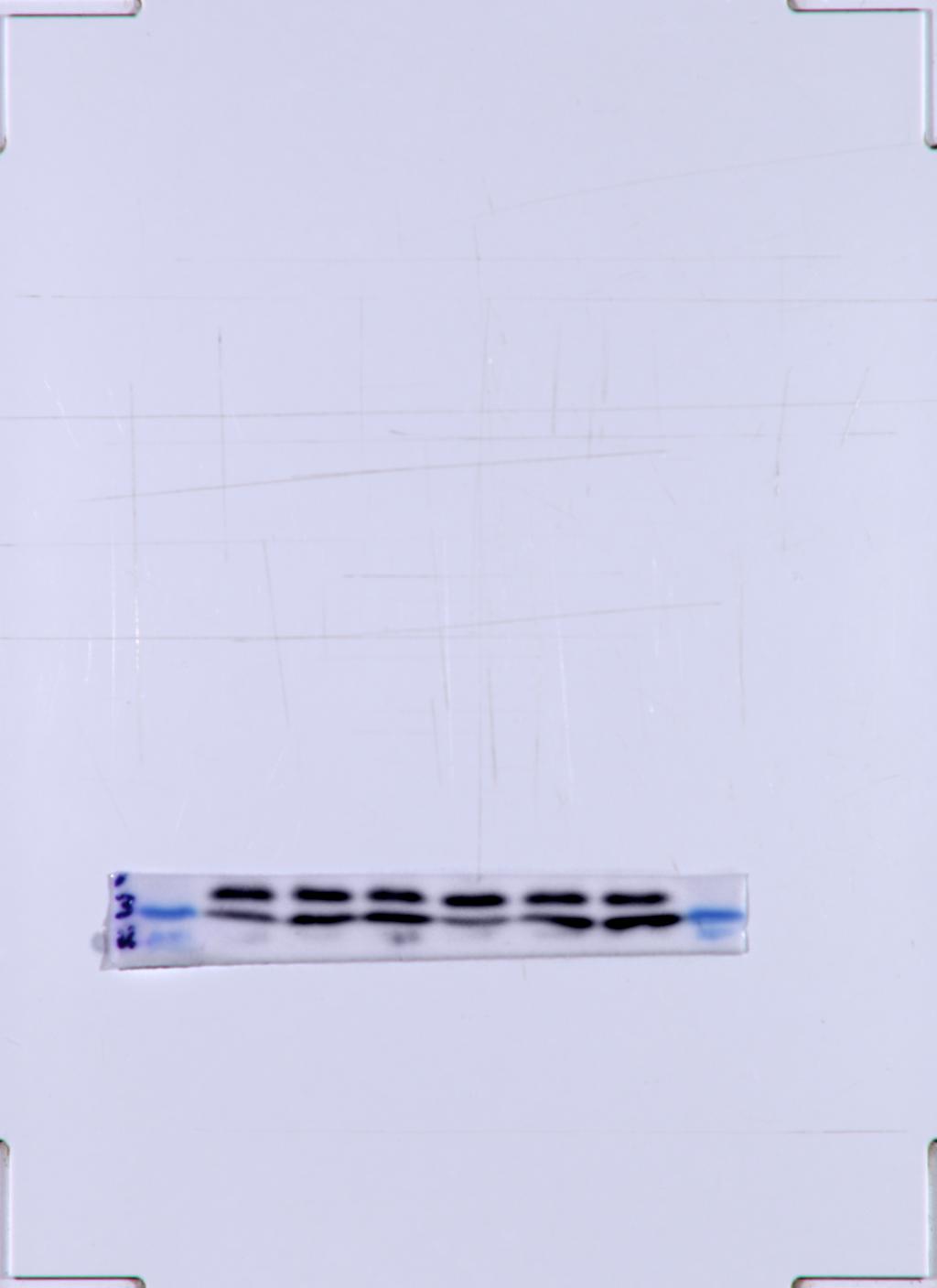


| Gray scale | 1.302 | 2.164 | 2.404 | 1.034 | 2.043 | 2.435 |
| --- | --- | --- | --- | --- | --- | --- |

**Fig. 6C~β-tubulin**

| hpi | 28 | 32 |
| --- | --- | --- |


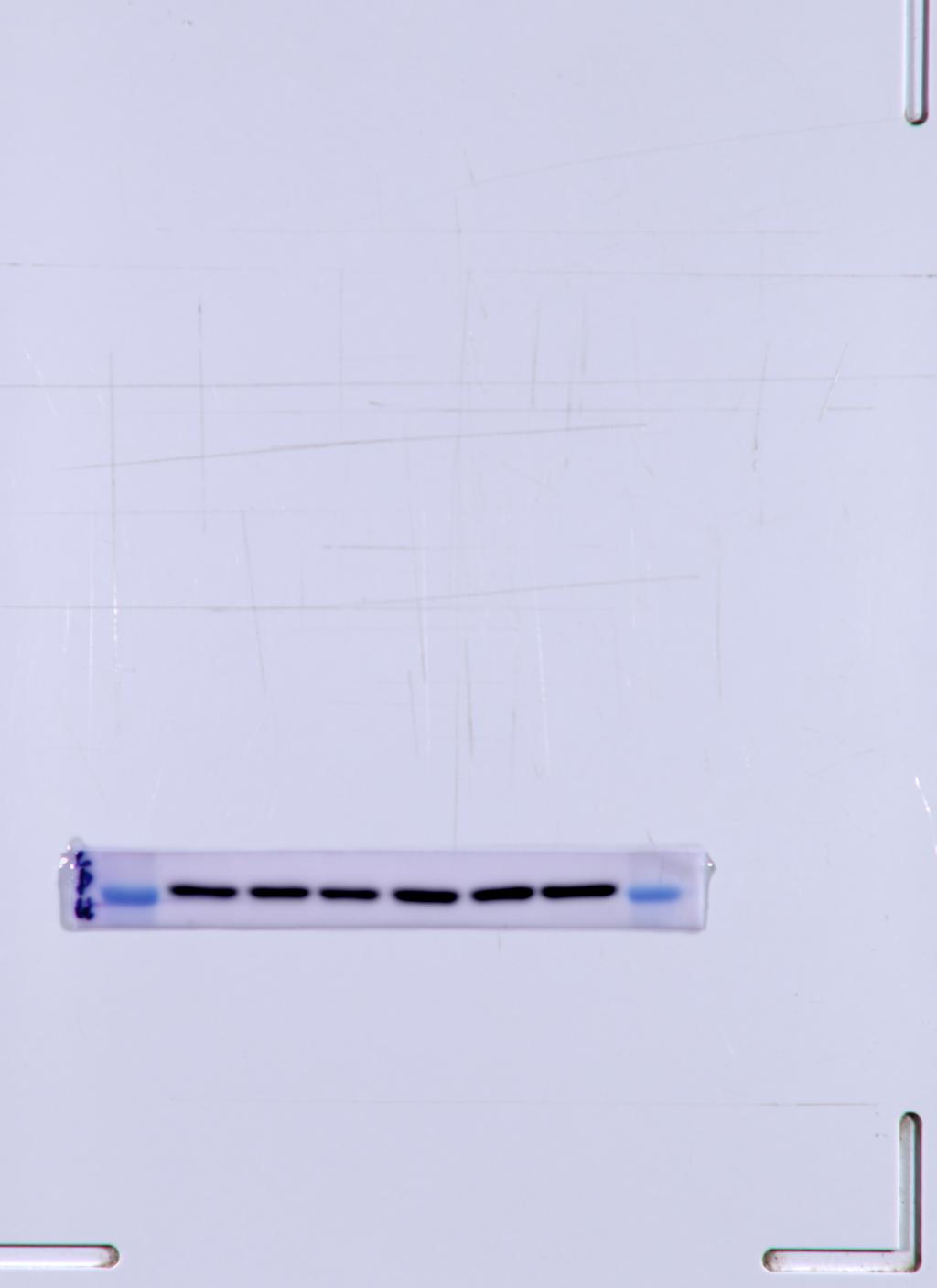


| Gray scale | 2.121 | 2.122 | 2.119 | 2.232 | 2.231 | 2.234 |
| --- | --- | --- | --- | --- | --- | --- |

**Fig. 6E~Flag**

mock

p3×Flag-CMV

p3×Flag-CMV-N


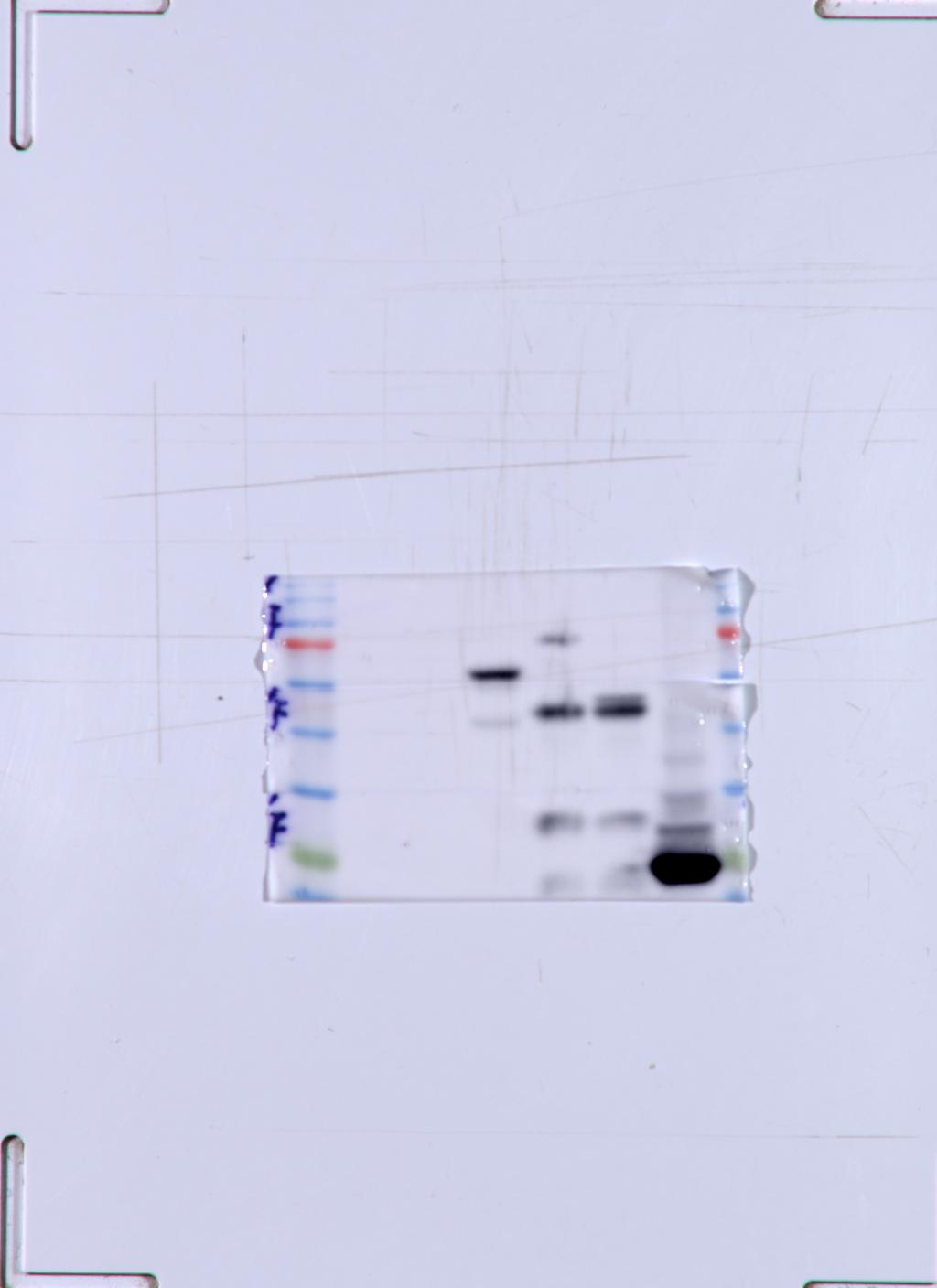


**Fig. 6E~β-tubulin**

mock

p3×Flag-CMV

p3×Flag-CMV-N


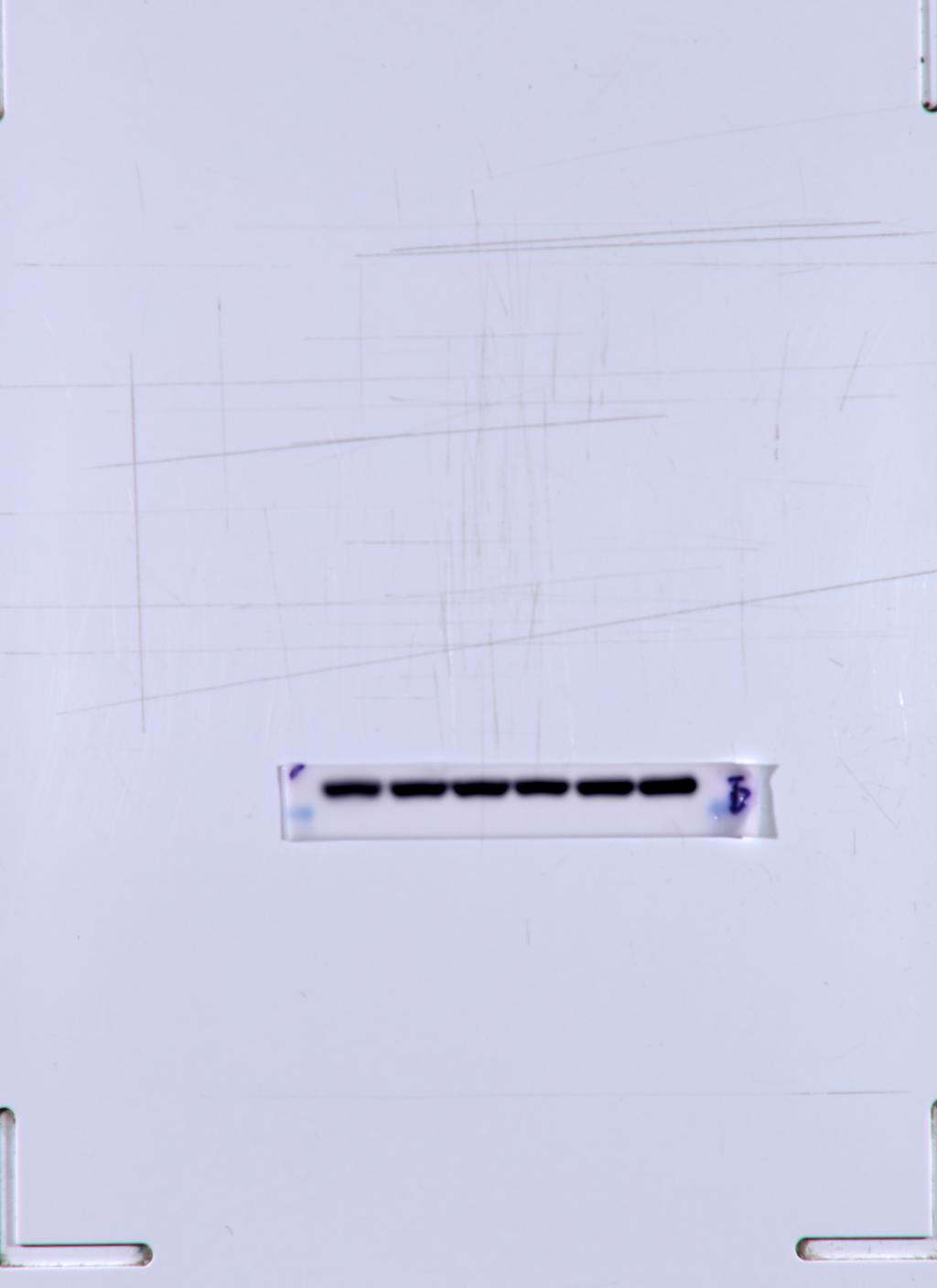


**Fig. 6F~p-mTOR**


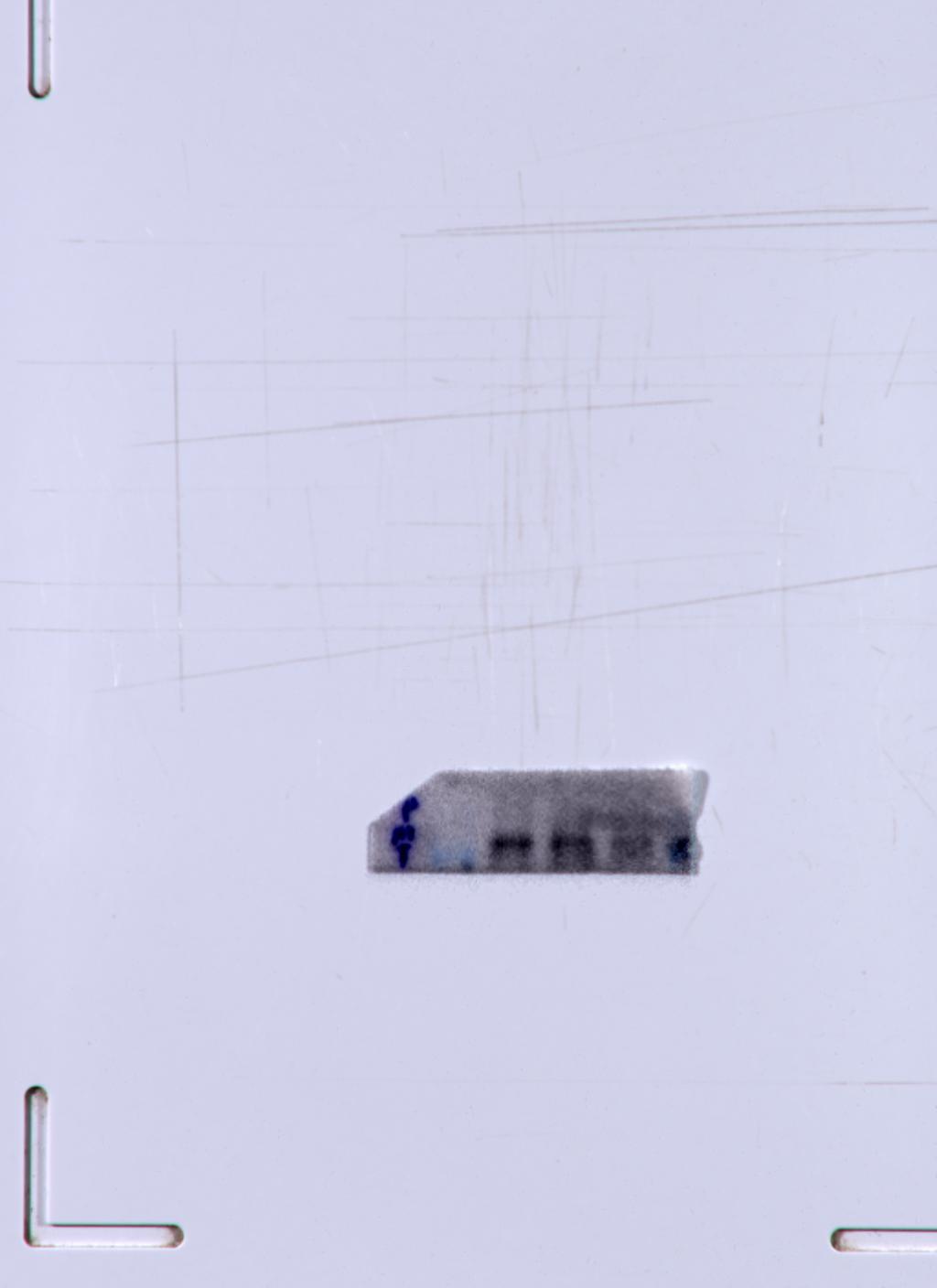


| Gray scale | 0.563 | 0.579 | 0.314 |
| --- | --- | --- | --- |

**Fig. 6F~mTOR**


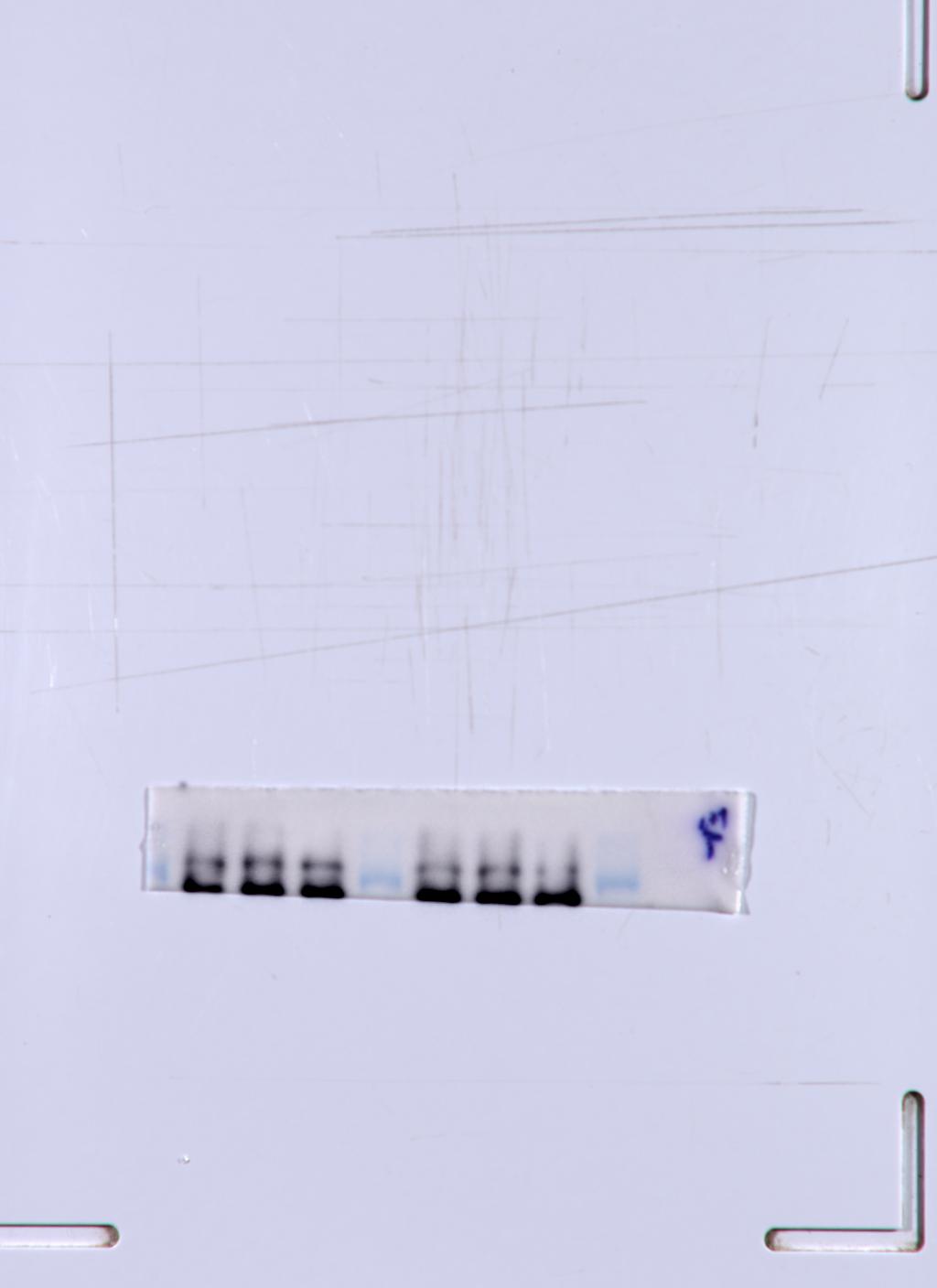


| Gray scale | 1.730 | 2.025 | 2.026 |
| --- | --- | --- | --- |

**Fig. 6F~p-AKT**


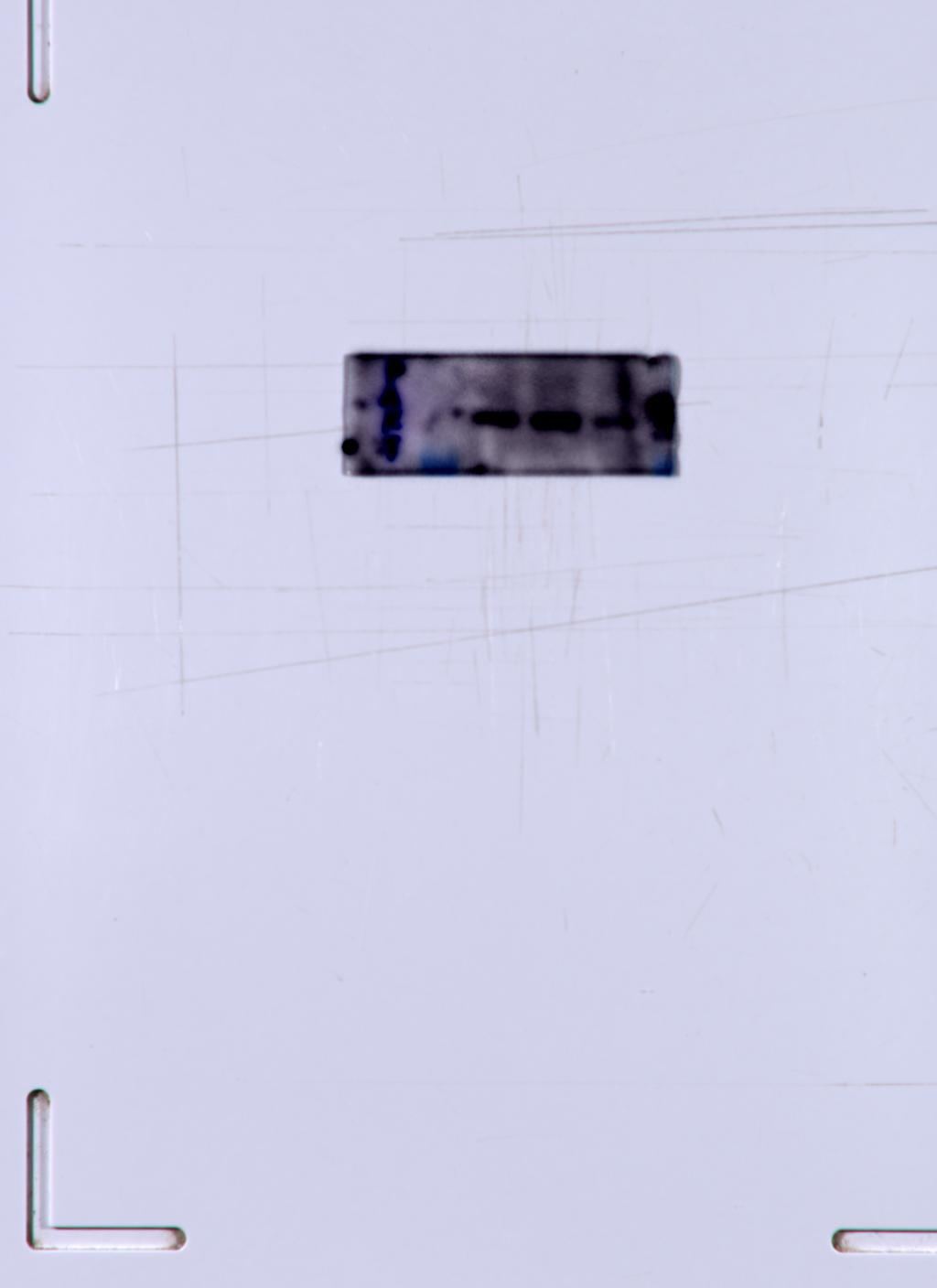


| Gray scale | 2.284 | 2.674 | 1.599 |
| --- | --- | --- | --- |

**Fig. 6F~AKT**


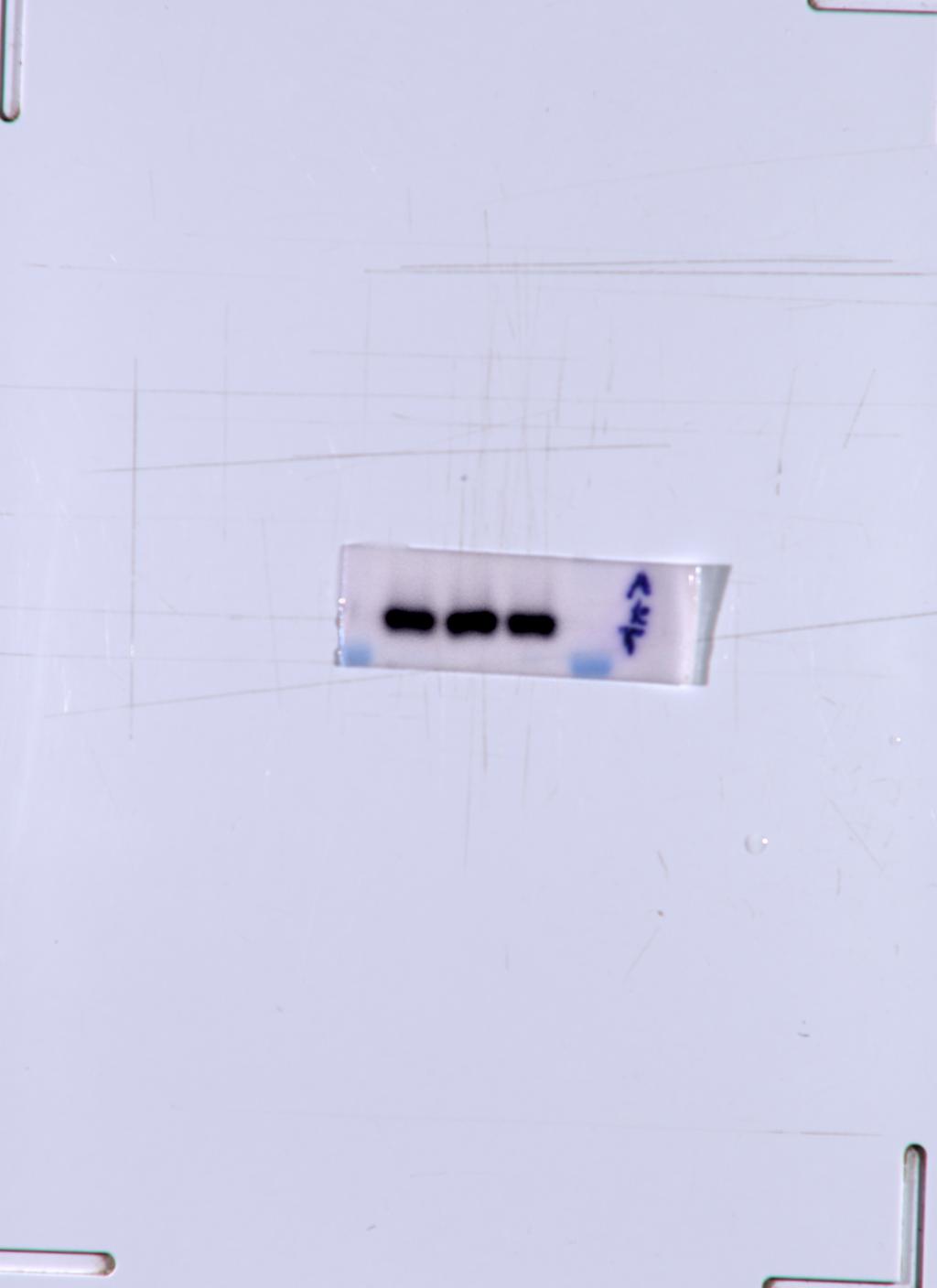


| Gray scale | 2.573 | 2.626 | 2.574 |
| --- | --- | --- | --- |

**Fig. 6F~p62**


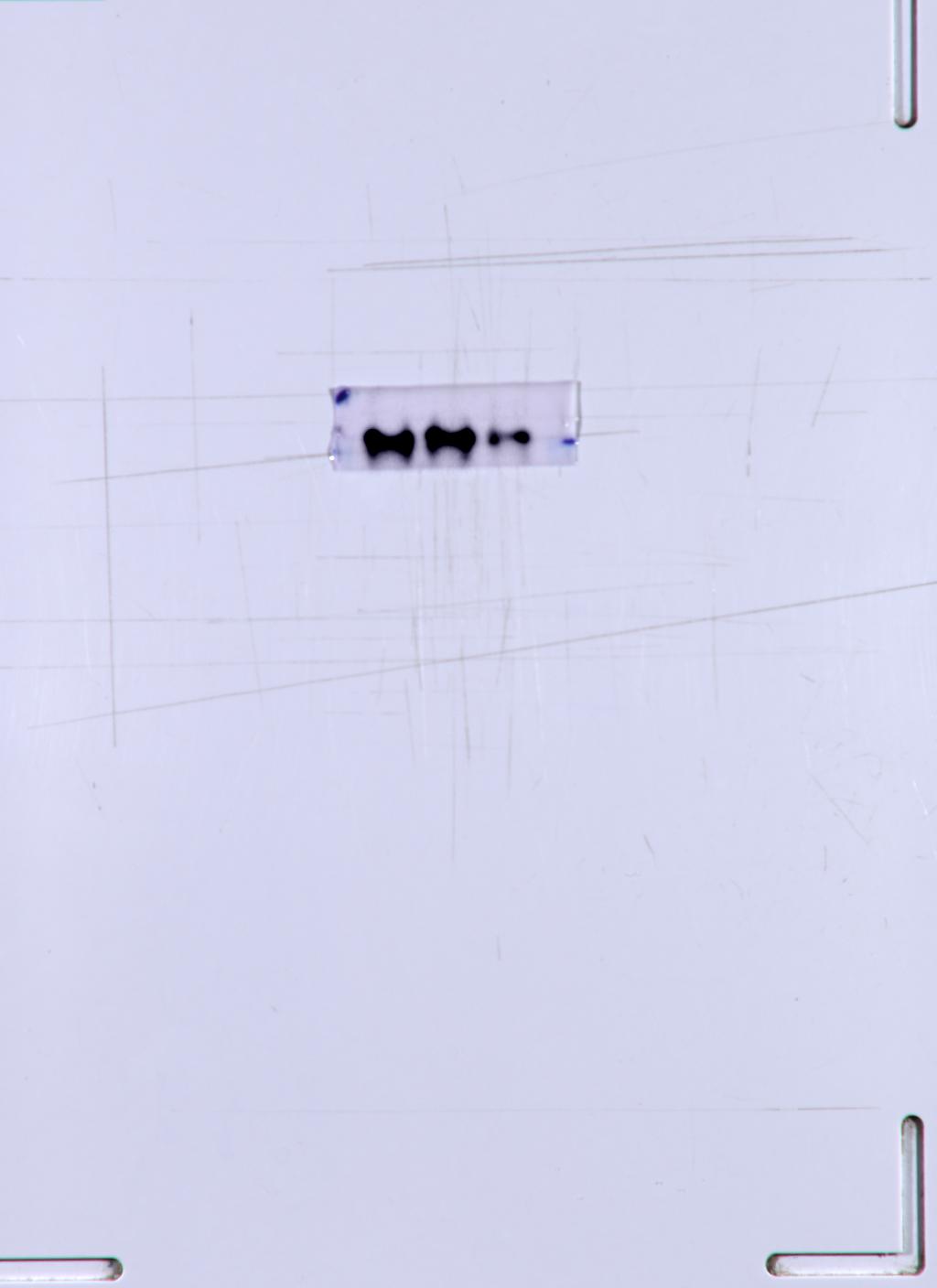


| Gray scale | 4.181 | 3.599 | 1.677 |
| --- | --- | --- | --- |

**Fig. 6F~LC3**


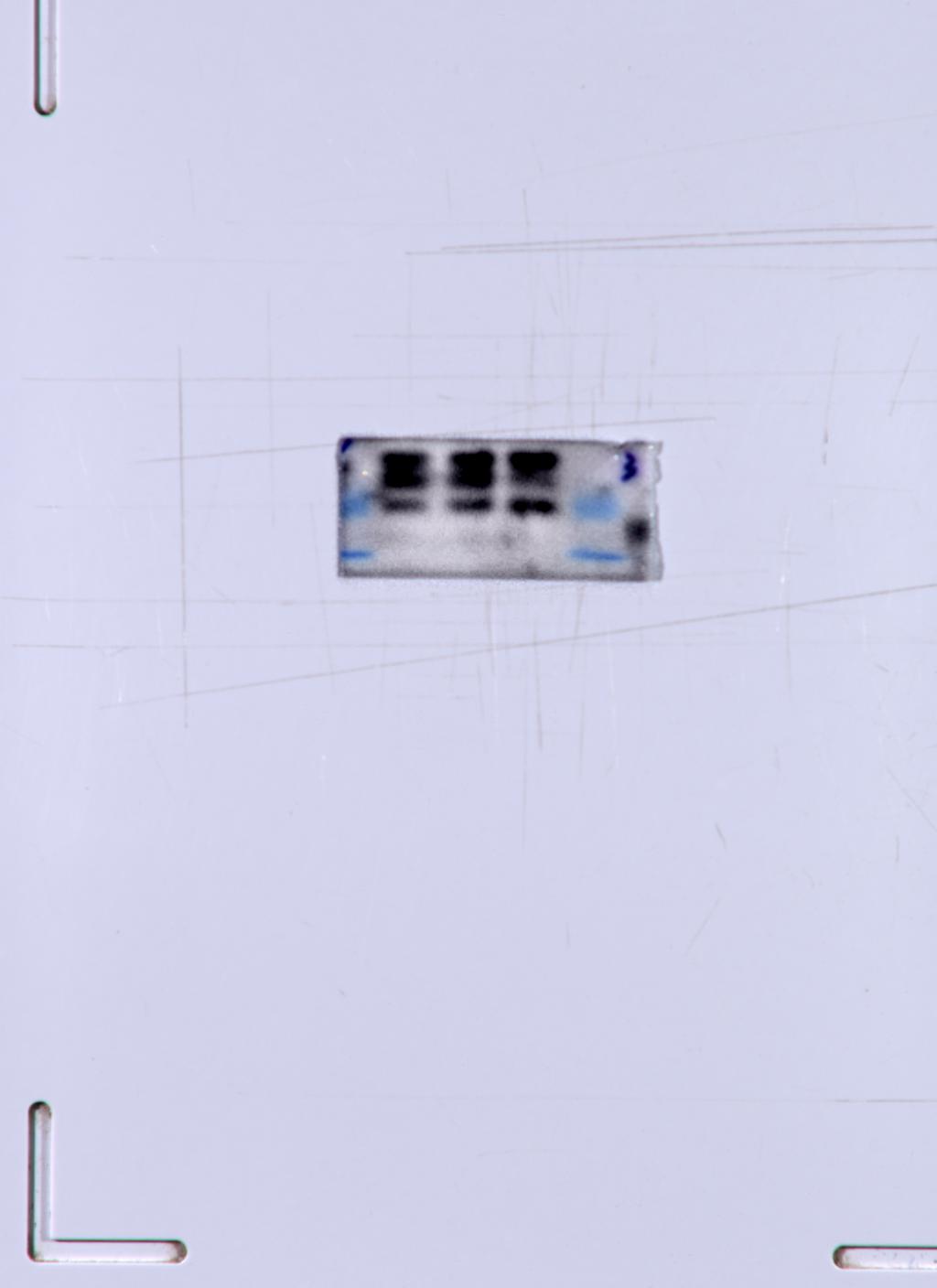


| Gray scale | 0.803 | 1.031 | 1.632 |
| --- | --- | --- | --- |

**Fig. 6F~β-tubulin**


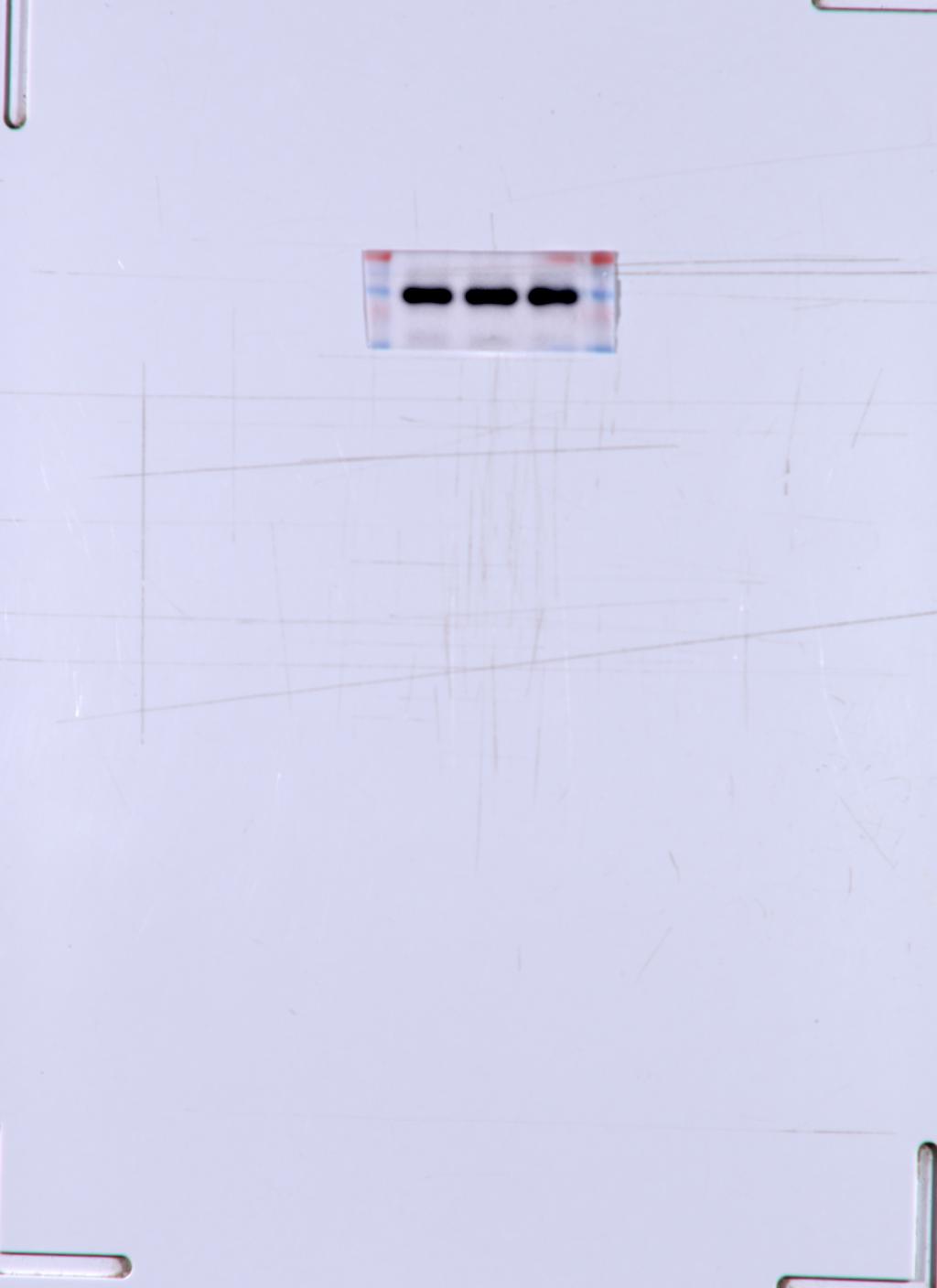


| Gray scale | 2.638 | 2.642 | 2.633 |
| --- | --- | --- | --- |

**Fig. S2A~Flag**

p3×Flag-CMV

mock

p3×Flag-CMV-C

p3×Flag-CMV-V

p3×Flag-CMV-P

p3×Flag-CMV-N


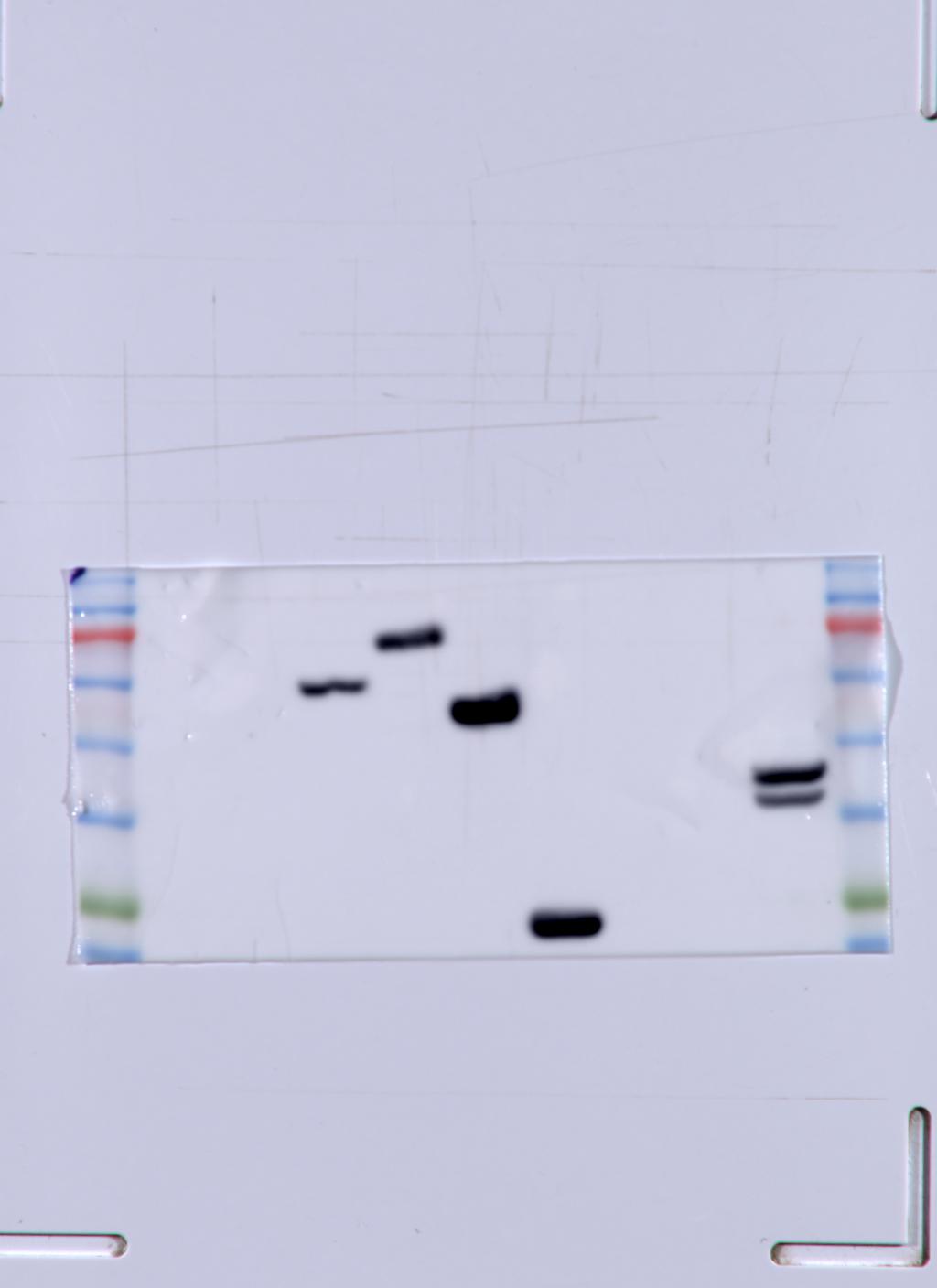


**Fig. S2A~β-tubulin**


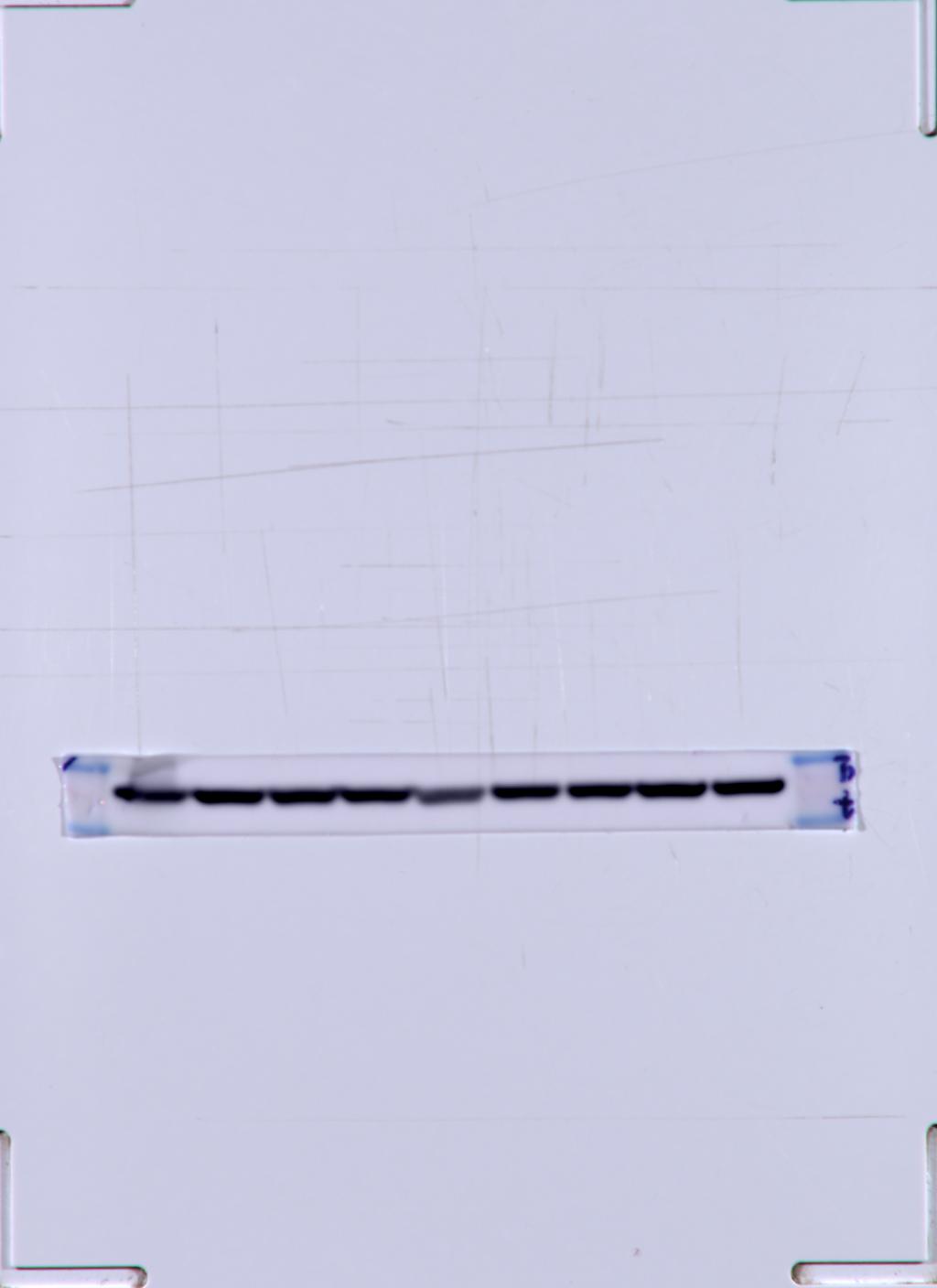


**Fig. S2B~LC3**


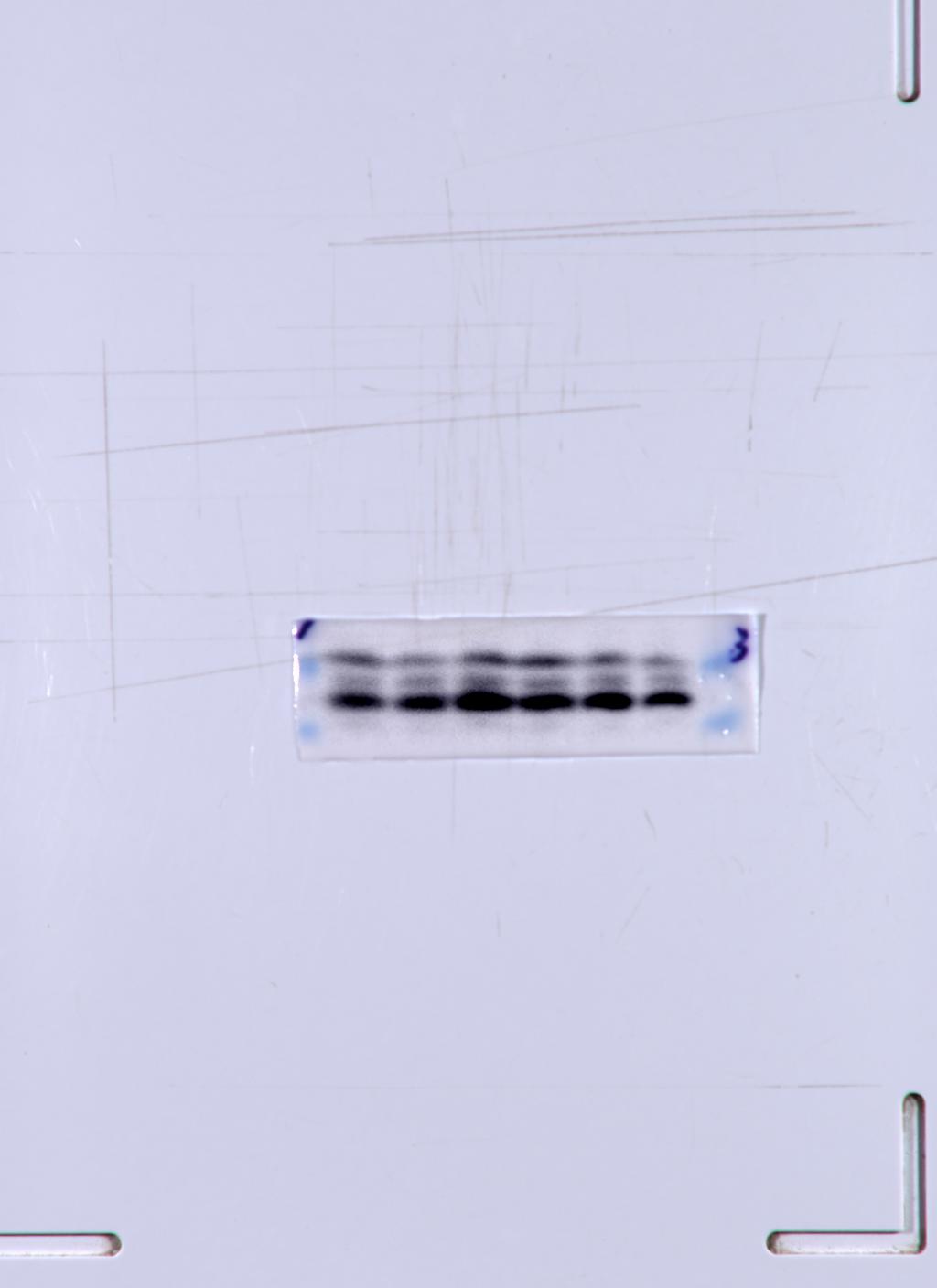


**Fig. S2B~β-tubulin**

| hpi | mock | Flag | -N | -  P | -  V | -C |
| --- | --- | --- | --- | --- | --- | --- |


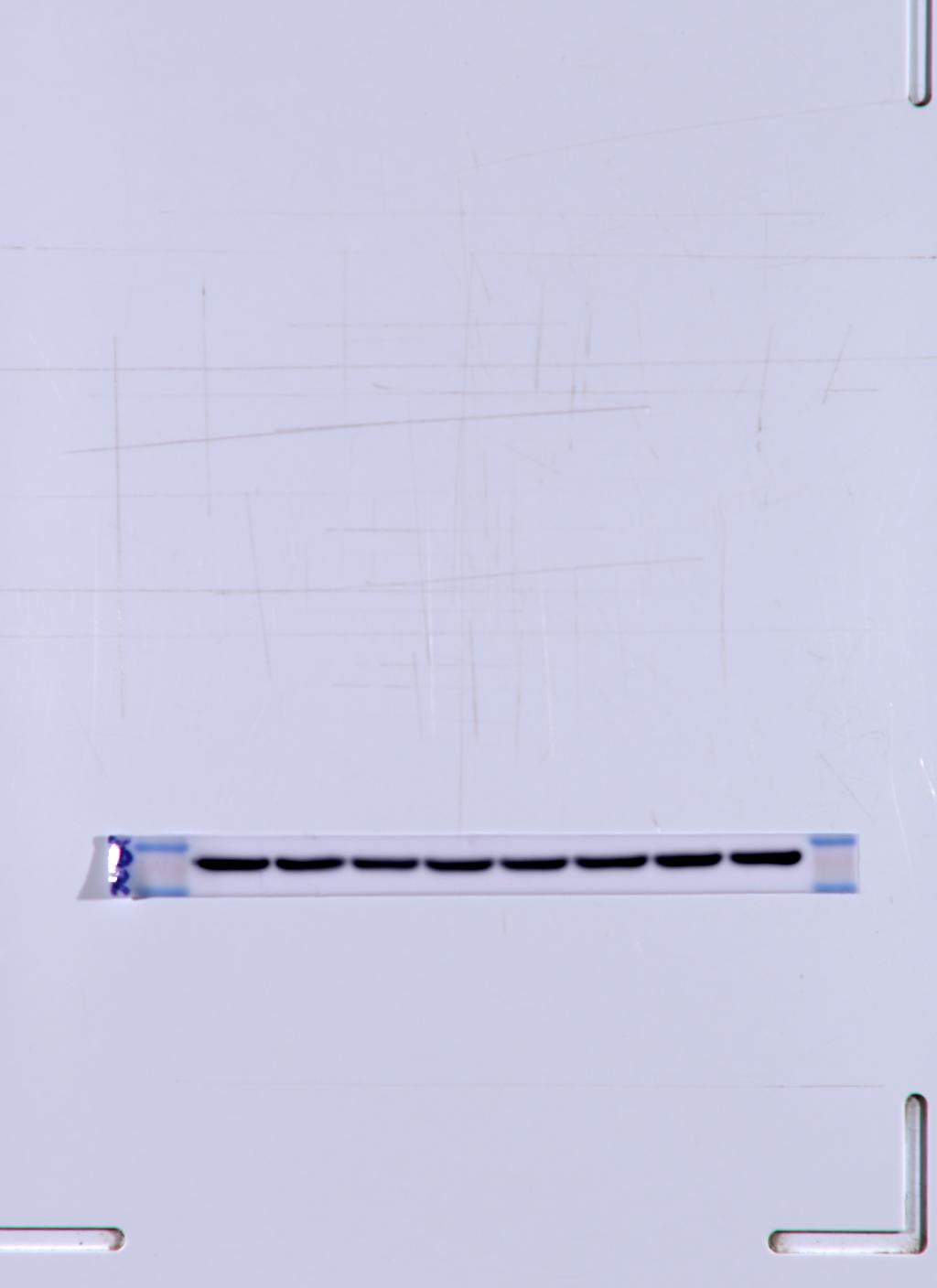

Supplement: Supplementary file 2 — Additional file 2. The original blots for the figures. [file 12917_2023_3575_MOESM2_ESM.docx]
